# New pleiotropic effects of eliminating a rare tRNA from *Streptomyces coelicolor*, revealed by combined proteomic and transcriptomic analysis of liquid cultures

**DOI:** 10.1186/1471-2164-8-261

**Published:** 2007-08-02

**Authors:** Andy Hesketh, Giselda Bucca, Emma Laing, Fiona Flett, Graham Hotchkiss, Colin P Smith, Keith F Chater

**Affiliations:** 1 Department of Molecular Microbiology, John Innes Centre, Norwich Research Park, Colney, Norwich, NR4 7UH, UK; 2 School of Biomedical and Molecular Sciences, University of Surrey, Guildford, Surrey, GU2 7XH, UK; 3 Manchester Interdisciplinary Biocentre, The University of Manchester, 131 Princess Street, Manchester, M1 7ND, UK

## Abstract

**Background:**

In *Streptomyces coelicolor*, *bldA *encodes the only tRNA for a rare leucine codon, UUA. This tRNA is unnecessary for growth, but is required for some aspects of secondary metabolism and morphological development. We describe a transcriptomic and proteomic analysis of the effects of deleting *bldA *on cellular processes during submerged culture: conditions relevant to the industrial production of antibiotics.

**Results:**

At the end of rapid growth, a co-ordinated transient up-regulation of about 100 genes, including many for ribosomal proteins, was seen in the parent strain but not the Δ*bldA *mutant. Increased basal levels of the signal molecule ppGpp in the mutant strain may be responsible for this difference. Transcripts or proteins from a further 147 genes classified as *bldA*-influenced were mostly expressed late in culture in the wild-type, though others were significantly transcribed during exponential growth. Some were involved in the biosynthesis of seven secondary metabolites; and some have probable roles in reorganising metabolism after rapid growth. Many of the 147 genes were "function unknown", and may represent unknown aspects of *Streptomyces *biology. Only two of the 147 genes contain a TTA codon, but some effects of *bldA *could be traced to TTA codons in regulatory genes or polycistronic operons. Several proteins were affected post-translationally by the *bldA *deletion. There was a statistically significant but weak positive global correlation between transcript and corresponding protein levels. Different technical limitations of the two approaches were a major cause of discrepancies in the results obtained with them.

**Conclusion:**

Although deletion of *bldA *has very conspicuous effects on the gross phenotype, the *bldA *molecular phenotype revealed by the "dualomic" approach has shown that only about 2% of the genome is affected; but this includes many previously unknown effects at a variety of different levels, including post-translational changes in proteins and global cellular physiology.

## Background

The extraordinary virtuosity of streptomycetes as producers of secondary metabolites, including most of the antibiotics and anti-cancer agents in wide use, is associated with the developmental complexity of *Streptomyces *colonies, which have a vegetative substrate mycelium and a spore-bearing aerial mycelium. Among many mutants of the model species *Streptomyces coelicolor *with pleiotropic defects in both aspects, this paper focuses on a mutant deleted for the *bldA *locus, which specifies the only tRNA in the genome able to efficiently translate the rare leucine codon UUA [1-3]. Since the complex *bldA *phenotype is likely to involve elements of both transcriptional and translational control, it is particularly suitable for an integrated functional genomics approach. With the aim of discovering consequences of *bldA *mutation that are not obvious to the naked eye, we set out to analyse the RNA and protein extracted from a series of liquid cultures of isogenic *bldA*^+ ^and *bldA *deleted strains. This allowed us to focus on the effects of *bldA *deletion on metabolism and overall cell physiology without the complication of the unsynchronised morphological differentiation observed with surface-grown *S. coelicolor *cultures. Our results revealed unexpected effects of *bldA *mutation on gene expression during growth and the transition phase that precedes entry into stationary phase, as well as more wide-ranging effects on secondary metabolism than previously suspected, and led to further exploration of some of the underpinning mechanisms. Different, but complementary, results were obtained with the two "omic"approaches, and we conclude that there are significant benefits from their combined application.

## Results

### General characterisation of changes in the transcriptome and proteome during liquid culture of strain M600 ("wild-type") and its *bldA* deletion derivative

#### Overall strategy

The genome sequence of *S. coelicolor *[4] had been determined with DNA from a widely-used plasmid-free derivative (M145) of the wild-type strain A3(2). We could not use M145 for the work described here, because of the unexpected finding that *bldA *mutants cannot readily be constructed in M145 (M. Tao and KFC, unpublished; Gehring *et al*. [5]). Instead, we used M600, a plasmid-free prototrophic strain that was derived from the original wild-type strain A3(2) by a minimally mutated route, and has been used for physiological studies [6]. It proved straightforward to construct a *bldA *deletion in M600.

**Figure 1:**
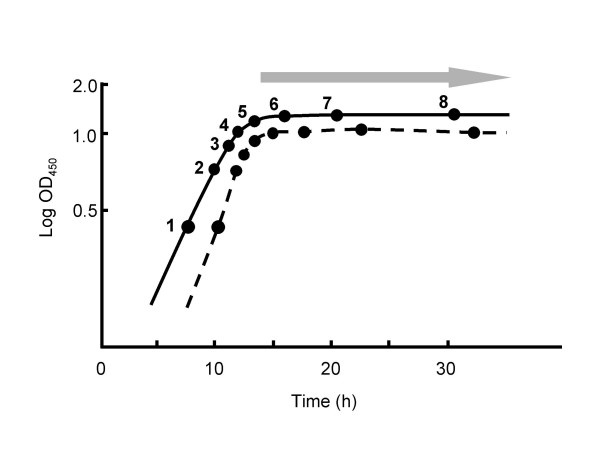
Growth of *S. coelicolor *M600 and M600 Δ*bldA *in supplemented minimal medium. A schematic representation of the measured growth curves is shown with M600 denoted by a solid line, and M600 Δ*bldA *by a broken line. Points 1–8 indicate times when cultures were harvested for RNA and protein extraction, and the shaded arrow represents the onset of pigmented antibiotic production in M600 (the mutant did not produce pigments). All eight sampling points were analysed transcriptomically, while only samples 1, 2, 4, 6 and 8 were subjected to proteomic analysis.

Proteomic and transcriptomic analyses were done with cultures grown in casaminoacid-supplemented liquid minimal medium (SMM) to different stages (Fig. 1). Material from each culture was divided into two parts, which were processed separately for the extraction and characterisation of protein and RNA. All transcriptome experiments were done using three independent time series for each strain (biological replicates), and two of these replicate sets of samples were used for the proteomic analysis. For transcriptome analysis, genomic DNA labelled with Cy-5 dCTP was used as a common reference. This strategy allows the *magnitude *of expression to be estimated, rather than the *relative *expression level that is obtained from 'direct comparison' cDNA vs cDNA hybridisations. This enabled us to compare the level of transcript for each gene with the abundance of its corresponding protein product. The approach also allows transcriptome data to be compared directly with other microarray datasets that use genomic DNA as the common reference. However, two important caveats emerged in the course of the work: first, for several technical reasons the microarray data can be relatively compressed compared with results from more gene-specific analytical procedures such as quantitative RT-PCR or S1 nuclease protection; and second, M600, used as the "wild-type" strain here, turned out to have a duplication of the genes SCO0021-1004 compared with the M145 DNA used as the microarray reference DNA [7]. This duplication may have had some direct effects on levels of mRNA and proteins corresponding to the duplicated genes, or indirect effects on genes elsewhere in the genome, but it did not affect the validity of the analysis of the effects of *bldA *deletion described in this paper.

Although protein samples were taken at all the time-points shown in Fig 1, the labour-intensiveness of 2-D gel analysis led us to confine the proteomic experiments to duplicate samples for each of five time-points (samples 1, 2, 4, 6 and 8), and to use high resolution 2-D gel separation only in the two pI ranges into which most proteins fall (4.5 – 5.5 and 5.5 – 6.7). Among the proteins excluded by this approach were integral membrane proteins and extracellular proteins (both of which have been analysed separately: [8,9]), proteins of less than ca.12 kDa or more than ca.120 kDa, and those with a basic pI (including many ribosomal proteins).

#### Detection of 74 differentially transcribed genes

For the transcriptome analysis, 5,983 genes remained for comparison between the strains after filtering out genes deemed to be 'non-changing' because they had log_2 _expression levels between -0.667 and 0.667 in all 16 time points (eight in M600, eight in Δ*bldA*). Applying different statistical approaches (see Methods), 74 genes – ~1% of all genes – were differentially transcribed between the wild-type and Δ*bldA *mutant at the specific thresholds applied (Table 1 and see Additional File 1). Since previous work has focussed on genes whose expression is reduced in *bldA *mutants, an interesting finding was that the expression of 13 of the 74 genes was significantly increased in the mutant strain (p-value < 0.05 and/or probability of false prediction (pfp) < 0.1 for Welch t-test and/or Rank Product analysis (see Methods) respectively).

**Table 1:** Genes differentially expressed in the transcriptome data as a result of *bldA *mutation^1^

Gene^2^	Annotated function^3^	P-value^4^	Mean pfp value^5^	Up/down in bldA^6^	Potential reason for absence in proteomic data^7^
SCO0072	possible secreted protein	N/A	0.055*	down	Secreted
SCO0247	conserved hypothetical protein	3.29E-06	N/A	down	Predicted 1 transmembrane domain
SCO0248	hypothetical protein	1.76E-05	N/A	down	Predicted pI = 11.6
SCO0297	secreted protein	8.25E-05	0.033	down	Secreted
SCO0472	possible secreted protein	N/A	0.076*	down	Secreted
**SCO0499**	possible formyltransferase	9.76E-04	N/A	down	
SCO0759	hypothetical protein	0.000976	N/A	down	Predicted 1 transmembrane domain
SCO0760	probable methyltransferase	0.000796	0.083	down	Predicted pI = 10.9
SCO0762	protease inhibitor precursor	0.0262	0.012	down	Secreted
SCO0990	integral membrane protein	7.72E-03	N/A	down	Predicted 7 transmembrane domains
SCO0991	conserved hypothetical protein	0.00772	N/A	down	Predicted transmembrane domain
SCO0994	integral membrane protein	0.0317	N/A	down	Predicted 9 transmembrane domains
SCO0995	probable methyltransferase	9.76E-04	N/A	down	Predicted 2 transmembrane domains
SCO1089	unknown	N/A	0*	up	None
SCO1815	probable 3-oxacyl-(acyl-carrier-protein) reductase	N/A	0.084*	down	Predicted 2 transmembrane domains
SCO1845	possible low-affinity phosphate transport protein	N/A	0*	up	Predicted 10 transmembrane domains
SCO1968	probable secreted hydrolase	N/A	0*	down	Secreted
SCO2435	hypothetical protein	0.00589	N/A	down	None
SCO3088	hypothetical protein	0.000415	N/A	up	Predicted pI = 9.7, mwt = 8377 Da
**SCO3285**	large gly/ala rich protein	7.72E-03	N/A	up	
SCO3286	secreted protein	0.00772	0.05	up	Secreted
SCO3428	50S ribosomal protein	N/A	0.082*	down	Predicted pI = 10.8, mwt = 6416 Da
SCO3608	hypothetical protein	N/A	0.1*	up	Predicted 3 transmembrane domains
SCO3717	probable cation transport system component	N/A	0.068	up	Predicted 7 transmembrane domains
SCO3718	probable cation transport system component	N/A	0	up	Predicted 11 transmembrane domains
SCO4131	integral membrane protein	0.0389	N/A	down	Predicted 2 transmembrane domains
SCO4173	unknown	N/A	0.088*	down	Predicted 1 transmembrane domains
SCO4174	possible integral membrane protein	N/A	0.088*	down	Predicted 1 transmembrane domains; predicted pI = 11.0
SCO4187	putative membrane protein	0.0404	0.1	down	Predicted pI = 9.5, mwt = 6268 Da
SCO4246	hypothetical protein	5.89E-03	N/A	down	Predicted pI = 9.7
**SCO4252**	hypothetical protein	3.29E-06	0.017	down	
**SCO4253**	conserved hypothetical protein	5.93E-05	5.66E-03	down	
SCO4256	possible hydrolytic protein	0.00772	N/A	down	None
SCO4262	hypothetical protein	0.000976	0.083	down	Predicted pI = 11.7, mwt = 12884 Da
SCO4295	Cold shock protein scoF4	0.00126	0.053	down	Predicted mwt = 7387 Da
SCO4442	hypothetical protein	0.00608	0.09*	down	Predicted mwt = 6484 Da
SCO4653	50S ribosomal protein	N/A	0.097*	down	None
SCO4661	elongation factor G	N/A	0.088*	down	None
**SCO4677**	possible regulatory protein	N/A	0.038*	down	
SCO4703	50S ribosomal protein	N/A	0.076*	down	Predicted pI = 10.0
SCO4704	50S ribosomal protein	N/A	0.044*	down	Predicted pI = 10.6
SCO4705	50S ribosomal protein	N/A	0.087*	down	Predicted pI = 11.3
SCO4706	30S ribosomal protein	N/A	0.095*	down	Predicted pI = 10.8
SCO4707	50S ribosomal protein	N/A	0.091*	down	Predicted pI = 10.2
SCO4712	50S ribosomal protein	N/A	0.087*	down	Predicted pI = 10.2
SCO4713	50S ribosomal protein	N/A	0.076*	down	Predicted pI = 10.3
SCO4714	50S ribosomal protein	N/A	0.088*	down	Predicted pI = 9.6
SCO4716	30S ribosomal protein	N/A	0.077*	down	Predicted pI = 9.6
SCO4717	50S ribosomal protein	N/A	0.043*	down	Predicted pI = 9.8
SCO4718	50S ribosomal protein	N/A	0.074*	down	Predicted pI = 10.6
SCO4719	30S ribosomal protein	N/A	0.071*	down	Predicted pI = 10.1
**SCO4994**	hypothetical protein	0.0136	N/A	down	Predicted 1 transmembrane domain
SCO5013	secreted protein	0.0479	N/A	down	Secreted
**SCO5073**	possible oxidoreductase	4.79E-02	0*	down	
SCO5074	possible dehydratase	N/A	0*	down	Predicted pI = 8.6; secreted
**SCO5079**	conserved hypothetical protein	N/A	0*	down	
SCO5123	membrane protein	0.000415	N/A	down	Predicted pI = 8.2, mwt = 6622 Da
SCO5124	hypothetical protein	0.00512	N/A	down	Predicted pI = 7.12, mwt = 7054 Da
SCO5125	hypothetical protein	0.000788	N/A	down	None
SCO5166	putative helicase	5.12E-03	N/A	up	Predicted pI = 9.5
SCO5225	ribonucleotide-diphosphate reductase small chain	N/A	0.007*	up	Predicted 2 transmembrane domains
SCO5624	30S ribosomal protein S2	N/A	0.089*	down	None
SCO5649	unknown	N/A	0.1*	down	None
SCO6197	secreted protein	3.29E-06	0.063	down	Secreted
SCO6198	secreted protein	8.09E-03	N/A	down	Secreted
SCO6346	hypothetical protein	6.08E-03	N/A	down	None
SCO6362	Probable two-component sensor	0.00589	N/A	down	Predicted 6 transmembrane domains; pI = 10.43
**SCO6637**	hypothetical protein	0.000788	N/A	down	
**SCO6638**	hypothetical protein	0.000415	N/A	down	
SCO6808	possible ArsR-family regulator	0.00219	N/A	down	Predicted mwt = 13271 Da
SCO6958	putative membrane protein	0.036	N/A	up	Predicted 3 transmembrane domains; pI= 8.7; mwt = 11304 Da
SCO7510	peptidyl-prolyl cis-trans isomerase	N/A	0.01*	up	None
**SCO7511**	glyceraldehyde 3-phosphate dehydrogenase	N/A	0*	up	
SCO7657	secreted protein	1.76E-05	0.066*	down	Secreted

^1 ^presented in more detail in Additional File 8.

^2 ^genes in bold were also found to be different in the proteome analysis detailed in Additional File 5. Underlined genes were more highly expressed during all stages of growth in M600 relative to the *bldA *mutant. Q-RT-PCR was performed with the 16 RNA samples from replicate 3 time courses, using specific primers and probes covering 50–150 bp, for each of the following genes: SCO4295, SCO5013, SCO6808, and SCO7657; five genes up-regulated in the *bldA *mutant – SCO3088, SCO3285, SCO3286, SCO5166, SCO6958; the TTA-containing gene SCO6638; and the ribosomal protein genes SCO4648, SCO4702, SCO4705, SCO4717. See Additional File 2 for these results.

^3^from ScoDB [55]

^4 ^from a Welch t-test applying the Benjamini and Hochberg multiple testing correction

^5 ^average pfp (Probability of False Prediction) values from time-point comparisons outputted from Rank Product Analysis. * denotes those genes found differentially expressed at one time point only.

^6^determined from a visual inspection of expression profiles in Additional File 1

^7 ^pI and Mwt values from ScoDB [55]: transmembrane domain predictions from TmPred [56]

In order to validate the differentially expressed gene list independently, 14 of the 74 genes were also analysed by quantitative real time PCR (Q-RT-PCR), including SCO6638, one of two TTA-containing genes in the list of 74 genes (see Table 1 for list). The expression profiles generated by the two techniques were broadly in agreement with each other, exhibiting Spearman correlation coefficients ranging from 0.54 to 0.91 (see Additional File 2).

#### Unexpected effects of *bldA *deletion at growth stages preceding stationary phase, leading to the discovery of abnormal ppGpp levels in the *bldA *mutant

**Figure 2:**
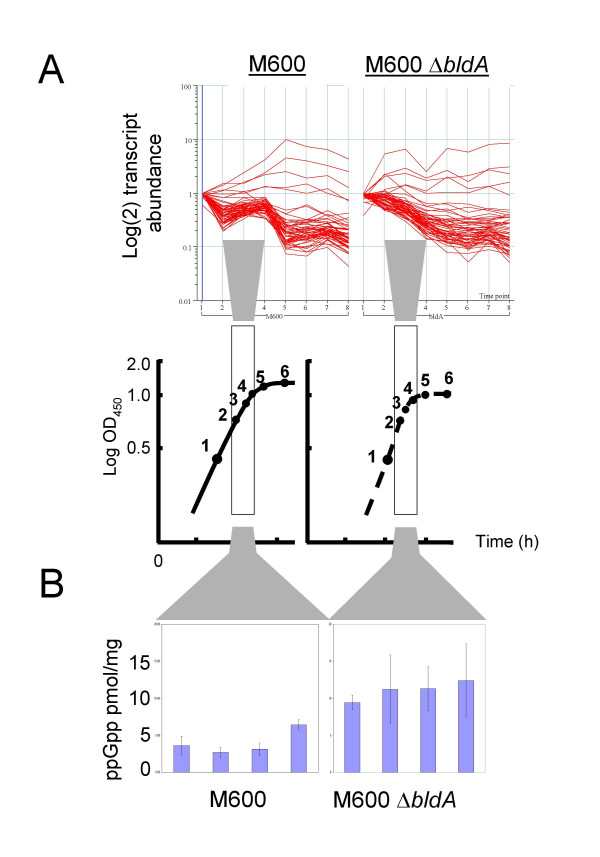
Aberrant transition phase physiology as a result of *bldA *mutation. (A) Patterns of ribosomal protein gene expression in M600 and M600 Δ*bldA *are markedly different at timepoints 3 and 4. (B) Synthesis of the stringent factor ppGpp is increased in transition phase cultures of M600 Δ*bldA*.

Since *bldA *mutants grow well, but differ from the wild-type most obviously in attributes expressed after the main growth period (aerial growth and secondary metabolism [10]), we expected to find effects of *bldA *deletion mainly on stationary phase gene expression. While one-third of the 74 differentially transcribed genes conformed to this expectation, the rest did not (see Additional File 1): two genes were most highly expressed during exponential growth in the mutant strain (SCO3088 and the putative RNA helicase gene SCO5166), 20 were consistently more highly expressed during all phases of growth and a further 16, encoding elements of the translational apparatus, showed a transient upshift at transition phase in the wild-type but not in the mutant (Table 1, see Additional File 1 and illustrated in Fig. 2A). The tight transition phase-dependent expression change of the latter genes has also been seen in other wild-type strains (V. Mersinias, G.B. and C.P.S., unpublished data). These observations suggest that, in addition to its important role in stationary phase biology, *bldA *may also influence transcription of certain genes at earlier stages, either due to an involvement of *bldA *in at least some exponential and transition phase processes, or because of some indirect effect of the loss of *bldA *on cellular physiology. Such indirect effects could include a 'shadow' cast by effects of *bldA *on the formation of the spores used to inoculate the cultures (inoculum preparation is known to influence the behaviour of cultures in industrial fermentations), or some kind of stress response caused by the occupation of a proportion of ribosomes by untranslatable UUA codon-containing RNA (see below). For example, two of these genes (SCO4262 and SCO6638) contain TTA codons, and translation of the transcripts detected is therefore dependent on *bldA*. The identification of these genes as being significantly differently expressed suggests that their transcript abundance, in addition to their translation, is in some way *bldA*-dependent. The 15 differentially expressed ribosomal protein genes listed in Table 1 mostly fall within one cluster (SCO4701-SCO4721). Further members of this and other ribosomal protein gene clusters appeared in the top rankings of genes down-regulated in *bldA *compared to M600 but did not meet the statistical threshold criteria applied here. Similar extensive alterations in patterns of protein abundance were not observed in the proteome study, primarily because most of the ribosomal proteins have isoelectric points that lie outside those used in the analysis (see Additional File 3); but in any event the observed modulations in transcription may not have resulted in detectable changes in abundance of stable proteins such as those making up ribosomes. However, comparable changes have been documented at the proteome level in pulse-chase experiments, using a different medium, by Vohradsky and Thompson [11].

The effects on transition-phase expression of ribosomal protein-encoding genes raised the possibility that the changes might be mediated via ppGpp, the stringent factor that controls transcription of rRNA and certain ribosomal protein operons in *E. coli *and many other bacteria, including streptomycetes [6]. Previous studies had shown that ppGpp concentrations increase transiently during transition phase growth of *S. coelicolor *in SMM medium [12]. Since ppGpp is synthesised when an insufficient supply of aminoacyl-charged tRNAs causes translation to stall, it was possible that stalling of ribosomes encountering a UUA codon during translation in a *bldA *mutant could similarly stimulate ppGpp synthesis. Intracellular ppGpp concentrations were therefore analysed by HPLC at four comparable timepoints leading up to transition phase in cultures of M600 and M600 Δ*bldA *similar to those used for the RNA and protein analysis. The levels in the *bldA *mutant were consistently two- to six-fold higher at each timepoint in the mutant than in the parent strain (Fig. 2B), supporting the idea that the observed large-scale changes in transition-phase transcription patterns are related to altered patterns of ppGpp synthesis. GTP levels were comparable between the two strains.

These results raise the strong possibility that some of the changes in the mRNA and protein profiles resulting from the deletion of *bldA *may be indirect effects of changes in the level of ppGpp. However, such effects are unlikely to be the principal reason for the most obvious features of the mutant phenotype, since the deficiencies in morphological development and actinorhodin synthesis can be suppressed by specifically replacing TTA codons in appropriate regulatory genes by alternative leucine codons [13,14].

#### Among 102 differentially represented protein spots (corresponding to 84 genes), the majority are maximally abundant in stationary phase in the wild-type

**Figure 3:**
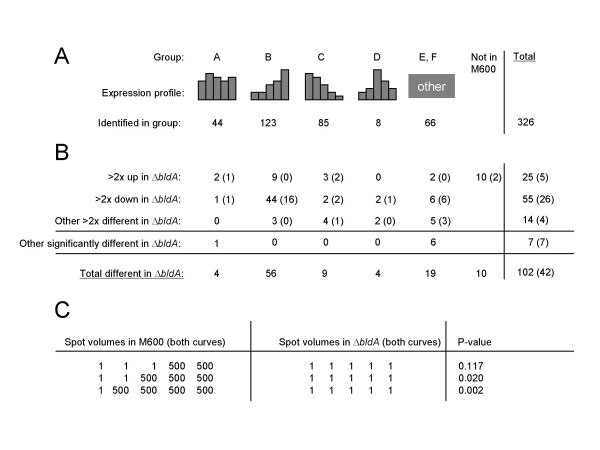
Summary of results from analysis of the proteome. The majority of spots that are different in the Δ*bldA *mutant are most abundant in M600 during the stationary phase. (A) Grouping of identified protein spots according to their abundance profiles in M600. (B) Protein spots identified that are considered to be altered in expression in Δ*bldA*. Figures given are the number of spots that reproducibly exhibit a two-fold or higher change in abundance in the Δ*bldA *mutant strain, followed by a bracketed figure indicating the number of these that are also statistically significantly different. (C) Table showing that the statistical approach is limited to proteins that are reproducibly more abundant in at least three of the five timepoints.

In the global proteomic analysis, about 750 spots were subjected to fluorescence quantification in each pI range, i.e. about 1,500 in all. Of these, 336 were identified by mass spectrometry of tryptic digests (see Additional File 4 for quantitative data for these identified spots). The selection of spots for characterisation was to some extent guided by obvious temporal regulation, or changes in abundance between the two strains. 42 of the 336 spots were differentially represented between the two strains according to the Mann-Whitney U-test, and 35 of these were also among the 95 found to be differentially represented by a different procedure, employed to compensate for a limitation of the statistical approach (discussed below). This second procedure searched for spots whose normalized volumes were changed two-fold or more between strains in both biological duplicate growth curves and in at least two (of the five) corresponding timepoints (see Methods for further details of the analytical procedures). The two groups of proteins gave a combined total of 102 differentially represented spots (about 7% of the c. 1500 quantified spots: see Additional File 5, and Fig. 3). As in the transcriptome analysis, some *bldA*-influenced protein spots (25/102) were more abundant in Δ*bldA *than the parent strain, one of these (SCO3285, encoding a large glycine/alanine-rich protein) being detected in both analyses.

To assess the impact of *bldA *mutation on proteins expressed at different growth phases in the parent strain, the 336 proteins were grouped into one of six temporal expression pattern classes by applying a set of simple mathematical rules (see Methods). A summary is shown in Fig. 3A (see Additional File 6 for detailed lists). We emphasise that the bias in spot selection means that some of these classes are over-represented (notably class B, expression mainly late) or under-represented (such as class A, expression uniform throughout growth). As indicated in Fig. 3, the great majority of the spots influenced by *bldA *showed strong up-regulation in stationary phase in the wild-type (class B), though four examples were found of a *bldA*-influenced spot that was more or less constitutive, echoing the evidence from microarray analysis that *bldA *has some effects during exponential phase (class A). The strong growth-stage-specificity of most proteins influenced by *bldA *was consistent with the observation that the *bldA-*specified tRNA is unusual in being most abundant in stationary phase, in contrast to most tRNAs, which are most abundant at high growth rates [15,16]. Most of the differences between protein spot lists from the two mathematical approaches can be accounted for by the blindness of the Mann-Whitney U-test to the expression pattern most common among *bldA*-influenced spots. Figure 3C shows that when a protein spot is completely absent at all timepoints in both growth curves of one strain, it needs to be present in at least 3 of the 5 timepoints in both curves of the second strain in order to be called significantly different by that procedure. Thus, the largest discrepancies between the two procedures are in expression group B, where protein spots may be detected only in the last two timepoints, and in the group of 10 proteins not detected at all in M600 (Fig. 3B). In total these groups account for 48 of the 59 differences between the two protein lists, and indicate why it was important to use both differential representation assessment procedures. The reasons for the unexpectedly small overlap between the differential expression lists obtained by proteome and transcriptome analyses are discussed below.

#### *bldA *mutation affects some proteins post-translationally

An unexpected new aspect of the *bldA *phenotype was the changes observed in post-translational modification/processing of 11 proteins (Table 2 and see Additional File 7). Nine of these were detected as pairs of spots with similar apparent molecular weights but differing isoelectric points, and in three of the nine cases one of the two forms of the protein was completely absent in the *bldA *mutant (SCO1916, SCO3073, and SCO3137). The other two of the 11 proteins were detected as pairs of spots differing in both molecular weight and isoelectric point (SCO5465, SCO2271). One of the SCO5465 spots in M600, with an apparent molecular weight of about 40 kDa, was absent from the *bldA *mutant, while the other, corresponding to the 23 kDa predicted from its amino acid sequence, and presumably unmodified, was more abundant in the mutant. Neither of the two SCO2271 spots coincided with the 2D gel location predicted for a 48 kDa protein of pK 6.8. However, SCO2271 is expected to be processed and exported from the cell, and the observed position of the higher molecular weight spot coincides with that predicted for the secreted protein (pH 5.9, 45 kDa). This form of the protein, possibly anchored in the cell membrane by a predicted transmembrane helix at its C-terminus, was more abundant in the parent strain in the stationary phase samples while the smaller of the two protein spots was approximately equally abundant in both strains.

**Table 2:** Changes observed in the post-translational modification of proteins as a result of *bldA *mutation

Gene	Function^1^	Alteration in protein abundance^2 ^observed for:	
		Spot 1	Spot 2
A) Spots differ in isoelectric point (pI): spot 1 has more acidic pI than spot 2			
SCO1246	BioD (dethiobiotin synthetase)	>2-fold up in Δ*bldA*	>2-fold down in Δ*bldA*
SCO1916	Putative transferase	Absent in Δ*bldA*	No change
SCO2390	FabF (3-oxoacyl- [ACP] synthase II	No change	>2-fold down in Δ*bldA*
SCO2618	ClpP2 (Clp-protease subunit 2)	>2-fold up in Δ*bldA*	>2-fold down in Δ*bldA*
SCO3073	HutU (urocanate hydratase)	No change	Absent in Δ*bldA*
SCO3137	GalE1 (UDP-glucose 4-epimerase)	Absent in Δ*bldA*	No change
SCO3409	Ppa (inorganic pyrophosphatase)	>2-fold up in Δ*bldA*	>2-fold down in Δ*bldA*
SCO4164	CysA (thiosulphate supfurtransferase)	>2-fold up in Δ*bldA*	>2-fold down in Δ*bldA*
SCO7400	ABC transport protein	>2-fold down in Δ*bldA*	No change
			
B) Spots differ in Mwt (and pI): spot 1 has higher Mwt than spot 2			
SCO2271	Putative membrane protein	>2-fold down in Δ*bldA*	No change
SCO5465	Conserved hypothetical protein	Absent in Δ*bldA*	>2-fold up in Δ*bldA*

^1^from ScoDB [55]

^2^protein abundance profiles for all 22 protein spots are detailed in Additional File 7.

#### Summary of global effects of *bldA *mutation

In summary, 147 genes were identified as being affected by *bldA *deletion, 63 and 73 uniquely from transcriptomics or proteomics respectively (Table 1 and see Additional Files 5 and 8). Eleven genes were found in both analyses (their transcript and protein abundance profiles are compared in Additional File 9). All 147 genes are considered together in subsequent sections dealing with the effects of *bldA *deletion on secondary metabolism and with the genetic routes by which some genes are affected by the deletion. We also consider some evidently meaningful effects of the *bldA *deletion that were not found by the statistical analysis.

### *Mutation of bldA has little effect on primary metabolic genes associated with growth, but does affect some genes associated with nutritional stress, as well as more secondary metabolic genes than previously recognised.*

#### Primary metabolism

The growth rate of the *bldA *mutant in minimal media is as rapid as that of the wild-type (possibly it is even faster: Fig 1; D. W. Kim and K. J. Lee, personal communication). The mutant was therefore not expected to show significant changes in the abundance of mRNAs or proteins involved in primary metabolism. In general confirmation of this, the major biosynthetic pathways for amino acids, nucleotides and vitamins were apparently unaffected, with the exceptions of aromatic amino acid biosynthesis (SCO1496, chorismate synthase; and SCO2115, one of two *aroH*-like genes for the first step) and biotin biosynthesis (SCO1244, 1246); and among the central pathways of primary carbon metabolism (glycolysis, the pentose phosphate pathway, the citric acid cycle, the glyoxylate cycle, gluconeogenesis), the only major changes seen involved *increased *abundance in the *bldA *mutant of protein and mRNA from one of three genes annotated as glyceraldehyde-3-phosphate dehydrogenase (SCO7511, *gap2*) (of the two other paralogues, SCO1947 protein was detected in all samples and SCO7040 was not detected in any). SCO7511 is orthologous with a *gap *gene of *Streptomyces aureofaciens *that was reported to be induced at the time of aerial growth on glucose-free medium [17]. In addition, a spot comprising a fragment of citrate synthase (SCO2784) was enhanced in the proteome of the mutant, though the major citrate synthase spot was unaffected.

However, some effects were seen on more peripheral aspects of primary and salvage metabolism, such as might be expected to be active after the main growth phase. The more basic of two SCO3073 (putative urocanate hydratase, *hutU*) spots was absent from the mutant; the product of a conserved hypothetical gene apparently translationally coupled to the downstream *rocD *(ornithine aminotransferase)-like gene was less abundant in the mutant; and a putative uracil phosphoribosyltransferase (SCO4041, nucleotide salvage) was likewise less abundant in the mutant. Changes in the abundance of both a basic and an acidic form of SCO4164, a homologue of thiosulphate sulphurtransferase (*cysA*, SCO3920), were seen in the mutant.

Interestingly, multiple protein forms were associated with ten of the 17 *bldA*-influenced protein spots potentially associated with primary/intermediary metabolic processes (central carbon metabolism, amino acid biosynthesis etc), suggesting that the majority of these effects may be indirect subtle changes in post-translational modifications (see Additional File 5).

#### Secondary metabolism

**Figure 4:**
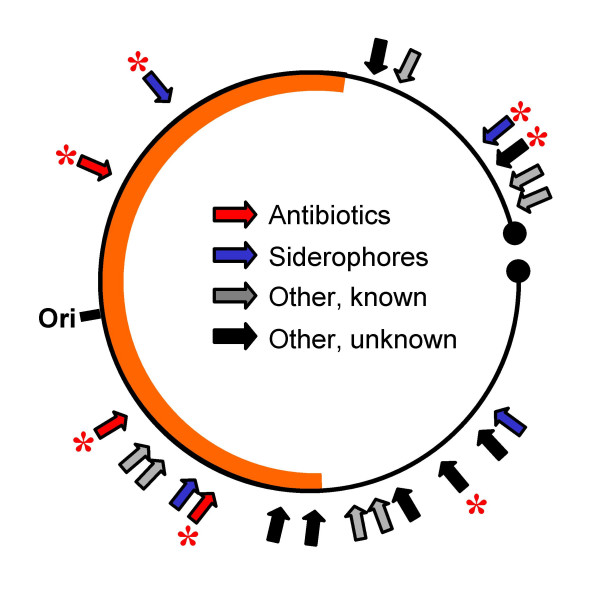
Mutation in *bldA *has wide-ranging effects on secondary metabolism. The figure presents a stylised illustration of the 8.7 Mbp *S. coelicolor *linear chromosome, with the 21 genes or gene clusters that determine secondary metabolite production marked with arrows, and those that are altered in expression in M600 Δ*bldA *asterisked. The thick orange line represents the central core of the chromosome rich in essential functions and containing the origin of replication (ori).

Streptomycetes are very important producers of antibiotics and other valuable secondary metabolites. Bentley *et al*. [4] identified 21 genes or gene clusters in the genome sequence of *S. coelicolor *that are likely to determine secondary metabolite production. In our proteomic analysis, the abundance of gene products from seven of these was found to be affected by *bldA*, one of them, a Type III polyketide synthase (SCO7221), being more abundant in the mutant (Fig. 4 and see Additional File 10). Previously, only two of these gene sets (*act *and *red*) had been found to be influenced by *bldA*. In general, proteomic analysis here and in previous work [18] failed to detect some of the proteins specified by each cluster. In most cases this reflected the properties of the proteins themselves (size, charge, cellular location). For reasons discussed below, only two of the secondary metabolism gene sets (for Act and coelichelin) were among those identified as being affected by *bldA *in the transcriptome analysis (see Table 1).

##### *act *gene cluster (SCO5070-5092)

The acetate-derived blue aromatic polyketide, actinorhodin, whose production is encoded by the extensively studied *act *genes, is absent from *bldA *mutants. Each of the 5 *act *biosynthetic transcription units was represented by at least one spot in the proteomic analysis, and each showed strong dependence on growth phase and on *bldA *(see Additional File 10). Three *act *genes, SCO5073, SCO5074 and SCO5079, were also identified from the transcriptome analysis. Putting these data together with previous less extensive results, it appears that every *act *biosynthetic operon is likely to be regulated by *actII-ORF4*, the pathway-specific regulatory gene containing the TTA codon previously shown to mediate *bldA*-dependence of actinorhodin production [14]. Most *act *genes were not identified as significantly differentially expressed by transcriptome analysis, although there is compelling evidence from previous studies that they are very markedly *bldA*-dependent [14]. Examination of the microarray data revealed excessive variance within the data for these genes (itself probably a consequence of noticeable variation in the timing and onset of pigment development between replicate experiments). This may be why they failed to meet the stringent statistical criteria employed: relaxing the criteria yielded more *act *genes (data not shown; with Rank Product analysis the additional *act *genes had associated p-values of less than 0.05 but the pfp values were above 0.1).

##### *red *gene cluster (SCO5877-5898)

The red prodiginine pigments determined by the *red *gene cluster are absent from *bldA *mutants except under certain conditions (e.g. low phosphate [19]). Only one Red biosynthetic protein, the methyl transferase RedI, was detected in this study. It appeared in the later time points, when *redI *transcripts also became more abundant. RedI abundance was strongly dependent on *bldA*, probably because of a TTA codon in *redZ *[19], which encodes an atypical response regulator believed to activate a second regulatory gene, *redD*, that in turn activates at least some of the *red *biosynthetic genes [20]. Thus, the *bldA*-dependence of RedI may reflect either a direct dependence of *redI *on RedZ, or an indirect effect via RedD. As with the *act *genes (see above), further *red *genes were detected as differentially transcribed only when the statistical criteria were relaxed.

##### *cda *gene cluster (SCO3210-3249)

*S. coelicolor *produces a lipopeptide antibiotic CDA which is chemically similar to daptomycin, a drug recently approved for the treatment of certain MRSA infections [21]. The oxygenase encoded by SCO3236, the only protein detected from the CDA cluster, dramatically increased in abundance in the parental strain during transition phase before falling back during late stationary phase. Levels of this protein were reduced in the *bldA *mutant.

##### Deoxysugar/glycosyltransferase cluster (SCO0381-0401)

These 21 genes are arranged in what could be a single operon, with the largest intergenic region being 102 bp between SCO0386-0387. They include two TTA-containing genes (SCO0383 and 0399), neither of regulatory character, and neither being represented in the proteome. Proteomics data were collected for five proteins from this cluster, and four were found to be reduced in abundance by two-fold or more in the *bldA *mutant. The complete absence from the *bldA *mutant of gene products from the last two genes in the cluster, SCO0400 and 0401, suggests that the TTA codons in upstream genes could exert polarity effects. The reduced abundance of mRNA for the first two genes in the cluster, which are upstream of the TTA codons, although not statistically significant, perhaps suggests that polarity effects on protein synthesis may result in destabilisation of the whole message (see also below).

##### Type III PKS (SCO7221)

The Type III polyketide synthase specified by SCO7221, which has very recently been shown to be responsible for the production of a germination inhibitor, germicidin [22], increased slightly in abundance during growth of the parent strain M600, peaking in the last stationary phase sample, but was 5- to 10-fold overproduced in the *bldA *mutant at all time-points (see Additional File 10). QRT-PCR confirmed that the transcript was also more abundant in the mutant strain, especially in the later timepoints (data not shown).

##### Coelichelin biosynthetic cluster (SCO0489-0499)

The non-ribosomal peptide synthetase encoded by this cluster produces an iron-chelating metabolite, coelichelin [23]. Although there are no TTA-containing genes in or near the cluster, the products of SCO0498 and 0499 were less abundant in the *bldA *mutant, while the products of SCO0490 and 0494 were unaffected. The SCO0499 gene was also identified as being significantly differentially expressed from the transcriptome data. Unusually among the secondary metabolism-related proteins detected in this work, these gene products were readily detected before the onset of stationary phase (see Additional File 10). The presence of regulatory DNA sequence motifs similar to iron-dependent repressor binding sites upstream of several genes in this cluster suggests responsiveness to the intracellular iron concentration [24]. It is not known whether the effects of *bldA *are independent of iron-responsiveness.

###### Desferrioxamines biosynthetic cluster (SCO2782-2785)

Like the coelichelin biosynthetic cluster above, the desferrioxamines cluster encodes production of an iron siderophore [25], and possesses an iron-dependent repressor motif upstream of the first gene in the cluster (SCO2782). Two proteins from this cluster were detected in the proteomics data from this experiment: SCO2784 protein was more abundant in the *bldA *mutant during some of the early timepoints when compared to the parental strain, and SCO2785 shows a slightly altered pattern of expression in the mutant strain.

### *Roles of regulatory and TTA-containing genes in the changed patterns of gene expression in the *bldA *mutant*.

Of the 147 TTA-containing genes in the annotated genome of *S. coelicolor*, 19 are predicted to encode regulatory proteins [26]. None of these 19 proteins was detected by proteomics, presumably because of low abundance in the growth conditions studied. However, reduced expression of genes known to be activated by TTA-containing regulatory genes provided indirect evidence for the absence from the *bldA *mutant of the regulatory proteins encoded by *adpA *(which controls SCO0762 [8]), *actII-ORF4 *(which controls the *act *cluster genes [27,28]), *redZ *(which controls the *red *cluster genes via *redD *[20]) and SCO4263 (which controls SCO4251-4253: see below). In addition, regulatory proteins encoded by three TTA-free genes were reduced in abundance in the *bldA *mutant, perhaps accounting for at least some of the changes in gene expression in the mutant. One of the three is an ArsR-family transcriptional regulator (SCO6808), so it was interesting that a motif reminiscent of the ArsR-binding site determined in *E. coli *[29] (see Additional File 11, motif 3) precedes a group of six differentially expressed genes. Another of the three is a nickel-responsive Fur-like repressor of a putative nickel transport operon (SCO4180 [30]). A SCO4180 deletion mutant exhibited a higher intracellular nickel concentration than the parent [30], so *bldA *may be similarly affected. The third (SCO4677) showed homology to sensory histidine protein kinases. SCO6362, encoding another such kinase, was also significantly altered in its pattern of transcription.

#### One of many likely operons affected by *bldA *is the target of a regulator encoded by a nearby TTA-containing gene

**Figure 5:**
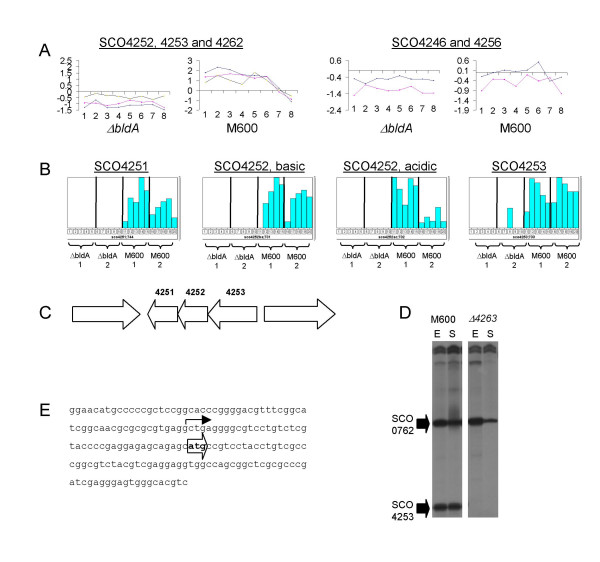
Expression of a cluster of function-unknown genes is dependent on *bldA*. (A) Transcriptome and (B) proteome data showing the *bldA*-dependence of expression of the genes of unknown function SCO4251-4253, SCO4246, SCO4256 and SCO4262. (C) Genetic organisation of the SCO4251-4253 locus. (D) Transcription of the SCO4253 gene during growth of strains M600 and M600 Δ SCO4263 in SMM. RNA was isolated during the mid-exponential (E) and early stationary (S) phases of growth and subjected to S1 nuclease protection analysis using uniquely end-labelled PCR-generated probes. Transcription of the SCO0762 gene was analysed as an internal control for RNA quality and loading. (E) Sequence upstream of the SCO4253 gene indicating the location of the single transcription start site (bent arrow), upstream of the ATG translation start codon (block arrow).

Of the 147 genes identified as being *bldA*-influenced in Table 1 or Additional File 5, 87 are probably co-transcribed with other genes [31] in a total of 59 putative operons. Sixteen of these operons have more than one member listed in Table 1 or Additional File 5 (equivalent to 42 genes), reinforcing their classification as *bldA*-dependent. Apart from those involved in secondary metabolism (see above), the others include a cluster of function-unknown genes whose expression was particularly strongly dependent on *bldA *at the mRNA (SCO4246, SCO4252-3, SCO4256 and SCO4262; Fig. 5A) and protein levels (SCO4251-3; Fig.5B) [9]. A search of the genome revealed a nearby TTA-containing gene, SCO4263, encoding a LuxR-family regulator. The effect of deleting SCO4263 on expression of the SCO4251-4253 operon was therefore analysed. S1 nuclease protection experiments indicated that transcription of SCO4253, the first gene in the putative SCO4251-4253 operon (Fig. 5C), starts 32 bp upstream of the translational start site, and is completely dependent on SCO4263 (Fig. 5D, E). As a control, transcription of another strongly *bldA-*dependent gene, SCO0762 [8], was unaffected in the SCO4263 mutant. Proteomic analysis also demonstrated that the SCO4251-4253 proteins, readily detected in the parent strain, were absent from the SCO4263 mutant (data not shown), confirming the *bldA*-dependence of these three genes via the TTA-containing regulator. There was no obvious phenotypic change associated with the deletion of SCO4263.

#### Apparent destabilisation of UUA-containing polycistronic mRNA by *bldA *deletion

The transcript of SCO6638, a TTA-containing gene of unknown function, was less abundant, and its gene product – the only one from a TTA-containing gene to be detected in the proteome analysis – completely absent, in the *bldA *mutant. The stop codon of SCO6638 overlaps the start site for the downstream SCO6637 gene, whose RNA and protein products were also less abundant in the *bldA *mutant, indicating that the two genes are cotranscribed and possibly translationally coupled. Possibly, a polarity effect on protein synthesis resulting from stalling of translation at the SCO6638 UUA codon upstream accounts for reduced SCO6637 expression, and the consequent uncoupling of transcription and translation may further result in exposure of the mRNA to RNase action, in turn reducing the half-life and overall abundance of mRNA corresponding to both genes. Such an explanation may also account for the similar situation described above for the deoxysugar cluster (SCO0381-0401).

Another group of linked genes, SCO0991-0995, most of which encode integral membrane proteins, was preferentially transcribed late in the wild-type, and transcription of SCO0991 (protein kinase), SCO0994 (function unknown) and SCO0995 (methyl transferase) was reduced in the *bldA *mutant. SCO0992, the TTA-containing gene encoding a putative cysteine synthase, and SCO0993 were not identified as being significantly affected by *bldA *mutation, presumably failing to meet the statistical criteria (though there are alternative explanations, including differences in half-lives of component transcripts or the presence of internal independent promoters, which are predicted to be common in *Streptomyces*: e.g. Laing *et al*. [32]). The half-life of the UUA-containing putative polycistronic SCO0991-0995 mRNA may therefore also be affected by *bldA-*dependent uncoupling of transcription and translation. We note that the gene next to, and diverging from, the SCO0991-0995 cluster, SCO0990, and also encoding a membrane protein, was also *bldA*-dependent.

## Discussion

### Few of the genes found to be affected by *bldA *contain TTA codons

Some 147 genes were found by RNA and/or proteome analysis to be affected by *bldA*. Further differences are likely to be found when the strains are growing on the surface of agar, conditions in which the mutant forms no obvious aerial mycelium or spores. Some genes (31/147) showed increased expression in the *bldA *mutant. Of the 47 whose protein products were found to be less abundant in the mutant, only one (SCO6638) has a TTA codon. [Only one other protein among those seen on gels was specified by a gene (SCO6717) annotated as TTA-containing, and closer examination of the SCO6717 sequence indicates that the TTA codon falls upstream of a likely translation start codon.] The gels examined contained about 1500 different protein spots. Based on a reported average of 1.2 spots per gene [18], these correspond to about 1250 genes, i.e. about 16% of the genome. The products of the TTA-containing genes in the *S. coelicolor *genome (147 in the analysis of Li *et al*. [26]) are therefore strikingly under-represented in our analysis. Thus, the growth conditions used here (submerged growth in casaminoacids-supplemented minimal medium) did not favour the expression of most of these genes. This would be consistent with the idea that many TTA-containing genes are adapted for expression either during surface growth and differentiation, or in specialised ecological niches or physiological responses. A similar conclusion was reached by Li *et al. *[26], in a study in which mutations in 21 TTA-containing genes of *S. coelicolor *were found to cause no obvious phenotypic changes. Since nearly all of the genes that seemed to be influenced by *bldA *in our analysis lack a TTA, these effects must have been indirect. Three indirect routes probably account for most of the effects (Table 3). First, some of the *bldA-*influenced genes are regulated by TTA-containing genes, most of which are expressed at levels too low to make them amenable to the analytical procedures employed. We estimate that dependence on such weakly-expressed TTA-containing genes accounts for the *bldA*-dependence of at least 15 genes found in the transcriptome and proteome analyses. The upstream sequences of the genes with differentially expressed transcripts were analysed to identify over-represented DNA motifs that may be indicative of co-regulation of genes by the same regulatory protein, and the ten statistically most significant motifs are shown in Additional File 11. None resemble binding motifs reported or proposed for the *S. coelicolor *regulators encoded by TTA-containing genes i.e. AdpA [8], ActII-ORF4 [33], RedZ (White and Bibb, personal communication), or previously reported consensus promoter sequences in *S. coelicolor*. Secondly, eight of the genes affected are probably co-transcribed with TTA-containing genes, and the resulting UUA-containing mRNAs may have a reduced half-life under conditions in which the UUA codon is not readily translated. Thirdly, *bldA *deletion changes the profile of ppGpp abundance and eliminates a transient upshift in transcription of ribosomal protein genes at the transition phase that precedes entry into stationary phase (see Fig. 2A). This extraordinary effect may arise through a triggering of the stringent response, because of ribosomes encountering untranslatable UUA codons in a small proportion of mRNAs. It is likely that the changed ppGpp levels will affect the expression of some genes even before transition phase, and that the disturbance of transition phase gene expression will have knock-on effects on gene expression during stationary phase, the stage at which most of the known phenotypic consequences of *bldA *mutation are manifested.

**Table 3:** *bldA*-dependent genes for which some direct link to *bldA *can be proposed

Gene^1^	Gene product^2^	Reference
*A: Containing a TTA codon*		
SCO4262	Hypothetical protein	[55]
SCO6638	Hypothetical protein	[55]

*B: Regulated by a TTA-containing gene*		
By *adpA*		
SCO0762	Protease inhibitor	[55]
By *SCO4263*		
SCO4246	Hypothetical protein	[55]
SCO4251	Putative secreted protein	This study
SCO4252	Hypothetical protein	This study
SCO4253	Conserved hypothetical protein	This study
By *act *II-ORF4		
SCO5071	Hydroxylacyl-CoA dehydrogenase, ActVI-ORFA	[14]
SCO5073	Putative oxidoreductase, ActVI-ORF2	[14]
SCO5074	Putative dehydratase, ActVI-ORF3	[14]
SCO5075	Putative oxidoreductase, ActVI-ORF4	[14]
SCO5078	Hypothetical protein	[14]
SCO5079	Conserved hypothetical protein	[14]
SCO5086	Ketoacyl reductase, ActIII	[14]
SCO5088	Polyketide beta-ketoacyl synthase, ActI-ORF2	[14]
SCO5090	Actinorhodin polyketide cyclase/dehdratase, ActVII	[14]
By *redZ*		
SCO5895	Putative methyltransferase, RedI	[20]

*C: In a putative operon with a TTA-containing gene*		
SCO0392	Putative methyltransferase	[31]
SCO0395	Putative epimerase/dehydratase	[31]
SCO0400	Putative epimerase	[31]
SCO0401	Putative aminotransferase	[31]
SCO0991	Conserved hypothetical protein	[31]
SCO0994	Integral membrane protein	[31]
SCO0995	Probable methyltransferase	[31]
SCO6637	Hypothetical protein	[31]

^1^the combined list of 147 *bldA*-affected genes as determined by transcriptomics and/or proteomics was considered

^2^from SCODB [55]

### Comparison of the results from proteomic and transcriptomic analysis ("dualomics")

**Figure 6:**
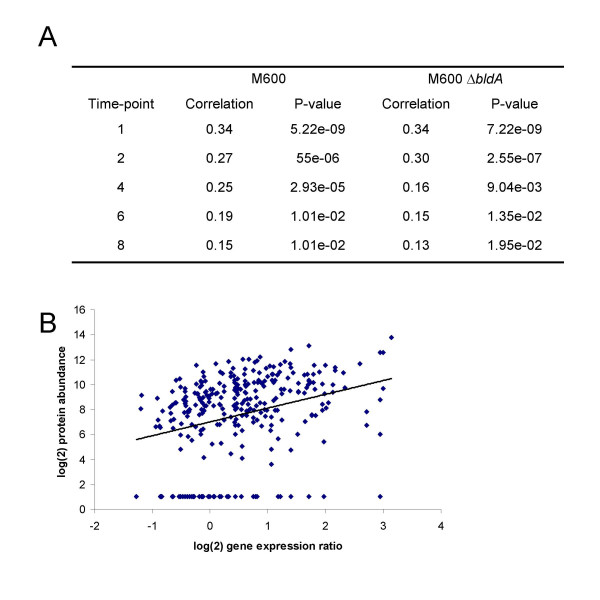
Significant global correlation between protein and transcript abundance. (A) Table listing correlation coefficients and significance P-values characterising the relationship between protein and transcript abundance in liquid-grown cultures of M600 and M600 Δ*bldA *at different stages of growth. (B) Graph illustrating the positive correlation between protein and transcript abundance in strain M600 at the earliest timepoint.

For a number of reasons, discussed at various points in the preceding text, we did not expect to find simple global correlations between transcript and protein levels, particularly in view of the fact that no system of statistical analysis is well-suited to integration of the kinds of data given here. Nevertheless, using the Spearman Rank correlation as detailed in the Methods, there was a weak but significant global correlation at the 5% level between the proteome and transcriptome values at each time-point, with correlation coefficients ranging from 0.15 to 0.34 for wild-type timepoints 8 and 1, respectively, and 0.13 to 0.34 for the corresponding times in Δ*bldA *(Fig. 6). Only 11 genes were positively correlated across both strains at the 0.05 probability level, while five genes notably even showed significant negative correlations between transcript and protein abundance (Table 4). Three of the latter genes encode highly important enzymes of primary metabolism (SCO1947, glyceraldehyde-3-phosphate dehydrogenase; SCO2198, glutamine synthetase; and SCO2736, citrate synthase), and in each case the anomaly could be ascribed to changes in the modification/processing state of the protein. A similar explanation has not been eliminated for the remaining two cases (SCO2634, SCO5466, neither of known function).

**Table 4:** Genes that have a high confidence in congruence between protein and transcript profile during growth

Gene/spot	Confidence ((1-p) × 100)	Spearman rank correlation	Annotated gene function
**Positive correlation in set A**			
SCO0379:123	100	1	catalase KatA
SCO1651	100	1	conserved hypothetical protein
SCO1776	100	1	putative CTP synthetase PyrG
SCO2554	100	1	DnaJ
SCO7510	100	1	peptidyl-prolyl cis-trans isomerase CypH
			
**Positive correlation in set B**			
SCO1212	100	1	putative ligase
SCO2115	100	1	putative 2-dehydro-3-deoxyphosphoheptonate aldolase AroH
SCO2539	100	1	Era-like GTP-binding protein
SCO3801	100	1	putative aminopeptidase
SCO3907	100	1	single strand DNA-binding protein ssb
SCO4809	100	1	succinyl CoA synthetase alpha chain SucD
SCO4813	97.7	0.87	phosphoribosylglycinamide formyltransferase PurN
SCO4824	98.4	0.89	bifunctional protein (methylenetetrahydrofolate dehydrogenase and methenyltetrahydrofolate cyclohydrolase FolD
SCO4921	100	1	putative acyl-CoA carboxylase complex A subunit AccA2
SCO5745	100	1	conserved hypothetical protein
			
**Positive correlation in set C**			
SCO0379:123	97	0.85	catalase KatA
SCO1244	98	0.88	Biotin synthase BioB
SCO1489	98.4	0.89	DNA-binding protein BldD
SCO1523	98.4	0.92	conserved hypothetical protein
SCO1651	98.4	0.89	conserved hypothetical protein
SCO1776	99.8	0.96	putative CTP synthetase PyrG
SCO2949	98	0.88	UDP-N-acetylglucosamine transferase MurA
SCO3122	98.4	0.9	putative nucleotidyltransferase
SCO4550	98.4	0.9	conserved hypothetical protein
SCO4813	97.7	0.87	phosphoribosylglycinamide formyltransferase PurN
SCO4824	98.4	0.89	bifunctional protein (methylenetetrahydrofolate dehydrogenase and methenyltetrahydrofolate cyclohydrolase FolD
SCO5113	98.4	0.9	BldKB, putative ABC transport system lipoprotein,
			
**Negative correlation (all in set C)**			
SCO1947:683	98.4	-0.86	glyceraldehyde-3-phosphate dehydrogenase Gap1
SCO2198:235	97.7	-0.87	glutamine synthetase I GlnA
SCO2198:746	98.8	-0.92	glutamine synthetase I GlnA
SCO2634	97	-0.85	conserved hypothetical protein
SCO2736:472	95.1	-0.83	Citrate synthase CitA
SCO5466	97.8	-0.87	putative hydrolase

Congruence across time was calculated by Spearman Rank Correlation given different data sets where set A represents 5 time points in M600, B represents 5 time points in Δ*bldA *and C represents 10 time points spanning both M600 and Δ*bldA *(the 5 time points are 1, 2, 4, 6 and 8 in Fig. 1).

Comparing Table 1 and Additional File 5, only eleven *bldA*-influenced genes were found by both approaches (six when considering only the statistically significant proteome differences). However, at least 53 of the 74 significantly differentially transcribed genes encode proteins that are predicted to be undetected in the 2D gel proteomics approach employed, because of their physical properties (basic pI, low molecular weight (<13 KDa), multiple transmembrane domains or putative secretion: (see Table 1)). Indeed, four of the genes in Table 1 that encode putatively secreted protein products (SCO0297, 0762, 1968 and 6198) and are down-regulated in *bldA*, were reported to be less abundant in the mutant extracellular proteome [8]. Taking this into account, there were ten discrepancies involving failure of the proteome analysis to find genes picked out in the transcriptome study, and this may be associated with limitations of the proteomics approach for detecting proteins of low abundance. In addition, the higher number of replicates and sampling points in the transcriptomics study will have increased the power of this analysis for determining statistically significant changes. Some changes in the proteome were not picked up by transcriptome analysis. Many of these may reflect either post-translational processes of potential physiological significance (e.g. proteins in Table 2), or differing stabilities of the mRNA and corresponding protein products of gene expression. Thus, there are real benefits in using a combination of proteome and transcriptome analyses to assess global gene expression, where limitations in one technique tend to be compensated by strengths of the other. Because of its sensitivity and genuinely global nature, the transcriptome approach captures data on lowly expressed genes, and on secreted, membrane or high molecular weight gene products, which are all poorly represented in whole-cell proteomics data. Proteomics on the other hand informs on the abundance of the end-product of gene expression, encompassing the effects of both post-transcriptional and post-translational regulatory mechanisms that cannot be glimpsed through transcriptomics.

## Conclusion

### The role and evolution of a *bldA*-based checkpoint for *Streptomyces *differentiation

Taking into account the sum total of information now available about the effects of *bldA *mutations, and the occurrence and nature of TTA-containing genes in streptomycetes, we suggest the following *bldA*-centred model of *Streptomyces *physiology.

Nutrient-limited conditions in most bacteria, including streptomycetes, are signalled through a mechanism involving ppGpp that generally results in decreased production of the machinery for macromolecular synthesis, including tRNAs. In the case of streptomycetes, such conditions may apply either when the environment changes, or in centrally located parts of the colony in which nutrient demands have come to outstrip the rate at which external supplies can diffuse in. It is in the latter parts of the colony that morphological and physiological differentiation take place. The transcription of the *bldA*-specified pro-tRNA and its processing to a mature form are increased in these conditions, as if *bldA *responds to nutrient limitation in the opposite way from other translational components. Indeed, nutritional shiftdown-induced increases in ppGpp concentration that strongly inhibited transcription of the *rrnD *geneset for 16S rRNA did not inhibit *bldA *transcription and processing [34].

A relative increase in the level of *bldA *tRNA provides an intracellular environment suitable for the translation of UUA-containing mRNA. In view of our evidence suggesting that such mRNAs have reduced abundance when the translation of their UUA codons is restricted, their free translation would increase the abundance of the messages, potentially further increasing the levels of the protein products. This enhancement may apply to individual structural genes or whole operons (such as the deoxysugar cluster SCO0381-0401), causing rapid changes in the activity of the corresponding biochemical activities and pathways. Such pathways nearly all show some degree of species-specificity, and many of them probably confer adaptive benefits in very particular environmental circumstances [26]. The enhancement should also apply to the expression of TTA-containing regulatory genes. Some of these activate pathway gene sets for antibiotic biosynthesis, and one, *adpA*, activates some critical aspects of morphological differentiation, at least partially by influencing an extracellular protease cascade [8]. Others activate other, often still uncharacterised, gene sets (such as the SCO4251-4253 operon). We presume that many of the processes showing some dependence on *bldA *are also subject to other regulatory influences, and that the role of *bldA *is to provide one of possibly several checkpoints that must be achieved before commitment to particular physiological activities, such as the onset of secondary metabolism or the activation of substrate mycelium autolysis that is associated with reproductive aerial growth.

If exposure of UUA-containing mRNA to ribosomes limited for *bldA *tRNA elicits ppGpp synthesis as our results suggest, this could reduce the efficiency of transcription of any genes adapted for expression in conditions favouring very rapid growth. Many bacteriophage genes have such adaptations. It could be imagined that the presence of UUA codons in highly expressed genes of *Streptomyces *phages would reduce the effective expression levels of such genes, providing a possible selective route for the evolution of the *bldA *checkpoint.

## Methods

### Strains, growth conditions and sampling

*S. coelicolor *M600 is a prototrophic, plasmid-free strain of *S. coelicolor *A3(2) [6]. In the M600 Δ*bldA *strain, kindly provided by M. Tao, *bldA *is completely replaced by an apramycin resistance cassette. Strains were cultivated with vigorous agitation at 30°C in minimal medium supplemented with 0.2 % casamino acids (SMM) as previously described [35]. In summary, spores (about 10^10 ^colony-forming units ml^-1^) were pre-germinated in 2 × YT medium [35] for 7 h at 30°C. Germlings were harvested by centrifugation (5 min at 4000 × g), resuspended in SMM, and briefly sonicated to disperse any aggregates, before inoculation into 50 ml of SMM in 250 ml siliconised flasks containing coiled stainless steel springs. Each flask received the equivalent of 5 × 10^7 ^colony-forming units. Growth curves for producing RNA and protein extracts to compare M600 and M600 Δ*bldA *strains were performed in triplicate, with 35-ml samples being taken from cultures of each strain at eight culture ages encompassing the exponential, transition and stationary phases (Fig. 1). Typically, 10 ml of each sample was used for RNA extraction and 25 ml for proteomic analysis. All samples from all three time-series were subjected to transcriptome analysis, while for logistical reasons only samples 1, 2, 4, 6 and 8 from two of the time-series were analysed using proteomics.

### Transcriptomics Methods

#### i) Genomic DNA and RNA extraction

M145 genomic DNA was prepared by the 'Kirby mix' method described in Kieser *et al. *[35], except that sarkosyl replaced TPNS as the detergent. To retain RNA content and integrity, the mycelium from each 10 ml culture sample was treated with RNA Protect Bacteria Reagent (Qiagen), following the manufacturers' recommendations, and then stored at -20°C. The procedure used for RNA extraction was as described at [36] employing RNeasy purification columns from Qiagen. The RNA was eluted in a final volume of 250 μl, assessed for structural integrity using a Bioanalyser system (Agilent Technologies), and quantified spectrophotometrically using a NanoDrop ND-1000 instrument (Labtech). The A_260_/A_280 _absorption ratios of the extracted RNA were between 1.8 and 2.

#### ii) Nucleic acid labelling and microarray hybridization

The procedures for genomic DNA labelling and array hybridizations were as described at [36]. Briefly, for the cDNA synthesis and labelling, 15 μg of total RNA was added to a reaction mixture containing random hexamer primers (Invitrogen), Superscript II Reverse Transcriptase (Invitrogen) and Cy3-dCTP (GE Healthcare), and the mixture was incubated at 42°C for 2–4 h. For the labelling of genomic DNA, ca. 3 μg DNA was labelled in a 50 μl reaction volume with Klenow enzyme and Cy5-dCTP. The incorporated Cy-dye-dCTP was quantified using a Nanodrop ND-1000 spectrophotometer. A volume equivalent to 45 pmol of Cy3-dCTP-labelled cDNA was pooled with 20 pmol of Cy5-dCTP-labelled genomic DNA, dried in a vacuum centrifuge and resuspended in 45 μl of Pronto! Universal Hybridisation Solution for long oligos and cDNA (Corning). For the array hybridisations of biological replicates 1 and 2, a salt-based hybridisation solution was used instead of a formamide-based solution [37].

The microarrays were coated glass slides (Corning GAPS II for replicates 1 and 2 and Corning UltraGAPS for replicate 3) spotted with PCR amplicons covering ca. 92% of *S. coelicolor *ORFs. The array design and layouts used are described at [36]. Different array layout and print runs were used in this work: for the biological replicates 1 and 2 the arrays contained one probe spot for each gene (array batches Sc9 and Sc10) while the arrays for the third biological replicate contained duplicate probe spots (batch Scp28). Microarrays were scanned using an Affymetrix 428 dual laser scanner. The microarray spots were analysed and quantified using BlueFuse software (Version 3.1; BlueGnome Ltd, Cambridge), which uses statistical parametrical models for accurate quantification of the spot signal and background noise.

### Proteomics Methods

#### i) Preparation of protein extracts from cultures

Mycelium was harvested from 25ml samples of cultures by centrifugation (30 sec, 4000 × g, room temperature) and immediately frozen in liquid nitrogen, with a transfer time from culture flask to frozen sample of 1.5 minutes. Mycelial pellets were stored at -80°C until use. Protein extracts, prepared as in Hesketh *et al. *[18], dissolved in denaturing isoelectric focusing buffer UTCHAPS (7 M urea, 2 M thiourea, 4% (w/v) CHAPS, 50 mM DTT, 4 mM Pefabloc SC protease inhibitor, and 40 mM tris pH9.0), were stored frozen in aliquots at -80°C.

#### ii) 2D gel separation of proteins, spot quantification and spot identification

Protein extracts were subjected to 2D gel electrophoresis over the pH4.5-5.5 or pH5.5-6.7 isoelectric point ranges as detailed in Hesketh *et al*. [18]. For quantitative analysis of protein abundance profiles, gels were stained with Sypro Ruby (Bio Rad) according to the manufacturer's instructions, and scanned using the Perkin-Elmer ProXPRESS proteomic imaging system using excitation and emission wavelengths of 480 nm and 630 nm, respectively. To produce a quantitative analysis of protein abundance profiles, gel images were analysed using PHORETIX 2D version 5.1 (NonLinear Dynamics): spot detection was optimised automatically using the 'Spot Detection Wizard' and then manually edited; background subtraction was performed automatically using the 'Mode of Non-Spot' setting; and images were then normalised to the total spot volume for each gel for quantification. Spot filtering was not used, although all spots were manually edited. Histograms of normalised spot volumes displaying changes in spot abundance during growth and between M600 and the Δ*bldA *mutant were generated within the software. Protein spots of interest were excised from Sypro Ruby-stained gels using the Investigator ProPic robot from Genomic Solutions, and identified by tryptic digestion and MALDI-ToF mass spectrometry as previously described [18]. Identification of proteins from peptide mass fingerprint data was performed using the MatrixScience 'Mascot' search engine [38], and was based on their 'Probability Based MOWSE Score' algorithm. A MOWSE score of 60 or higher is significant at the 5% level or better, and proteins in this work typically gave scores > 80 (frequently considerably higher). In addition, no identification was accepted unless at least 5 peptides representing at least 20% of the protein sequence were detected in the MALDI-ToF peptide mass fingerprint. Spots selected for identification included not only those showing significant differences between strains (using the non-statistical approach outlined below), but also some exhibiting only growth-phase dependent changes in abundance, and a smaller number of landmark spots that were neither growth-phase nor strain dependent.

### Preprocessing of -omics data

#### Microarray data

For normalization methods the statistical computing environment R (Version 2.1.1) [39] and the package Limma [40] were used. Within-array global median normalisation of log_2 _cDNA/gDNA ratios was applied to the data from each array in the analysis. Control and flagged spots were ignored. The log_2 _ratios were then scaled to have the same median-absolute-deviation (MAD) across arrays [40,41] of the same replicate series (I, II and III). Only un-flagged data (6,457 genes) were used in differential expression analyses and in the averaging of replicate data for the transcriptome-proteome correlation analysis.

#### Proteomics data

The proteomics data were analysed using both statistical and mathematical approaches. For the mathematical methods, normalized spot volumes as generated within the Phoretix 2D software (see above) were used without further processing. For all statistical analyses, these normalized spot volume measurements were transformed to the log_2 _scale, with an arbitrary value of 1 being assigned prior to transformation to protein spots too low in abundance to be detected.

### Public availability of -omics data

The transcriptome data have been deposited in the MIAME-compliant ArrayExpress database with accession number E-MAXD-27 [42]. Proteome data are listed in Additional File 4 as an Excel file of normalised spot volumes for all 336 identified spots.

### Data analysis

#### Transcriptomics: genes expressed differentially between strains

The choice of analytical method when compiling a list of differentially expressed genes between conditions is dependent on numerous factors [43]. For the transcriptomic data two independent methods were used: Welch t-test and Rank Product analysis [44]. Whilst t-test based approaches are commonly used for analysing microarray data it is apparent that when there is large variance between biological replicates (as is the case with *Streptomyces *cultures) and/or crucial single time-point changes (in this analysis), the t-test has shortcomings [[43,44], this study]. Consequently, the more 'biology friendly' technique of Rank Products [44] was also used. Comparison of the gene lists produced when applying the respective thresholds (Welch t-test p-value < 0.05, Rank Product pfp value < 0.1) revealed little overlap between them. Hence, to avoid biasing any interpretation of the results the lists generated by the two techniques were combined.

#### Welch t-test

The preprocessed data were imported into Genespring 7.2 (Agilent technologies) for further analysis and genes deemed to be 'non-changing' were filtered out from the un-flagged transcriptome data by removing those with log_2 _expression values between -0.667 and 0.667 in all 16 time points (eight in M600, eight in Δ*bldA*). The remaining 5,983 genes were then analysed for significant (p < 0.05) differential expression between M600 and Δ*bldA *using the Welch t-test and Benjamini and Hochberg multiple testing correction [45].

#### Rank Product analysis

The preprocessed data (6,457 genes) were imported into R (Version 2.1.1) [39] for analysis using the RankProd package [46] with default parameters. Though the microarray data produced in this study are two-colour, the use of a genomic reference allowed the use of the RankProd package analagously to one-colour data, the values compared being log_2 _expression ratios of sample_cDNA/gDNA. Thus, to compare the *bldA *mutant to M600 at a particular time point six expression ratios were used, three from the mutant and three from M600. Rank products (and associated p-values, probability of false prediction (pfp) values and average fold-change) for both up- and down regulation were then calculated by using ranks for each pairwise comparison of *bldA*/M600 (i.e. *bldA*_repA/M600_repA, *bldA*_repA/M600_repB, *bldA*_repA/M600_repC etc). This was repeated for each time-point, comparing the *bldA *mutant replicates with their respective M600 counterparts. Differentially expressed up- and down-regulated genes were selected if their corresponding pfp value was ≤ 0.1.

#### Proteins represented differentially between strains

To test statistically for proteins represented differentially between M600 and Δ*bldA *the non-parametric Mann-Whitney U-test was applied, comparing the data based on their ranks rather than absolute values. All 336 protein spots were tested for significance of differences between strains, with p-values being corrected by the Benjamini and Hochberg procedure. Differences were deemed significant if the corrected p-value was less than 0.05. It was evident that using these stringent criteria has the potential to lose interesting data, particularly spots absent in one strain and developmentally regulated in the other, since any protein spot completely absent from one would have to be present in at least three of the five time-points of the second strain to be called significantly different (based on simulations, see below). An additional complementary mathematical analysis was therefore undertaken in which protein spots were considered to be differentially represented between strains if their normalized volumes were changed two-fold or more in both biological duplicate growth curves in at least two (of the five) corresponding time points. The results from both approaches were ultimately combined to produce a master list of differentially represented proteins, with presence and absence of corresponding P-values indicating which protein spots were found in the statistical and mathematical analyses, respectively.

#### Classification of proteins according to their abundance profiles in M600

Normalized spot volume measurements for strain M600 samples over time for all 336 identified proteins were grouped into one of six abundance profile classes by applying the following set of simple mathematical rules. If in each replicate the largest value is less than or equal to the smallest value multiplied by 2, place into Group A (approximately equal abundance). If this is true only for one replicate, place in Group F (approximately equal abundance at each timepoint in one growth series, but not the other). The remainder exhibit two-fold or more changes in abundance in both replicates, and are placed into Group B if they reproducibly exhibit increasing abundance with time, Group C for those decreasing in abundance with time, and Group D for those with an initial increase followed by a decrease. Any remaining are placed in Group E (changing, but no reproducible trend). Ten of the 336 spots were not seen in M600 but were detected in the *bldA *mutant, and are given a separate class.

#### Correlation between transcriptome and proteome

Spearman Rank correlation was used to compare the transcriptome and proteome mainly because it avoids scaling issues. As a non-parametric method, using the ranking of values to compute correlation Spearman rank also allows the use of arbitrary values representing very low abundance proteins. Thus, the Spearman rank correlation r between transcript and protein was calculated by

r=1−6∑sNdg,s2N(N2−1)
 MathType@MTEF@5@5@+=feaafiart1ev1aaatCvAUfKttLearuWrP9MDH5MBPbIqV92AaeXatLxBI9gBaebbnrfifHhDYfgasaacH8akY=wiFfYdH8Gipec8Eeeu0xXdbba9frFj0=OqFfea0dXdd9vqai=hGuQ8kuc9pgc9s8qqaq=dirpe0xb9q8qiLsFr0=vr0=vr0dc8meaabaqaciaacaGaaeqabaqabeGadaaakeaacqWGYbGCcqGH9aqpcqaIXaqmcqGHsislcqaI2aGndaaeWbqaamaalaaabaGaemizaq2aa0baaSqaaiabdEgaNjabcYcaSiabdohaZbqaaiabikdaYaaaaOqaaiabd6eaojabcIcaOiabd6eaonaaCaaaleqabaGaeGOmaidaaOGaeyOeI0IaeGymaeJaeiykaKcaaaWcbaGaem4CamhabaGaemOta4eaniabggHiLdaaaa@43FE@

where *d *is the difference between the ranks of protein and transcript data in sample *s *and *N *is the number of samples.

To test whether the observed Spearman rank correlation could be obtained by chance a t-test was calculated by

t=r1−r2N−2
 MathType@MTEF@5@5@+=feaafiart1ev1aaatCvAUfKttLearuWrP9MDH5MBPbIqV92AaeXatLxBI9gBaebbnrfifHhDYfgasaacH8akY=wiFfYdH8Gipec8Eeeu0xXdbba9frFj0=OqFfea0dXdd9vqai=hGuQ8kuc9pgc9s8qqaq=dirpe0xb9q8qiLsFr0=vr0=vr0dc8meaabaqaciaacaGaaeqabaqabeGadaaakeaacqWG0baDcqGH9aqpdaWcaaqaaiabdkhaYbqaamaakaaabaWaaSaaaeaacqaIXaqmcqGHsislcqWGYbGCdaahaaWcbeqaaiabikdaYaaaaOqaaiabd6eaojabgkHiTiabikdaYaaaaSqabaaaaaaa@3842@

The resultant t-statistic was then converted to a p-value based on the t-distribution. All p-values were then corrected by application of the Benjamini and Hochberg multiple testing correction procedure [39,45,47] and were classed as significant if p < 0.05. Correlation between transcriptome and proteome data was computed for each of the 5 time-points in each strain (a vector of 310 in each test) and for each gene across five time-points in M600, five time-points in Δ*bldA *and ten time-points across both M600 and Δ*bldA*. Analysis was only performed on 310 protein spots, as the other 26 spots did not have corresponding genes that passed through the stringent filtering applied to the transcriptome data. Where multiple protein products were identified from a single gene the correlation was calculated using the transcriptomic data for that gene versus proteomic data for each individual protein spot (i.e. multiple spots were not averaged or summed), because of the possibility that not all spots for any one gene product were detected.

#### Identification of DNA motifs upstream of differentially expressed genes

Initial searches for conserved sequence motifs were conducted on the genes whose transcription was shown directly by Welch t-test analysis of microarray data to be influenced by *bldA*. A sequence set was constructed consisting of 500 nt segments (300 nt upstream and 200 nt downstream of the translational start codons) of each gene, in the correct transcriptional orientation (based on the annotated *S*. *coelicolor *genome GenBank (AL645882)). The sequence set was submitted to the MEME (Multiple Em for Motif Elicitation) server [48], applying default settings except for: minimum width of the motif to search for was set to 5 nts; maximum width to 30 nts; and maximum number of sites to find was set to 10. From the MEME output file an IUPAC consensus sequence was constructed for each motif. To estimate the distribution of each motif (i.e. how many genes in the genome have this motif in their upstream regions) the MEME-derived consensus sequence was used to search the *S. coelicolor *genome sequence for hits within the upstream regions of all genes (as defined above) using the genome-scale dna-pattern tool of RSAT (Regulatory Sequence Analysis Tools; [49]) with default settings except that one nucleotide substitution was allowed.

#### Construction of a SCO4263 deletion mutant

A mutant SCO4263 allele, in which the entire coding region was replaced by an apramycin resistance cassette, was constructed by PCR-targeting essentially as described by Gust *et al. *[50]. Briefly, oligonucleotide primers (5'-) with 5' ends homologous to the 5' and 3' ends adjacent to the SCO4263 coding sequence, and 3' (priming) ends designed to amplify the apramycin resistance disruption cassette, were used in a PCR with pIJ773 as template. The PCR product was introduced into *E. coli *BW25113/pIJ790 containing cosmid StD86A [51], preinduced for λred functions by the addition of arabinose, to obtain a SCO4263-disrupted version of the StD86A cosmid. The disrupted cosmid was introduced into *E. coli *ET12567/pUZ8002 by transformation, then transferred into *S. coelicolor *M600 by conjugation. Exconjugants were selected on MS agar [35] containing apramycin, and the products of double crossovers identified by screening for kanamycin sensitivity. Deletions were confirmed by PCR and Southern blotting.

#### Quantitative real-time PCR analysis of selected differentially expressed genes

Specific primers and probes for 14 of the 74 selected differentially expressed genes (SCO6808, SCO7657, SCO4295, SCO5013, SCO3088, SCO3285, SCO3286, SCO5166, SCO6958, SCO6638, SCO4648, SCO4702, SCO4705, SCO4717) were designed using either Primer Express v.2 software (Applied Biosystems) or Primer 3 software [52]. Five μg samples of each of the 16 RNA preparations from replicate 3 were subjected to RNase-free DNase I treatment (Promega) in a 20 μl reaction volume. One μg of RNA was used as template for cDNA synthesis with 225 ng of random hexamers (Invitrogen) and 50 units of Superscript II reverse transcriptase (Invitrogen) in 20 μl reaction volumes, and incubated for 10 min at 25°C, 50 min at 42°C and then 15 min at 70°C. The samples were diluted 1:4 with distilled water and 5 μl was used in the quantitative PCR reaction with 10 pmol of forward and reverse primer and 2.5 pmol of FAM/TAMRA dual-labelled specific probe (Operon technologies) in 25 μl master mix (QPCR ROX, ABgene). Parallel reactions were performed in the same 96-well plate using different dilutions of genomic DNA in order to generate a standard curve for each selected gene. The reactions were analysed in an ABI PRISM 7000 Sequence Detection System (Applied Biosystems).

#### S1 nuclease mapping

RNA was isolated from exponential and transition-phase cultures as described by Strauch *et al*. [12]. For each S1 nuclease reaction 20 μg RNA was hybridised in NaTCA buffer [53] to about 0.2 pmol (approximately 10^5 ^Cerenkov counts min^-1^) of each of the following radiolabelled probes. For SCO4253, the oligonucleotide 5'-GACCGACGAAGGCCGCCACCGA-3', which anneals within the SCO4253 coding region, was uniquely end-labelled at its 5'- end with [γ-^32^P]-ATP using T4 polynucleotide kinase. This was used in the PCR together with the unlabelled probe 5'-GATCTGACCGATCCTCCTGACACGCCGTCACCGT-3' (which anneals upstream of the SCO4253 gene) and cosmid StD49 as template, to generate a 432 bp probe. PCR was performed in the presence of 7% dimethylsulphoxide using the following conditions: 94°C for 4 min followed by 26 cycles of 45 s at 94°C, 45 s at 58°C and 60 s at 72°C, then held at 72°C for 5 min to finish. The probe for SCO0762 was made in a similar way, but using 5'- AGGCGATCCTAGTCGATCAAGAAACGCCCAGTTC-3' as the unlabelled primer, 5'-TCTCTCCGTGCCCCACGGTCAGCA-3' as the labelled primer, and cosmid StF81 [51] as template. This produced a 365 bp uniquely end-labelled probe product. Hybridisations were carried out at 45°C for 14 h after denaturation at 65°C for 15 min. S1 nuclease digestions and analyses of RNA-protected fragments were performed as described previously [54]. High-resolution mapping of the transcription start site of SCO4235 was achieved by generating a sequencing ladder with the same labelled primer as was used for the probe, using a Thermo Sequenase cycle sequencing kit (USB) according to the manufacturer's instructions.

#### Quantification of ppGpp and intracellular nucleotides

Extraction and HPLC analysis of ppGpp, ATP and GTP from *S. coelicolor *cultures were carried out as described by Strauch *et al. *[12].

## Authors' contributions

AH performed the microbiological work, the proteomic analysis (including some data analysis), and the ppGpp and S1 mapping studies. GB performed the transcriptomic analysis and qPCR (including some data analysis). EL performed the bulk of the data analysis. FF participated in the transcriptome analysis. GH was involved in microarray design and production, and participated in preliminary data analysis. CPS and KFC conceived of the study, and participated in its design and coordination. AH, GB, EL, CPS and KFC all contributed to the drafting of the manuscript.

## Supplementary Material

Additional file 1 Transcript abundance profiles for all 74 genes identified as being differentially expressed between M600 and M600 Δ*bldA*. Provides a summary of the transcript abundance profiles for all 74 genes identified as being differentially expressed between M600 and M600 Δ*bldA *using DNA microarrays. Click here for file

Additional file 2 Transcription profiles determined for SCO4295, SCO5013, SCO6808, SCO7657, SCO 3088, SCO 3285, SCO 3286, SCO 5166, SCO 6958, SCO 6638, SCO 4660, SCO 4702 and SCO 4648 using either a) Q-RT-PCR, or B) DNAmicroarrays. A comparison for a subset of genes of transcript abundance profiles determined using DNA microarrays with results obtained using quantitative RT-PCR. Click here for file

Additional file 3 A list of ribosomal protein genes present in the *S. coelicolor *genome, and the predicted physical properties of their gene products. Illustrates that the vast majority of ribosomal proteins would not be expected to be detected in the protomics analysis undertaken in this study. Click here for file

Additional file 4 Normalized spot volume data for the 336 protein spots identified in the proteome analysis. A spreadsheet of quantitative protein spot measurements from the proteome analysis. Click here for file

Additional file 5 Summary of the protein spots identified as being differentially represented between M600 and M600 Δ*bldA*. Lists proteins that were found to be statistically differentially represented between the two strains using the Mann-Whitney test; those found to be reproducibly differentially expressed using the complementary mathematical approach detailed in the Methods; and a combined list summarising all differences. Functional assignments are noted, based on KEGG [56], and on the Sanger Institute classifications [57], and information given on: (a) occurrence of more than one gene product per gene; (b) 2-fold up- or down-regulated relative to the *bldA *mutant; and (c) membership of the M600 expression profile group (see Additional File 6). Click here for file

Additional file 6 Classification of the time-course data for the 336 identified protein spots according to their abundance profiles in strain M600. Provides information on: (a) occurrence of more than one gene product per gene; (b) 2-fold up- or down-regulated relative to the *bldA *mutant; and (c) P-value for any statistically different protein spot observed. A list of all genes for which more than one protein spot was identified is also presented. Click here for file

Additional file 7 Time-course abundance profiles showing changes in the post-translational processing of certain proteins as a result of *bldA *mutation. Illustrates the changes observed in post-translational processing of 11 proteins during growth of the wild-type and bldA mutant strains. Click here for file

Additional file 8 Summary of the identification of changes in transcription or protein synthesis from 147 genes as a result of *bldA *mutation. Functional assignments are noted, based on KEGG [56] and on the Sanger Institute classifications [57], and operon membership is also indicated. Differences identified from the transcriptome and proteome analyses are listed separately, and also combined into a masterlist of all differences. Functional assignments are noted, based on KEGG [56] and on the Sanger Institute classifications [57]. For the proteome data information is given on: a) occurrence of more than one gene product per gene; b) 2-fold up- or down-regulated relative to the bldA mutant; and c) membership of the M600 expression profile group (see Additional File 6). Significance P-values are given where appropriate. Click here for file

Additional file 9 Comparison of transcript and protein abundance profiles for the eleven genes identified by both transcriptome and proteome analyses as being differentially expressed in the Δ*bldA *mutant. Compares the transcript and protein spot abundance profiles for the eleven genes identified by both transcriptome and proteome analysis as being differentially expressed in the *bldA *mutant. Click here for file

Additional file 10 Abundance data for protein spots belonging to genes responsible for the production of secondary metabolites in *S. coelicolor*. Illustrates differential representation of many proteins associated with secondary metabolite production as a result of *bldA *mutation. Click here for file

Additional file 11 Consensus DNA sequence motifs identified upstream of the 40 differentially transcribed genes determined from the ANOVA transcriptomics analysis. The top ten most significant motifs are listed, and figures for how frequently each motif is found in the genome are given. Click here for file
