# Supplementary material for: New pleiotropic effects of eliminating a rare tRNA from Streptomyces coelicolor, revealed by combined proteomic and transcriptomic analysis of liquid cultures
Source: BMC Genomics. 2007 Aug 2;8:261. doi: 10.1186/1471-2164-8-261 (PMC2000904; doi:10.1186/1471-2164-8-261)
Supplement: Additional file 1 — Transcript abundance profiles for all 74 genes identified as being differentially expressed between M600 and M600 ΔbldA. Provides a summary of the transcript abundance profiles for all 74 genes identified as being differentially expressed between M600 and M600 ΔbldA using DNA microarrays. [file 1471-2164-8-261-S1.pdf]

Additional file 1.
Transcript abundance profiles for all 74 genes identified as being differentially expressed between M600 and M600 Δ*bldA* using DNA microarrays.

## SCO0072

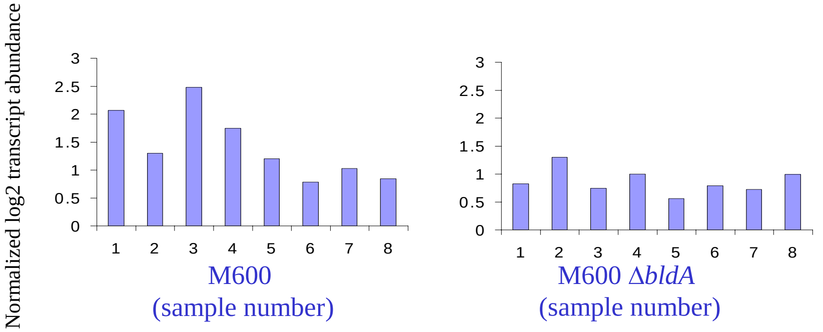


## SCO0247

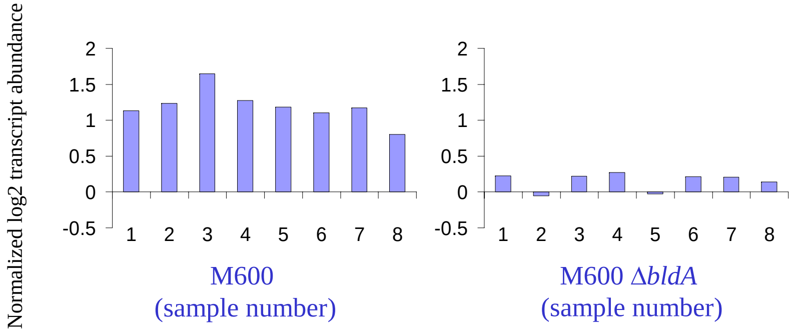


## SCO0248

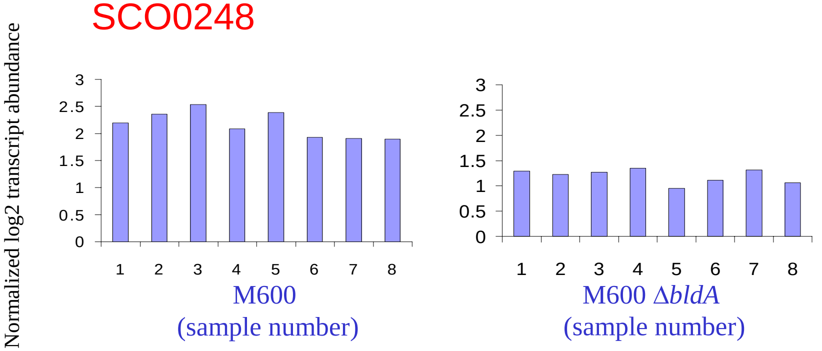


## SCO0297

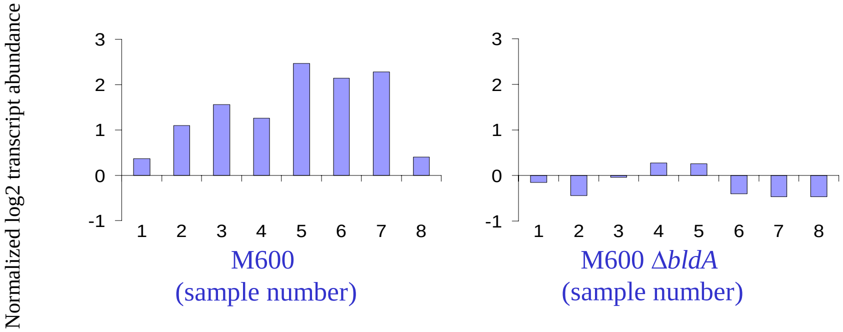


## SCO0472

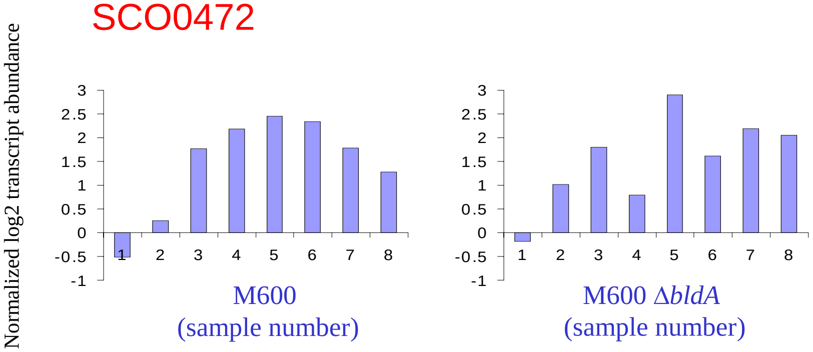


## SCO0499

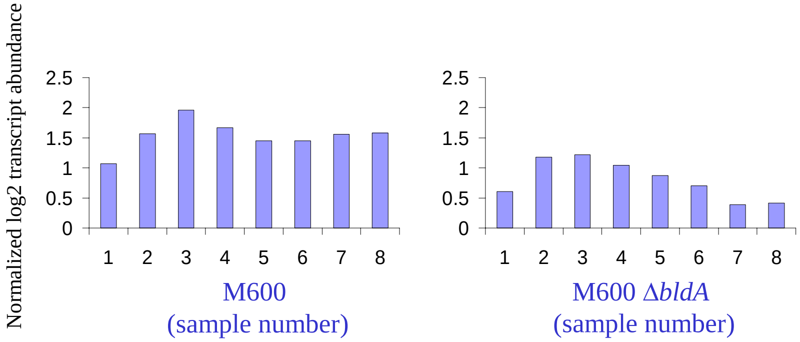


Profiles are the average of triplicate experiments

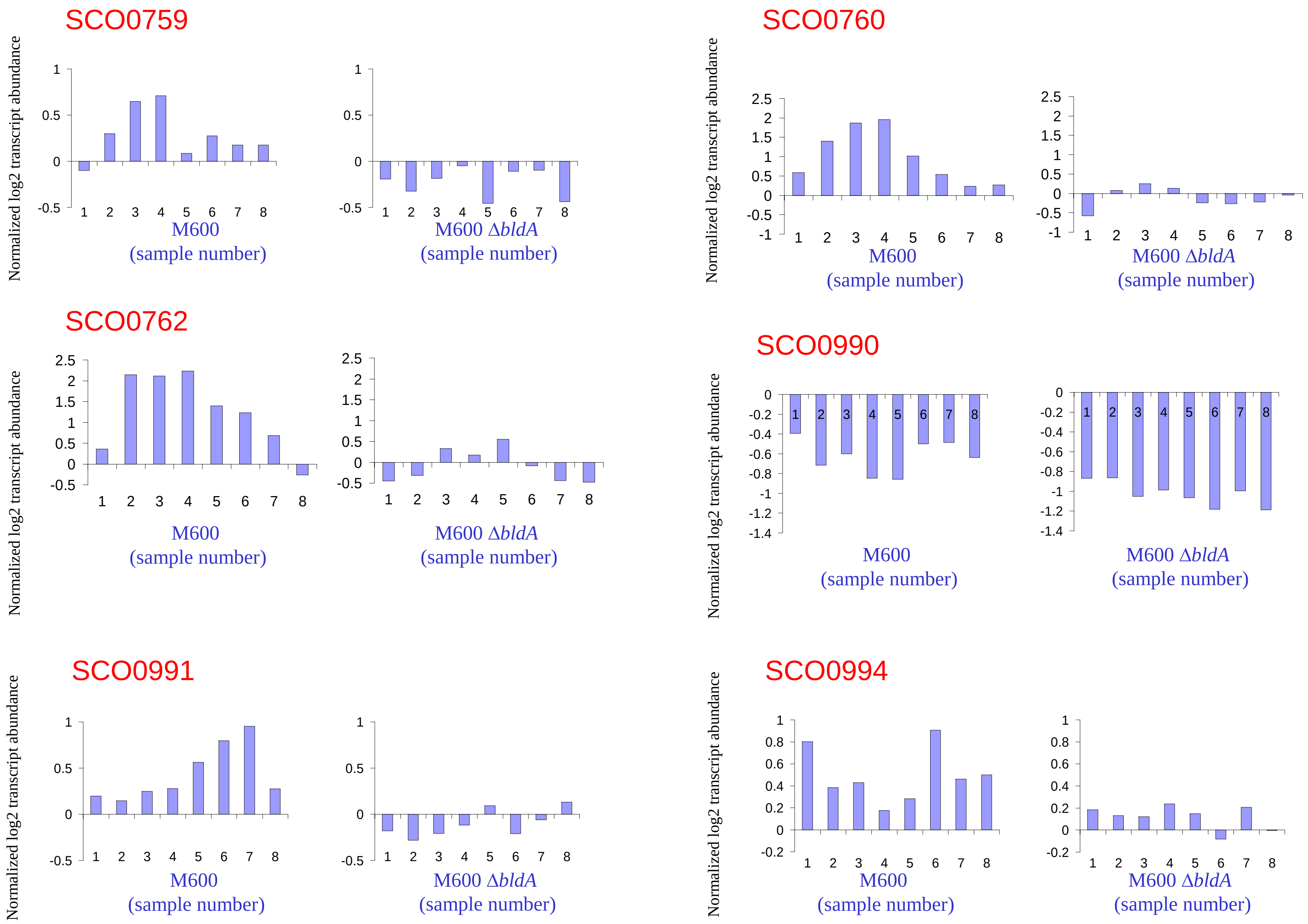


Profiles are the average of triplicate experiments

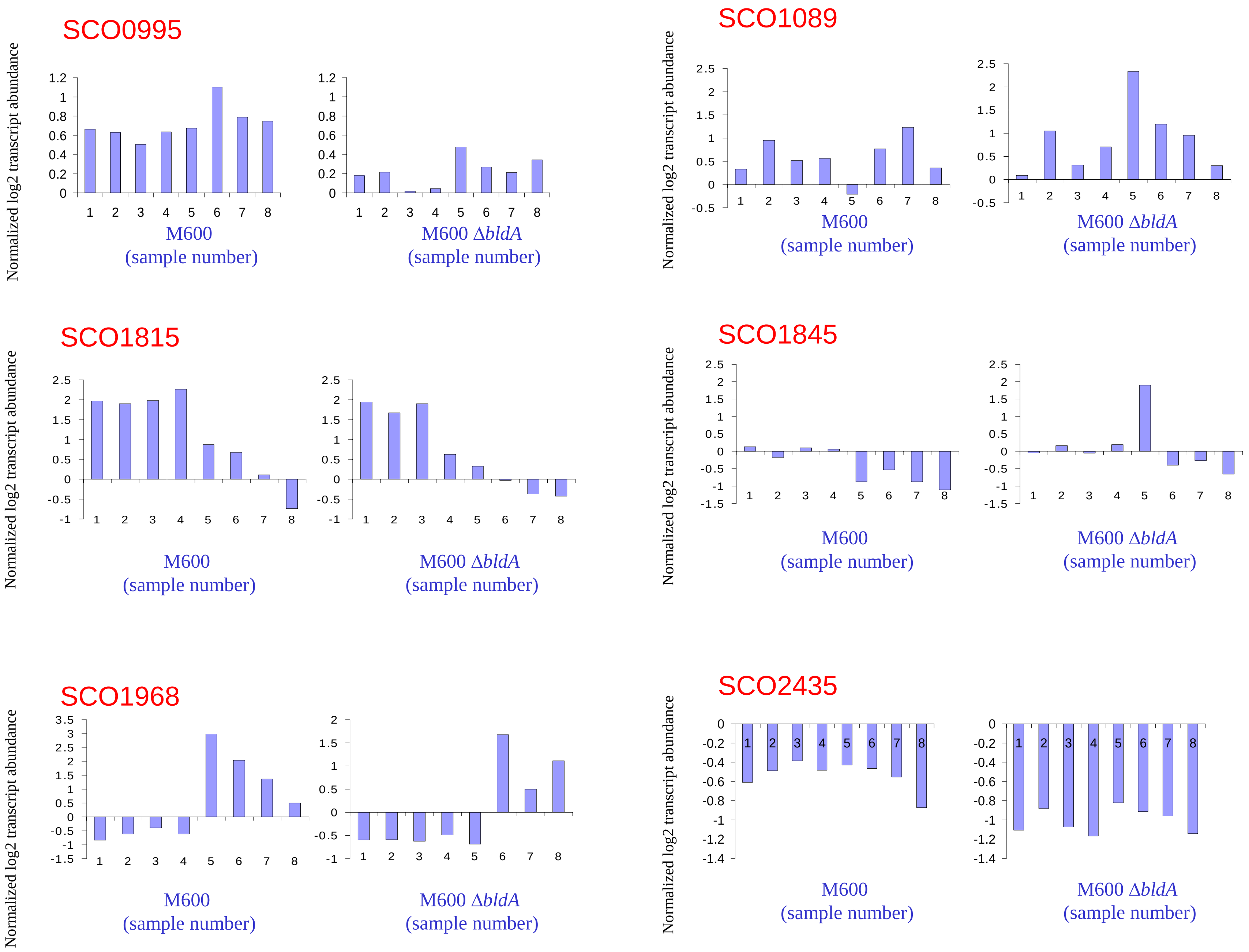


Profiles are the average of triplicate experiments

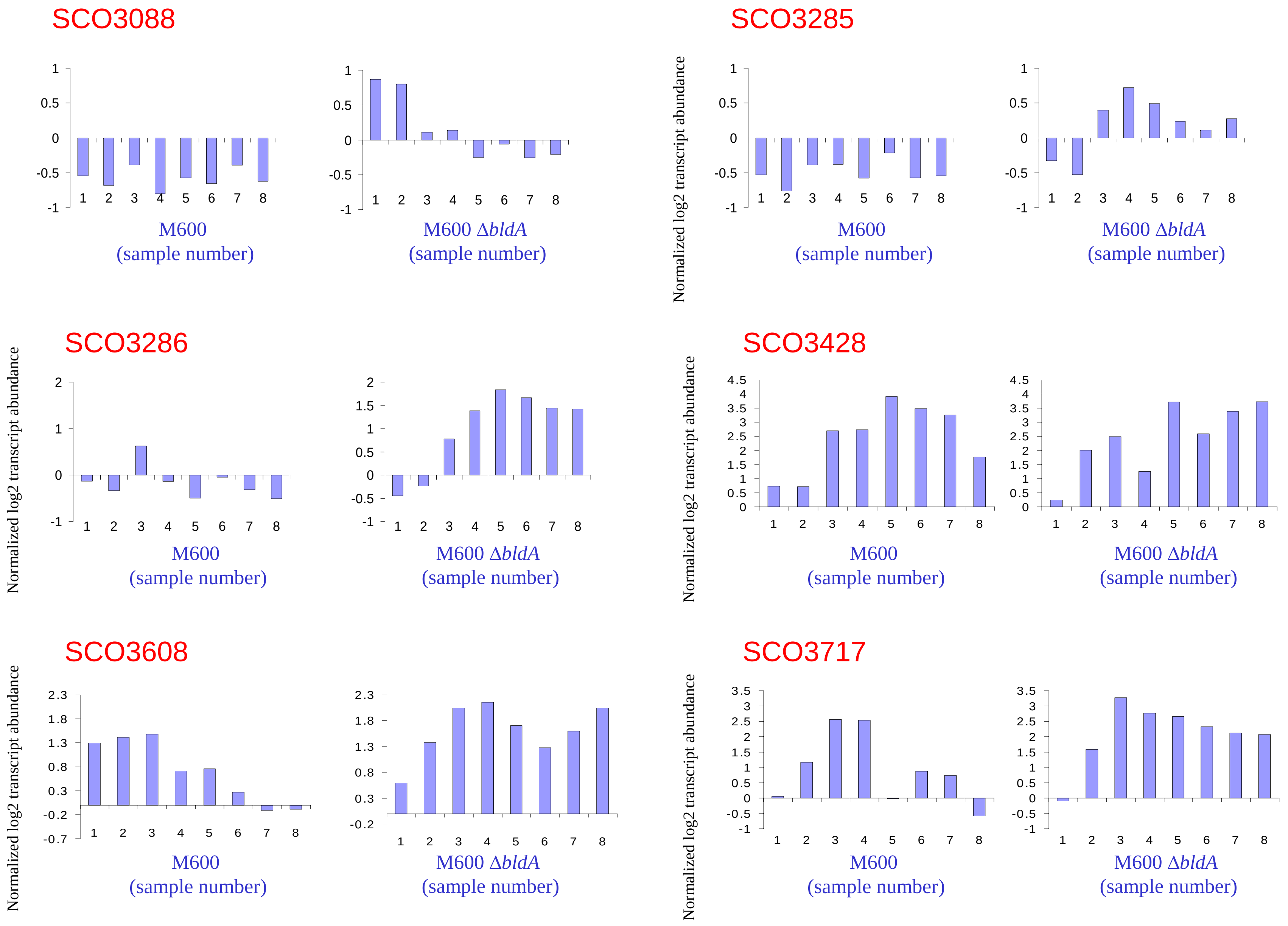


Profiles are the average of triplicate experiments

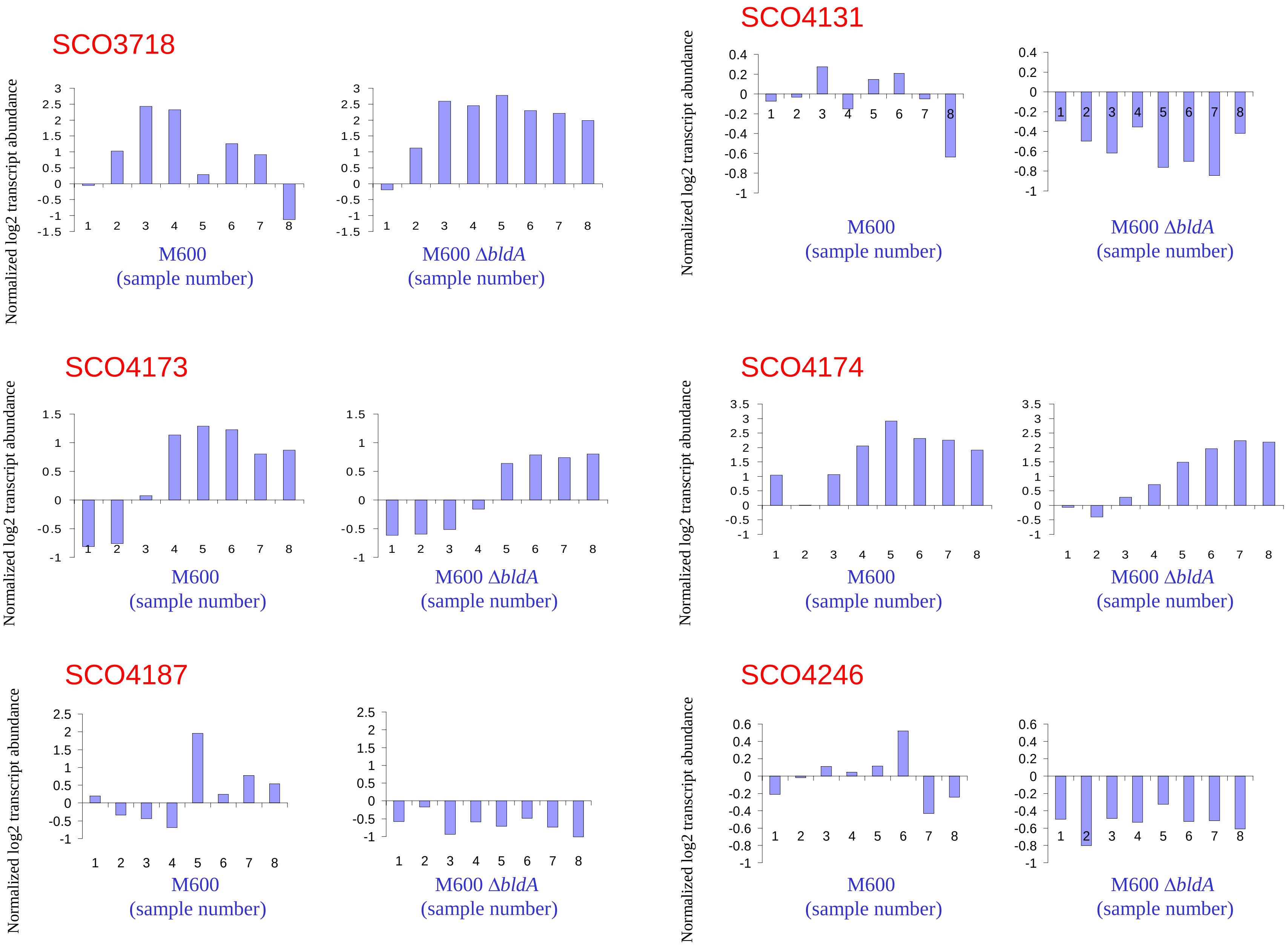


Profiles are the average of triplicate experiments

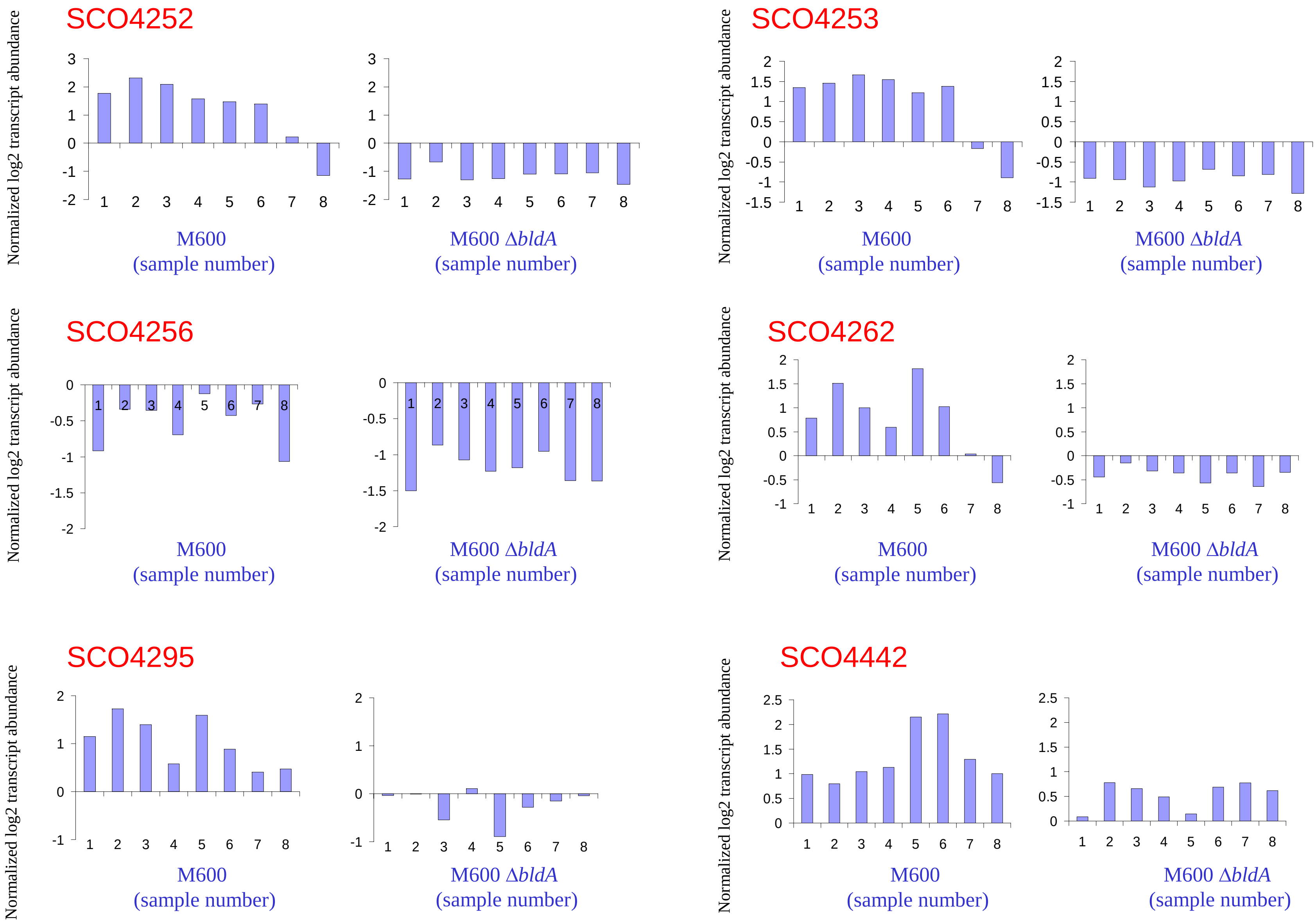


Profiles are the average of triplicate experiments

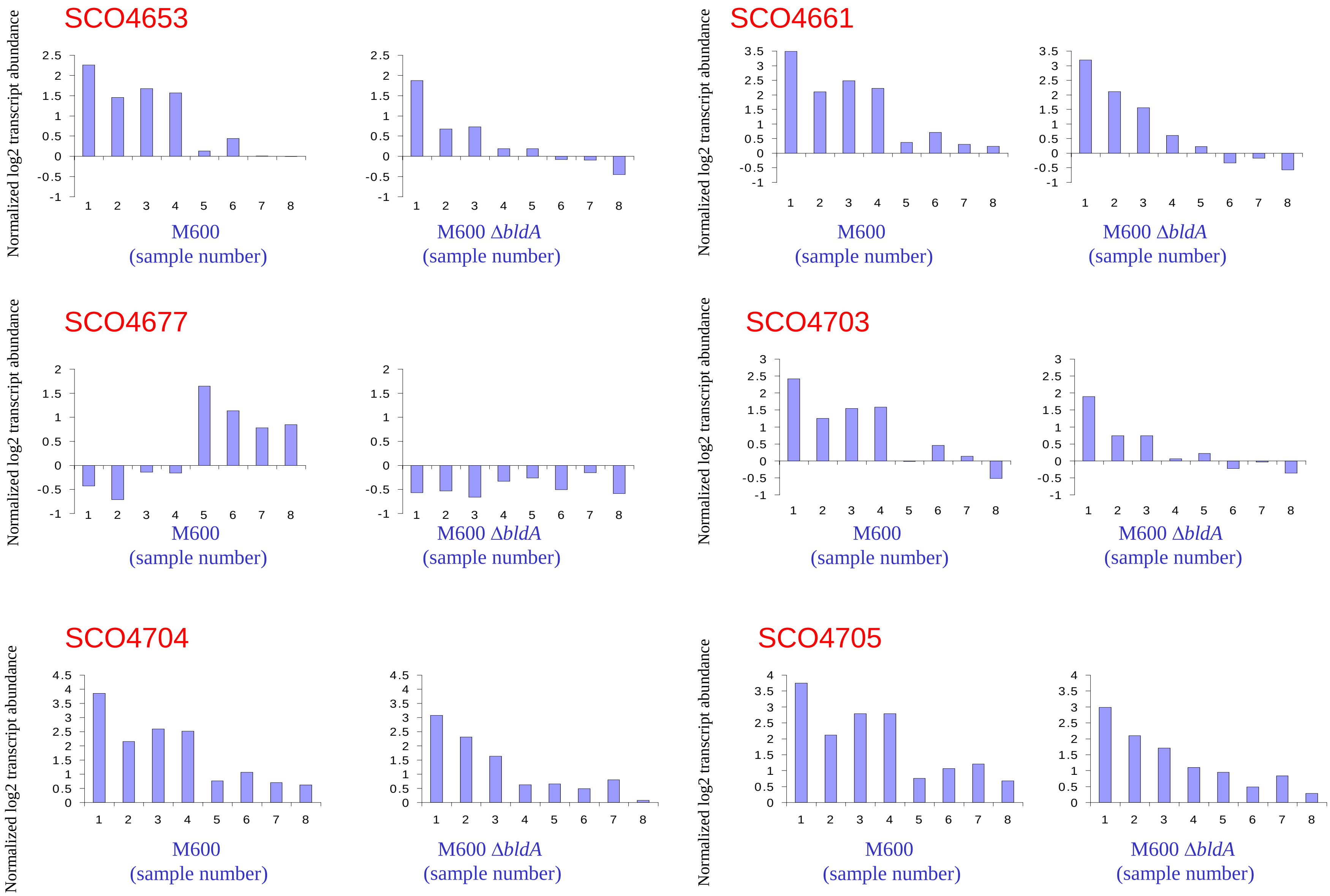


Profiles are the average of triplicate experiments

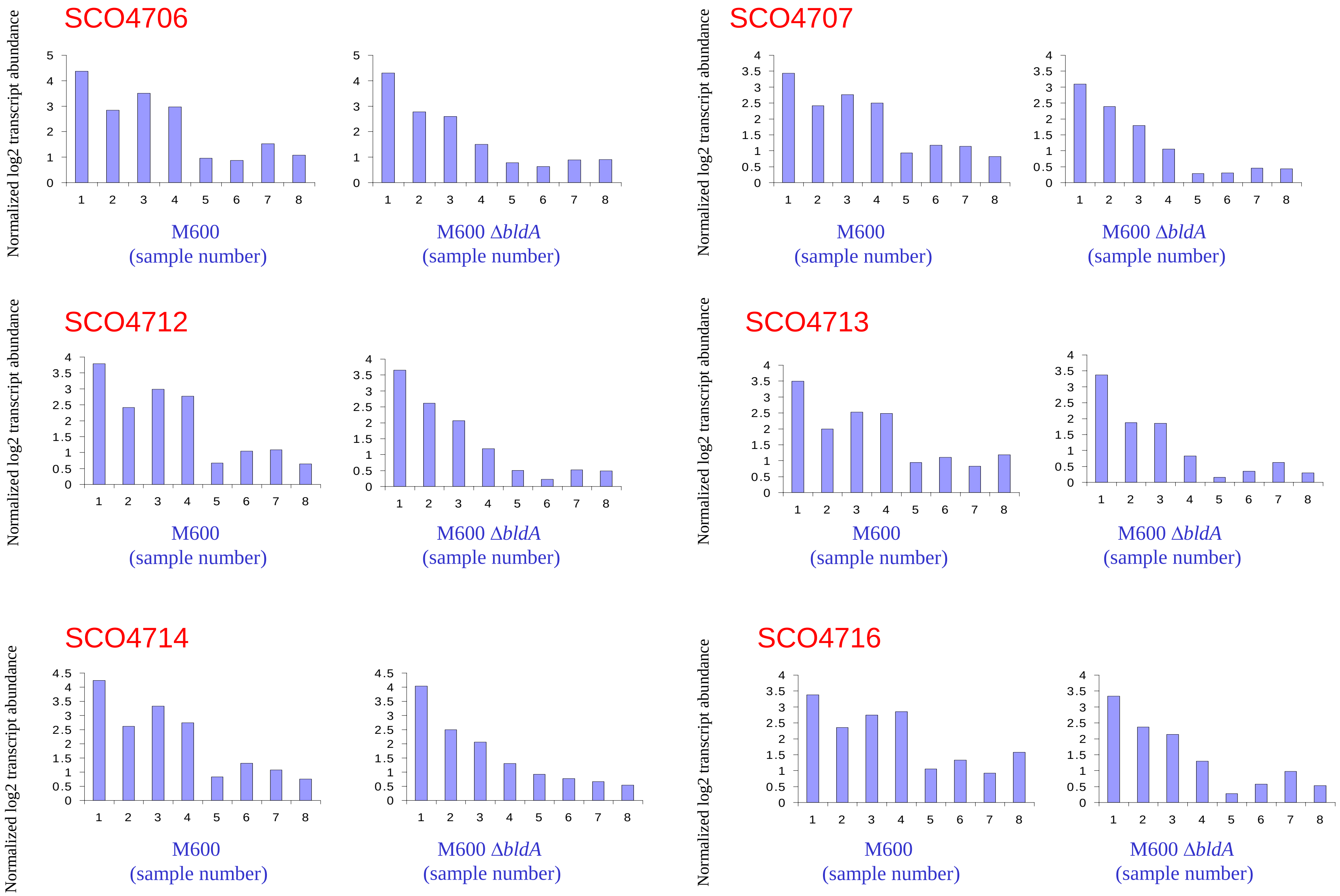


Profiles are the average of triplicate experiments

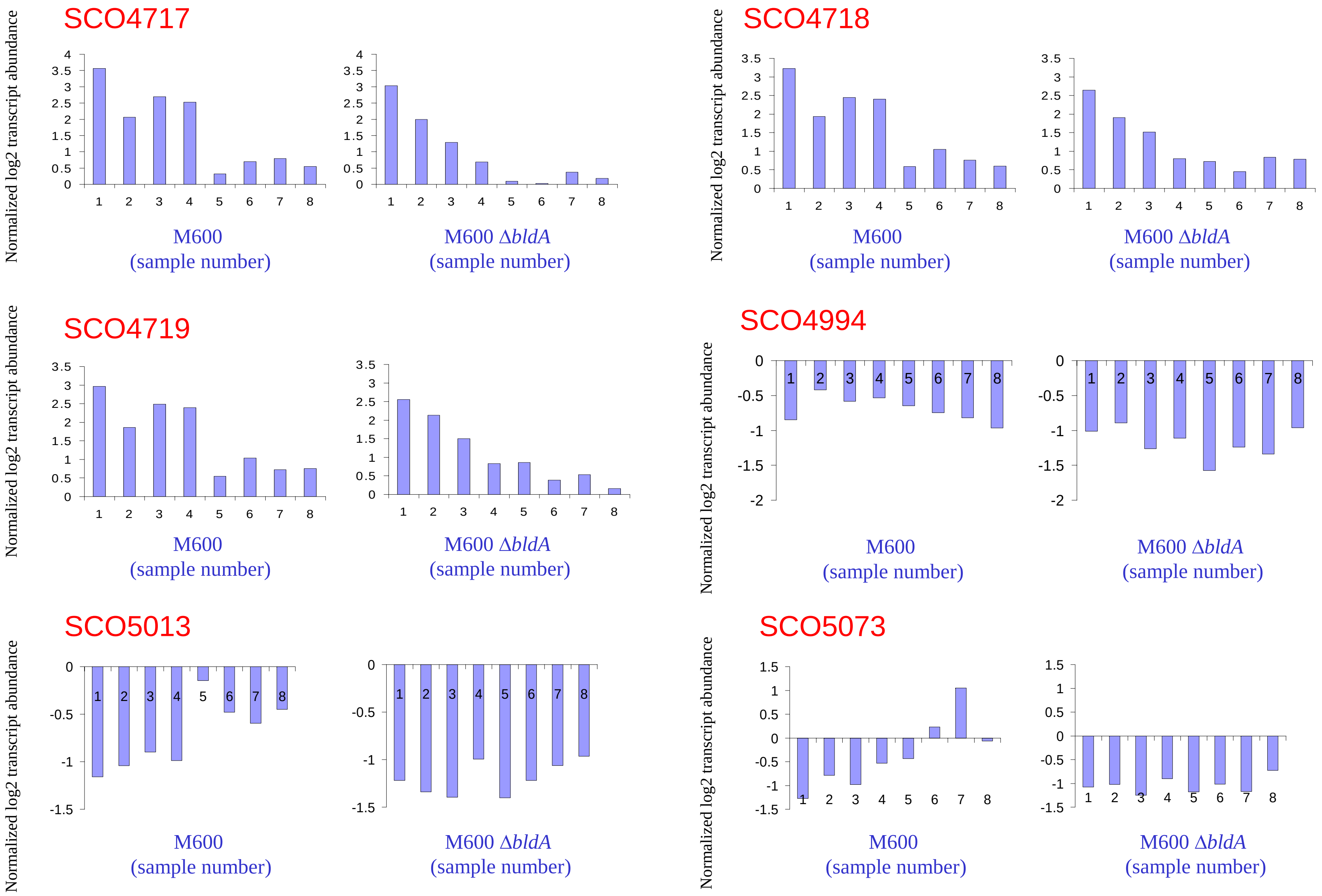


Profiles are the average of triplicate experiments

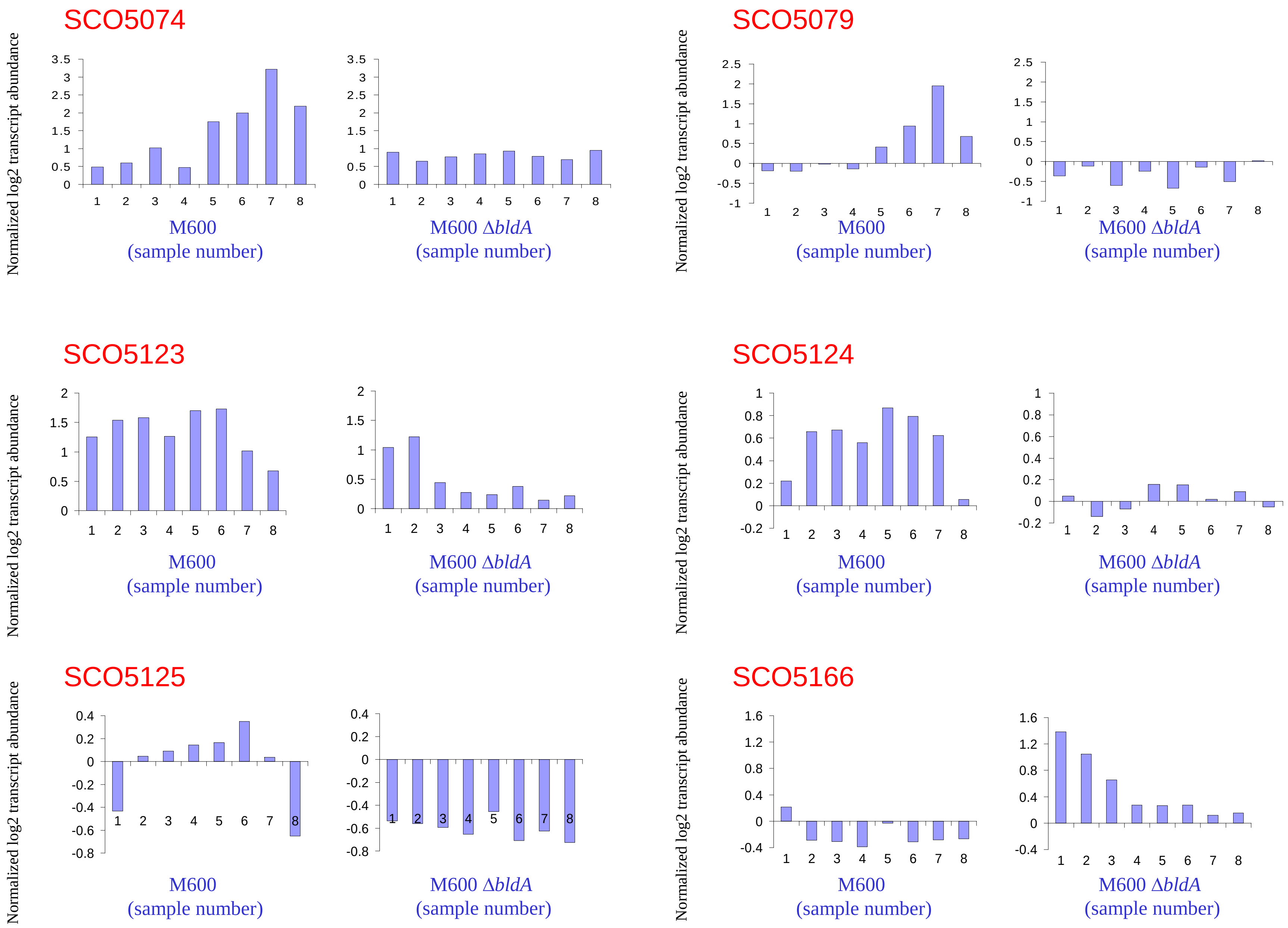


Profiles are the average of triplicate experiments

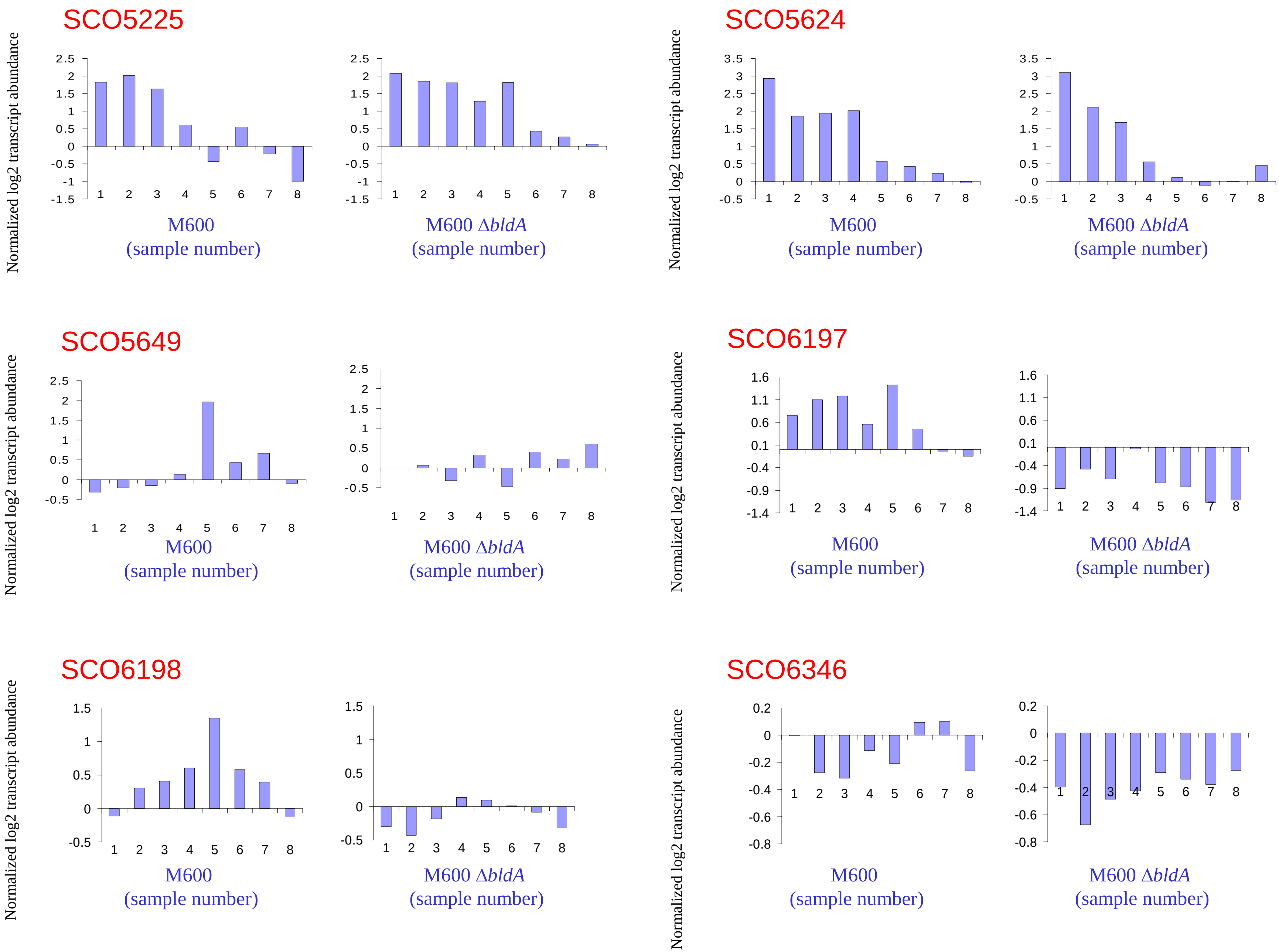


Profiles are the average of triplicate experiments

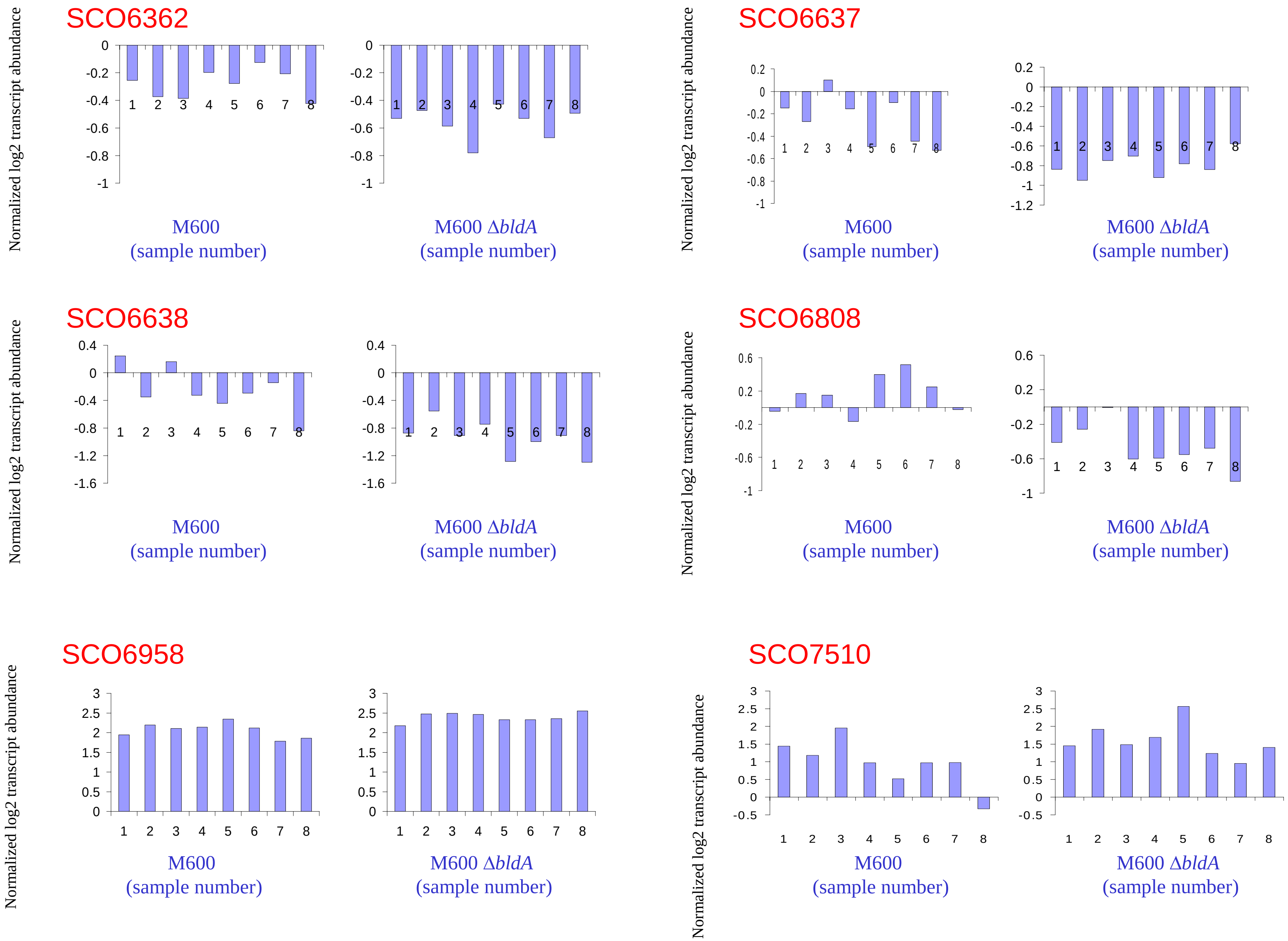


Profiles are the average of triplicate experiments

SCO7511

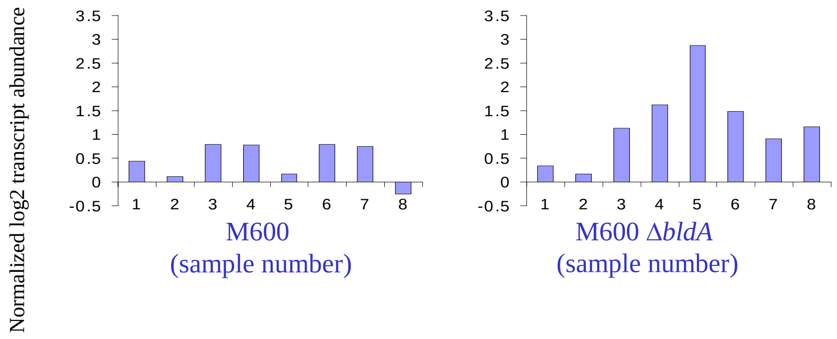


SCO7657

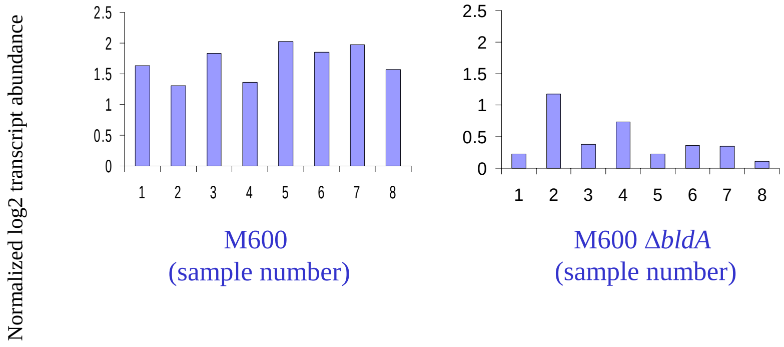


Profiles are the average of triplicate experiments
