# Supplementary material for: New pleiotropic effects of eliminating a rare tRNA from Streptomyces coelicolor, revealed by combined proteomic and transcriptomic analysis of liquid cultures
Source: BMC Genomics. 2007 Aug 2;8:261. doi: 10.1186/1471-2164-8-261 (PMC2000904; doi:10.1186/1471-2164-8-261)
Supplement: Additional file 2 — Transcription profiles determined for SCO4295, SCO5013, SCO6808, SCO7657, SCO 3088, SCO 3285, SCO 3286, SCO 5166, SCO 6958, SCO 6638, SCO 4660, SCO 4702 and SCO 4648 using either a) Q-RT-PCR, or B) DNAmicroarrays. A comparison for a subset of genes of transcript abundance profiles determined using DNA microarrays with results obtained using quantitative RT-PCR. [file 1471-2164-8-261-S2.pdf]

Additional File 2.
Comparison of transcription profiles determined for SCO4295, SCO5013, SCO6808, SCO7657, SCO3088, SCO3285, SCO3286,SCO5166, SCO6958, SCO6638, SCO4660, SCO4702 and SCO4648 of replicate 3 using either
a) Q-RT-PCR, or B) DNA microarrays.

1) Expression of SCO4295

a) Q-RT-PCR

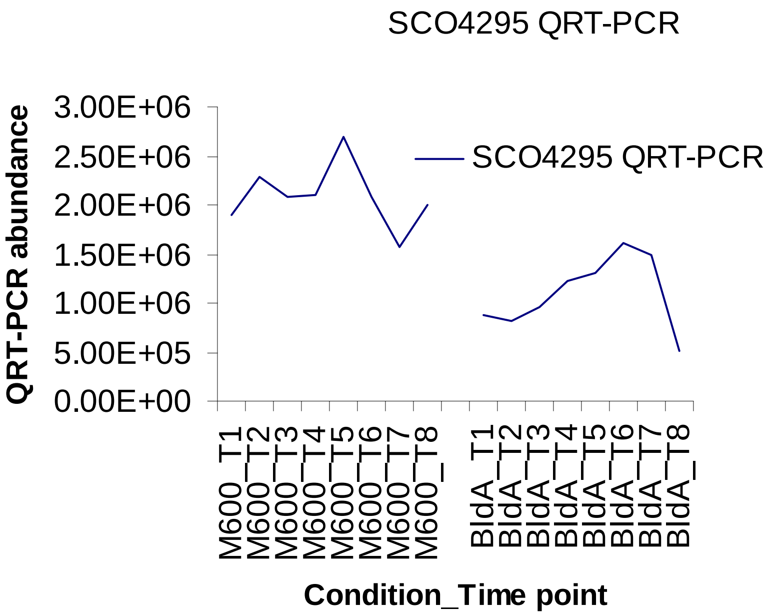

b) DNA microarrays

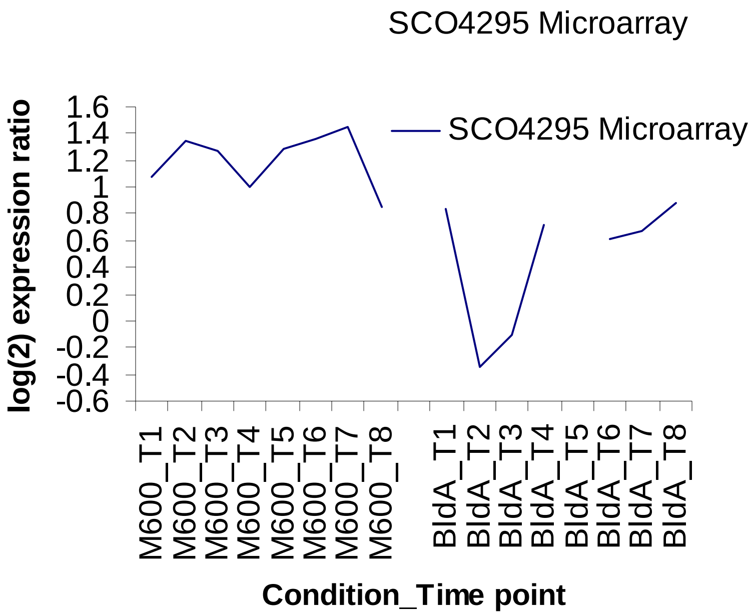

Spearman Correlation coefficient of 0.64 between the two profiles

All 16 RNA samples from one of the three time-course experiments (set C) were analysed.

2) Expression of SCO5013

a) Q-RT-PCR

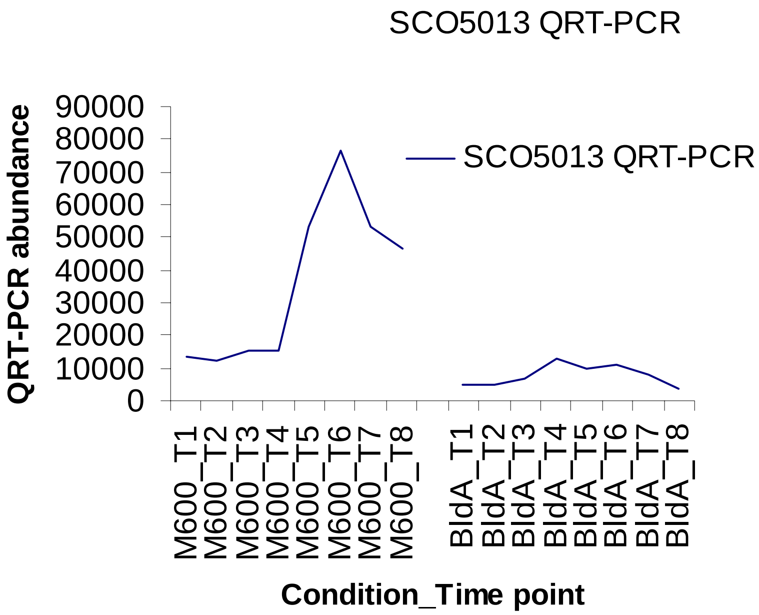

b) DNA microarrays

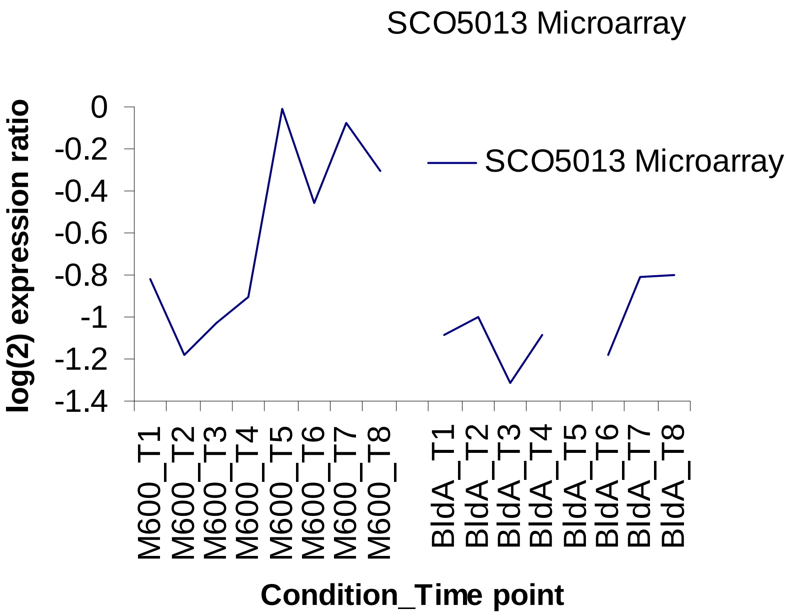

Spearman Correlation coefficient of 0.55 between the two profiles

All 16 RNA samples from one of the three time-course experiments (set C) were analysed.

3) Expression of SCO6808

a) Q-RT-PCR

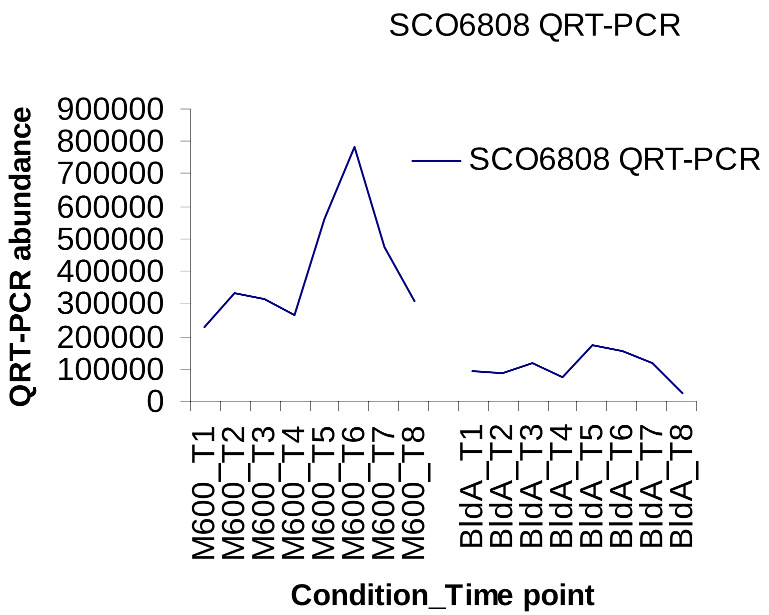

b) DNA microarrays

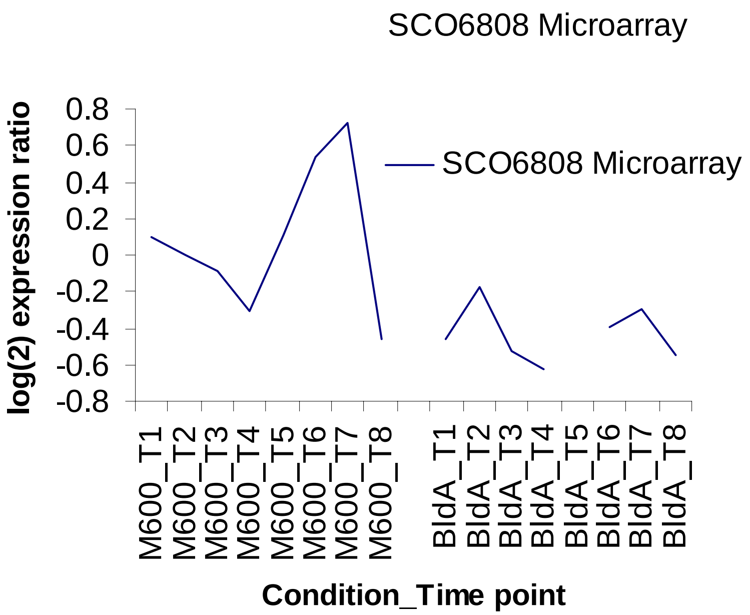

Spearman Correlation coefficient of 0.78 between the two profiles

All 16 RNA samples from one of the three time-course experiments (set C) were analysed.

4) Expression of SCO7657

a) Q-RT-PCR

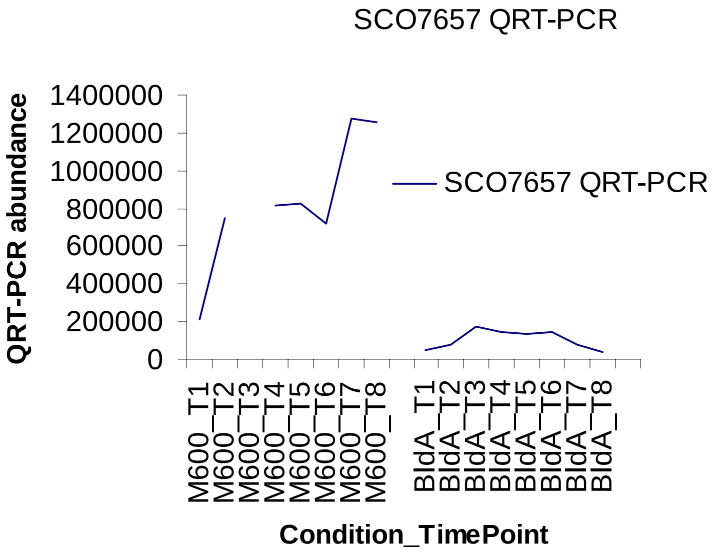

b) DNA microarrays

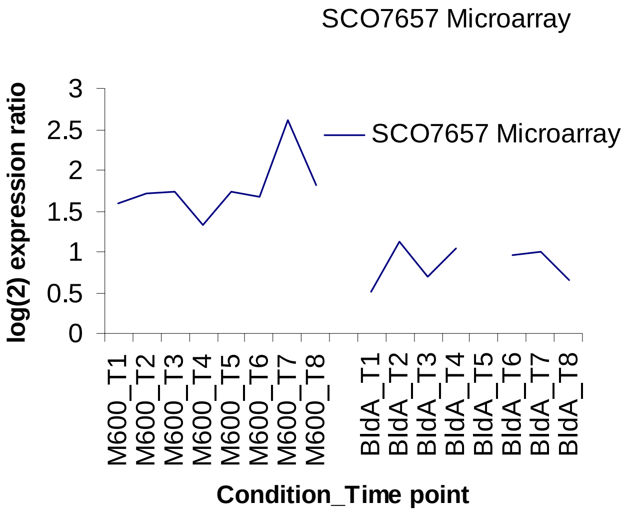

Spearman Correlation coefficient of 0.89 between the two profiles

All 16 RNA samples from one of the three time-course experiments (set C) were analysed.

Expression of SCO 3088

a) Q-RT-PCR

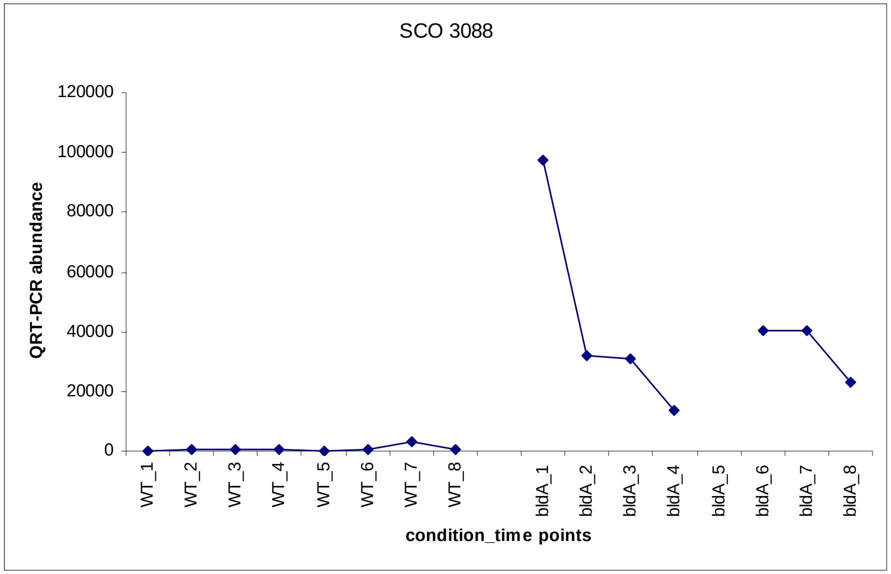

b) DNA microarray

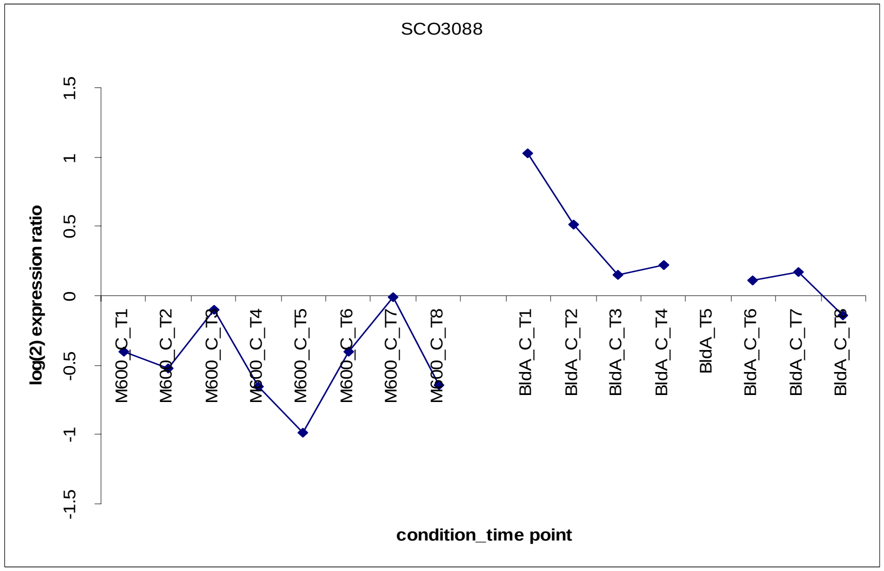

Spearman Correlation coefficient of 0.86 between the two profiles

All 16 RNA samples from one of the three time-course experiments (set C) were analysed.

Expression of SCO 3286

a) Q-RT-PCR

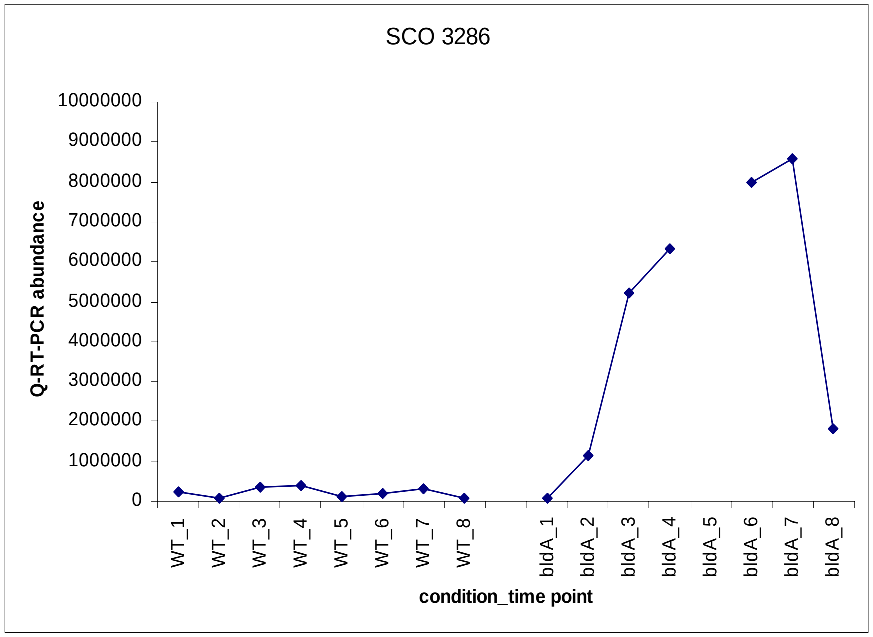

b) DNA microarray

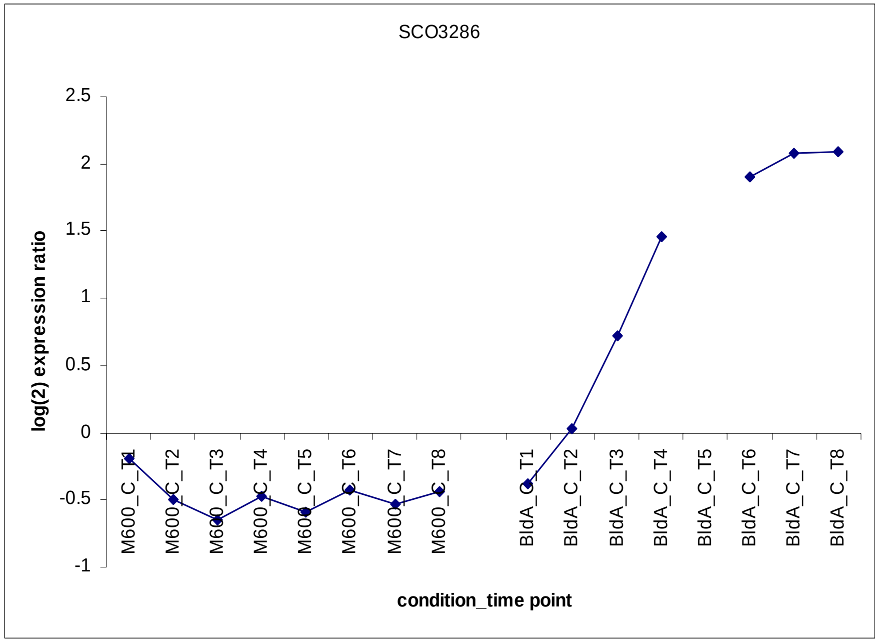

Spearman Correlation coefficient of 0.67 between the two profiles

All 16 RNA samples from one of the three time-course experiments (set C) were analysed.

Expression of SCO 4648

a) Q-RT-PCR

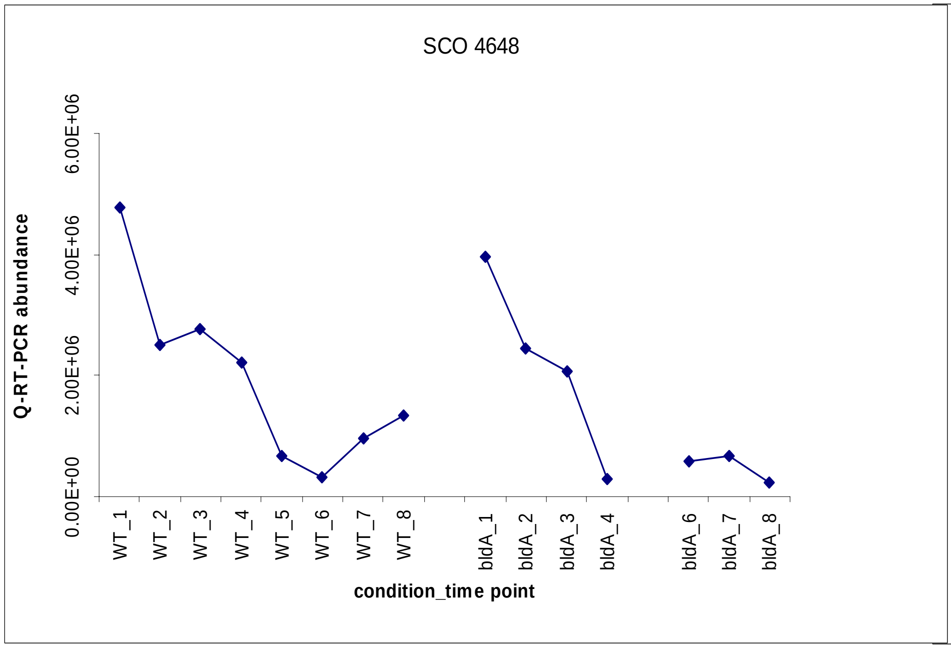

b) DNA microarray

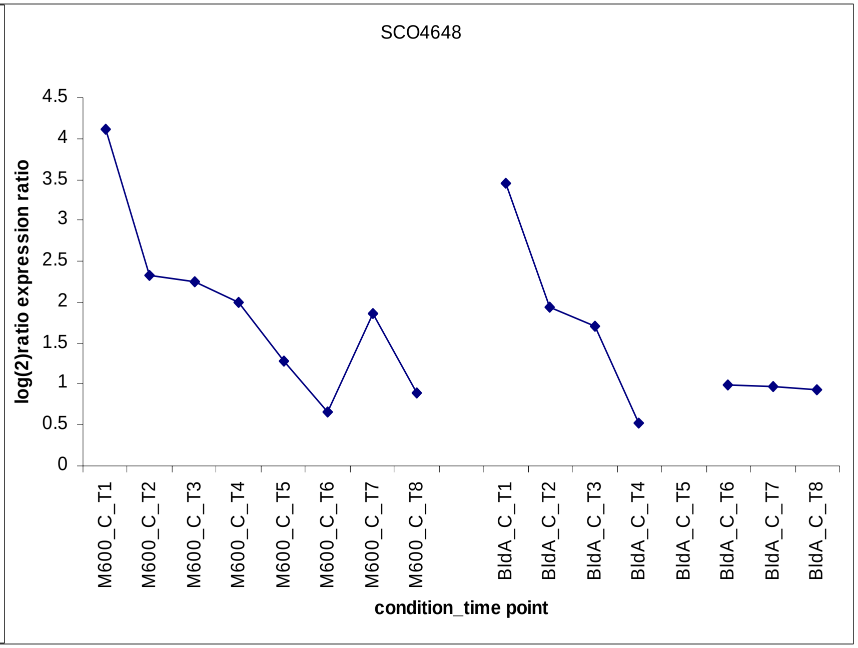

Spearman Correlation coefficient of 0.91 between the two profiles

All 16 RNA samples from one of the three time-course experiments (set C) were analysed.

Expression of SCO 4660

a) Q-RT-PCR

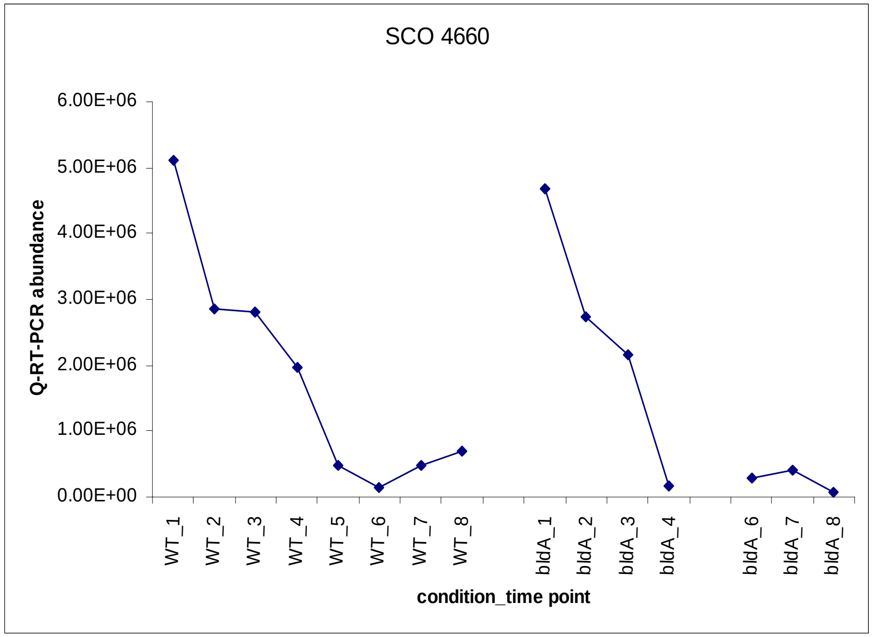

b) DNA microarray

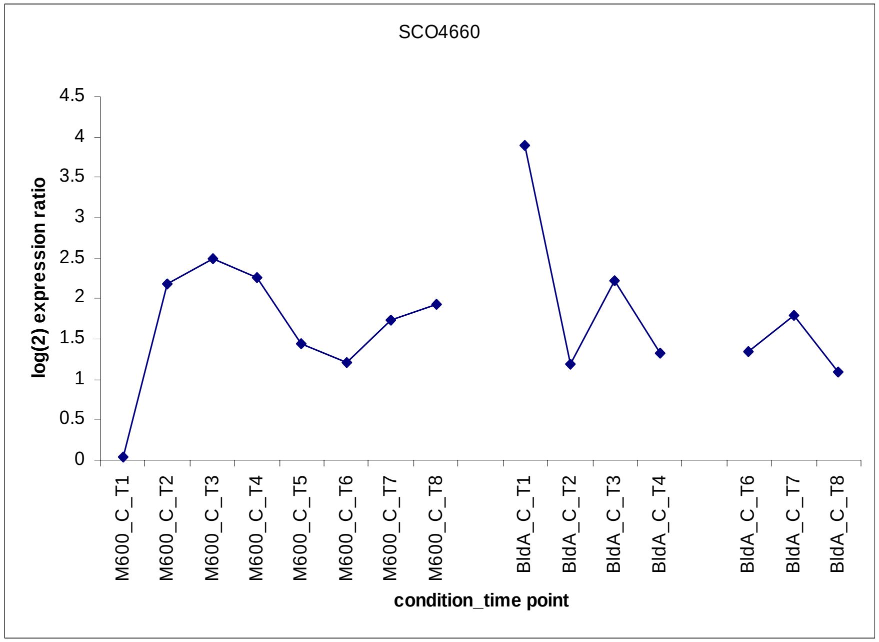

Spearman Correlation coefficient of 0.42 between the two profiles

All 16 RNA samples from one of the three time-course experiments (set C) were analysed.

Expression of SCO 4702

a) Q-RT-PCR

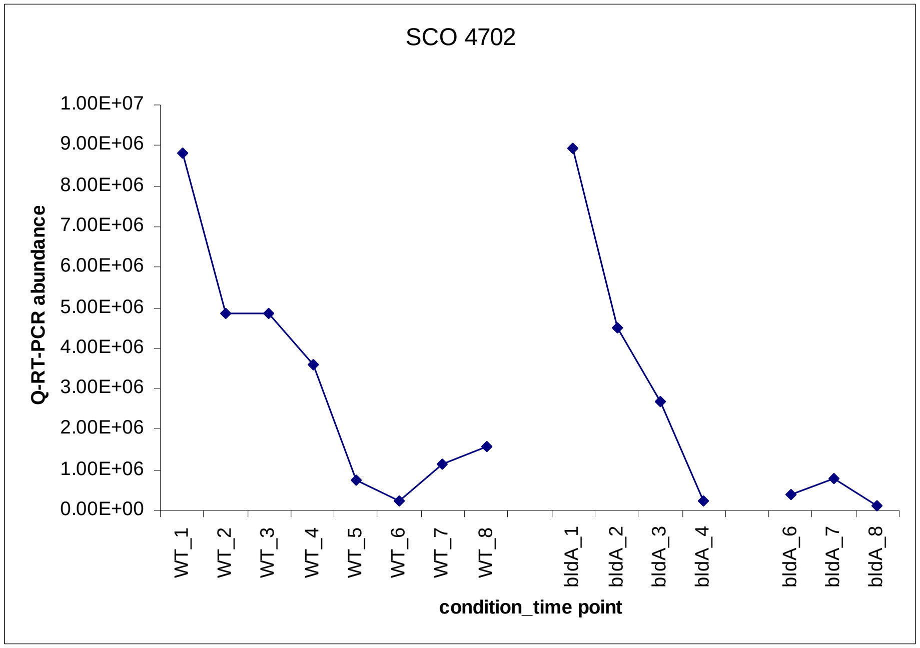

b) DNA microarray

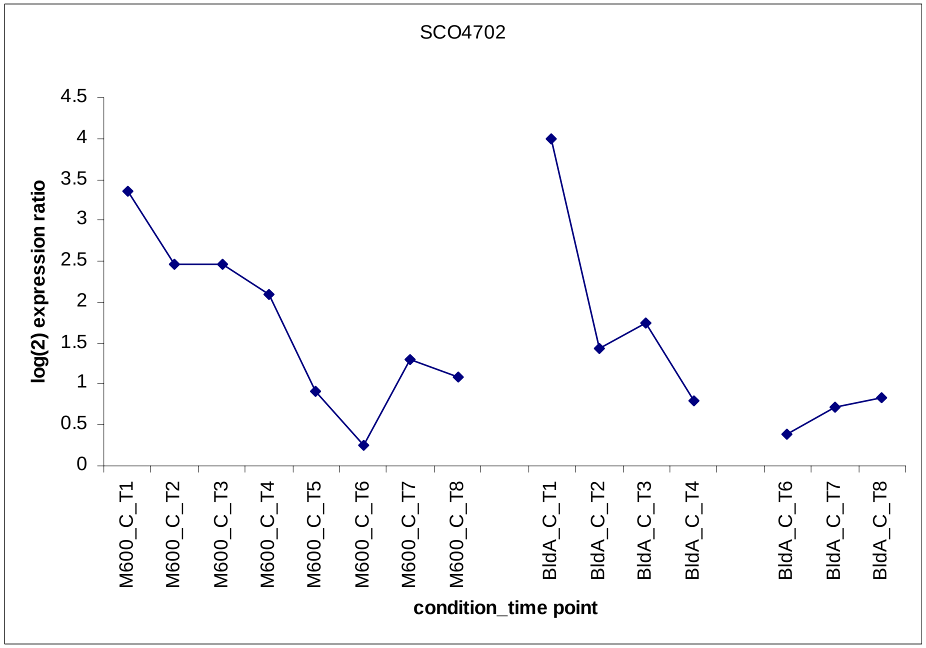

Spearman Correlation coefficient of 0.928 between the two profiles

All 16 RNA samples from one of the three time-course experiments (set C) were analysed.

Expression of SCO 4717

a) Q-RT-PCR

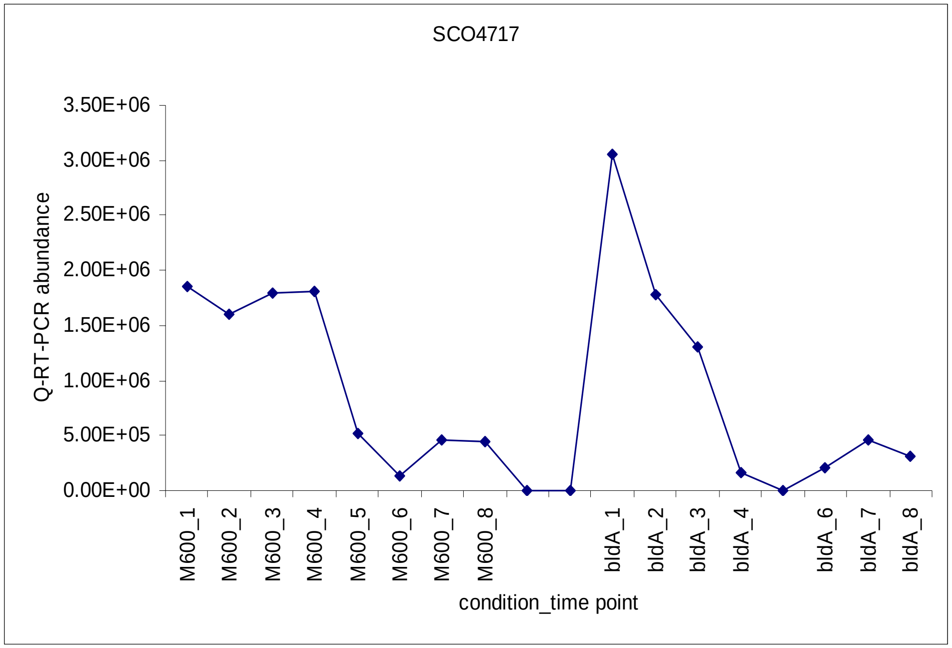

b) DNA microarray

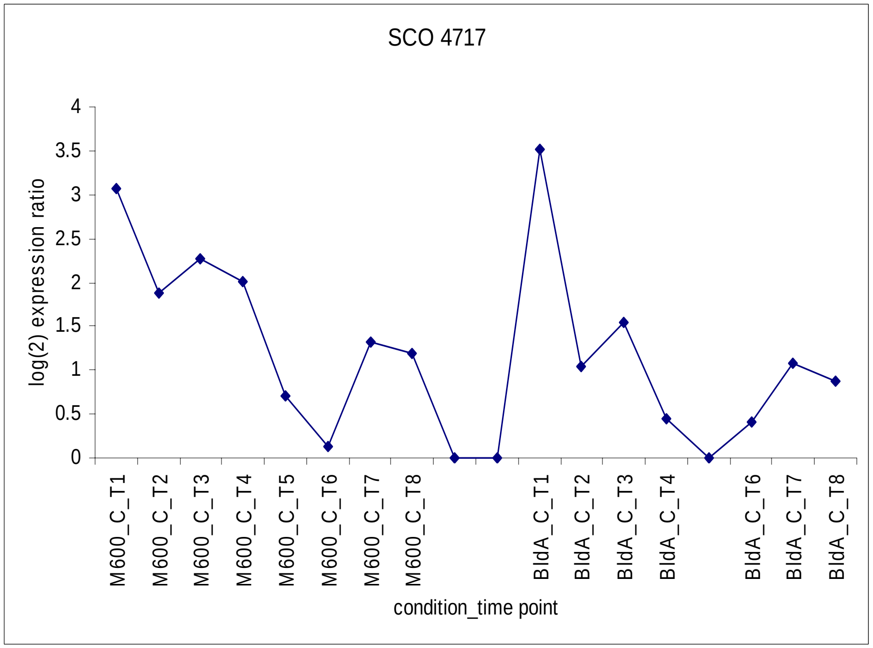

Spearman Correlation coefficient of 0.88 between the two profiles

All 16 RNA samples from one of the three time-course experiments (set C) were analysed.

Expression of SCO 5166

a) Q-RT-PCR

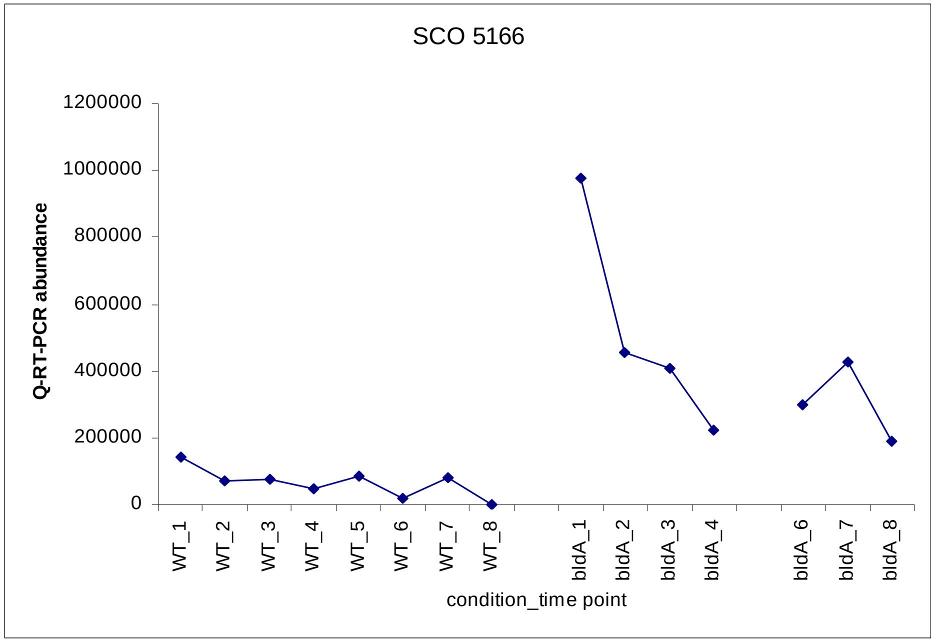

b) DNA microarray

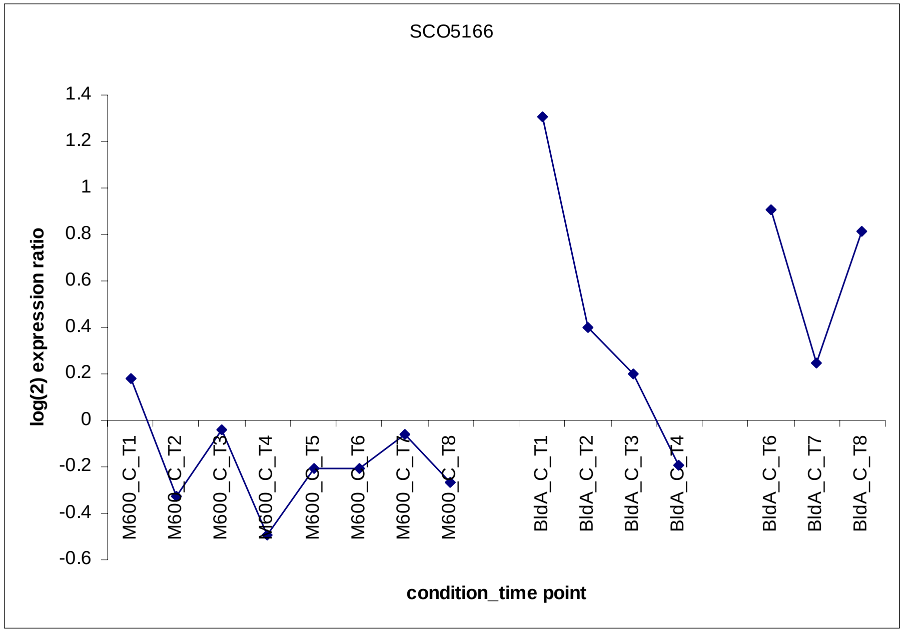

Spearman Correlation coefficient of 0.83 between the two profiles

All 16 RNA samples from one of the three time-course experiments (set C) were analysed.

Expression of SCO 6638

a) Q-RT-PCR

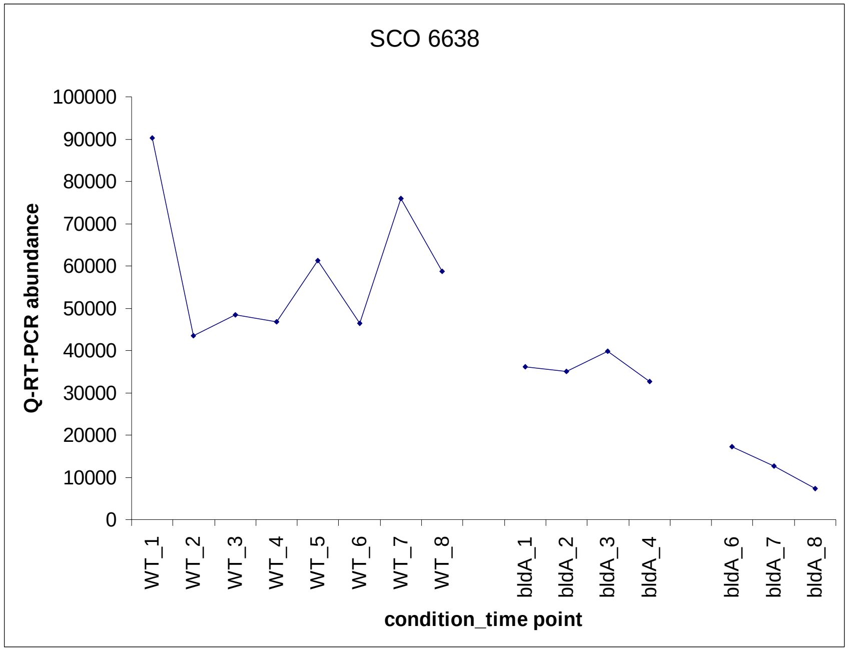

b) DNA microarray

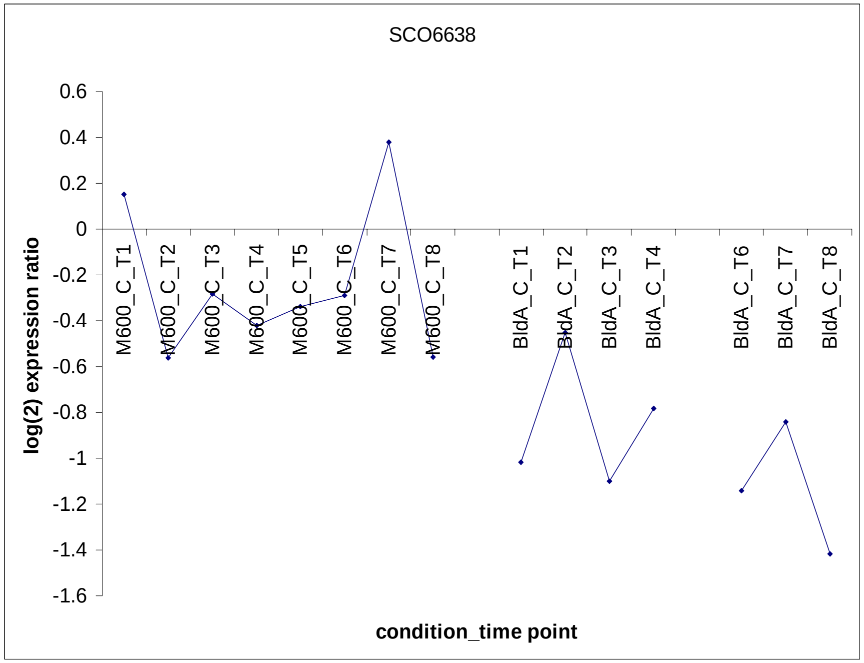

Spearman Correlation coefficient of 0.846 between the two profiles

All 16 RNA samples from one of the three time-course experiments (set C) were analysed.

Expression of SCO 6958

a) Q-RT-PCR

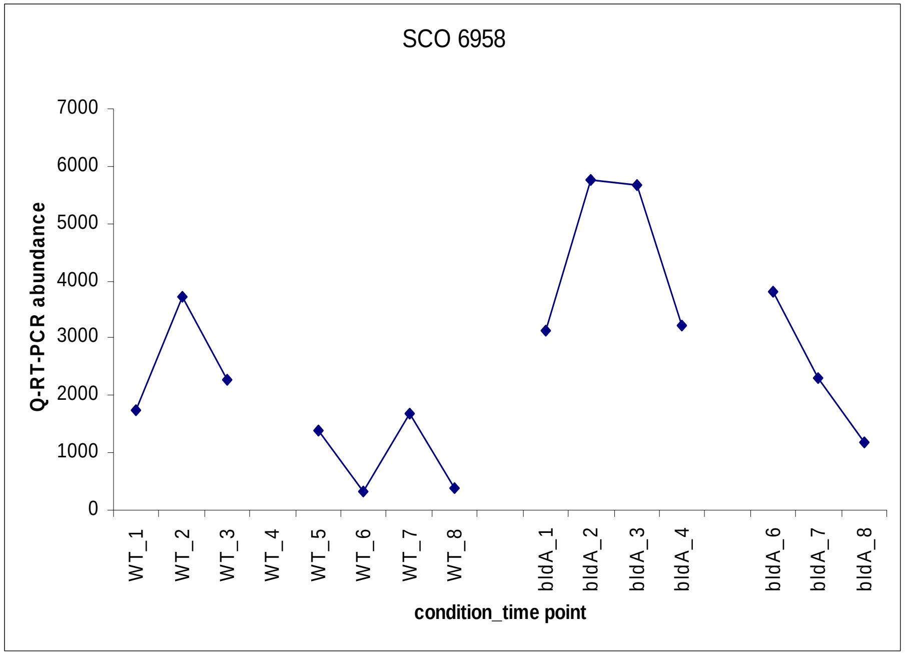

b) DNA microarray

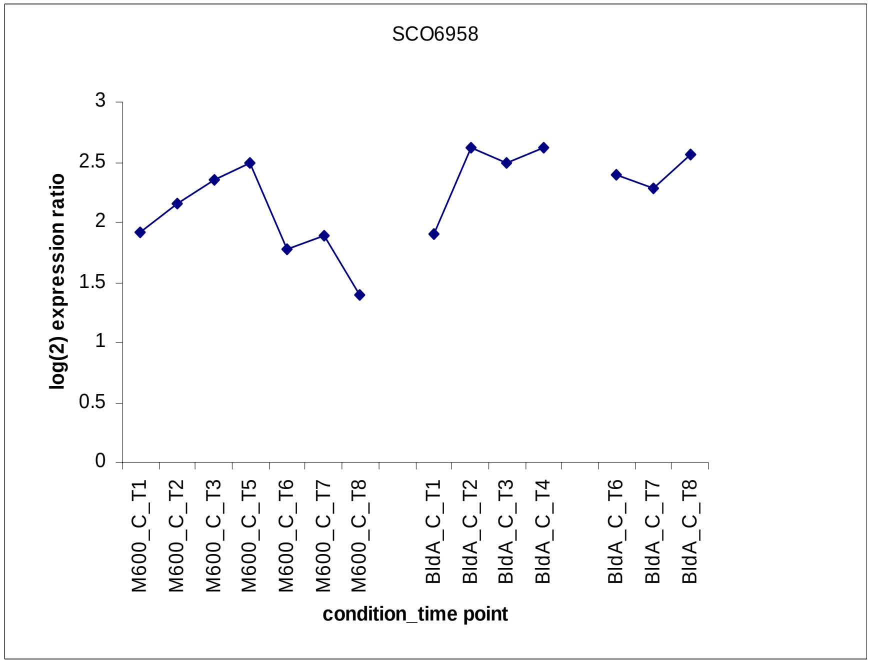

Spearman Correlation coefficient of 0.547 between the two profiles

All 16 RNA samples from one of the three time-course experiments (set C) were analysed.
In the Q-RT-PCR, WT T4 was undetected

Expression of SCO 3285

a) Q-RT-PCR

b) DNA microarray

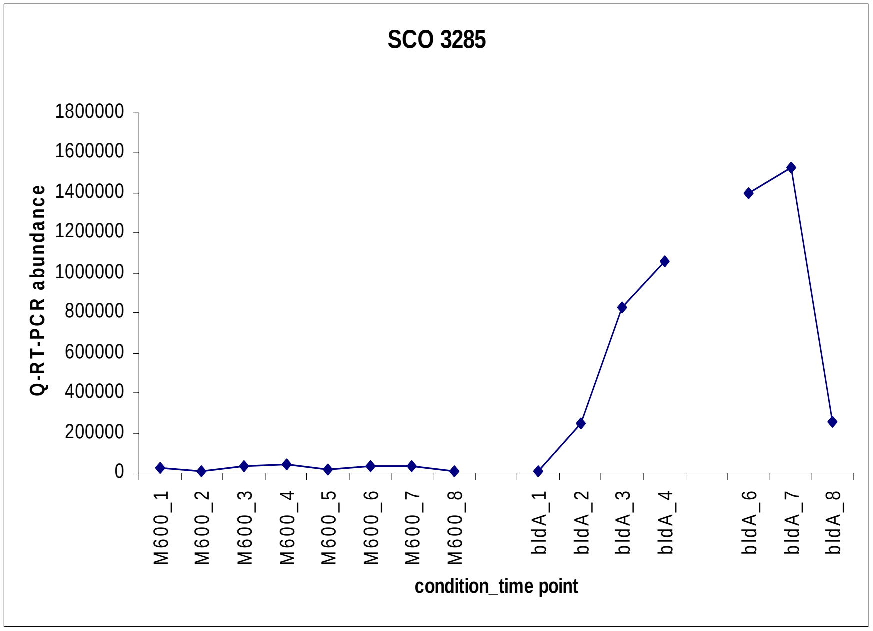

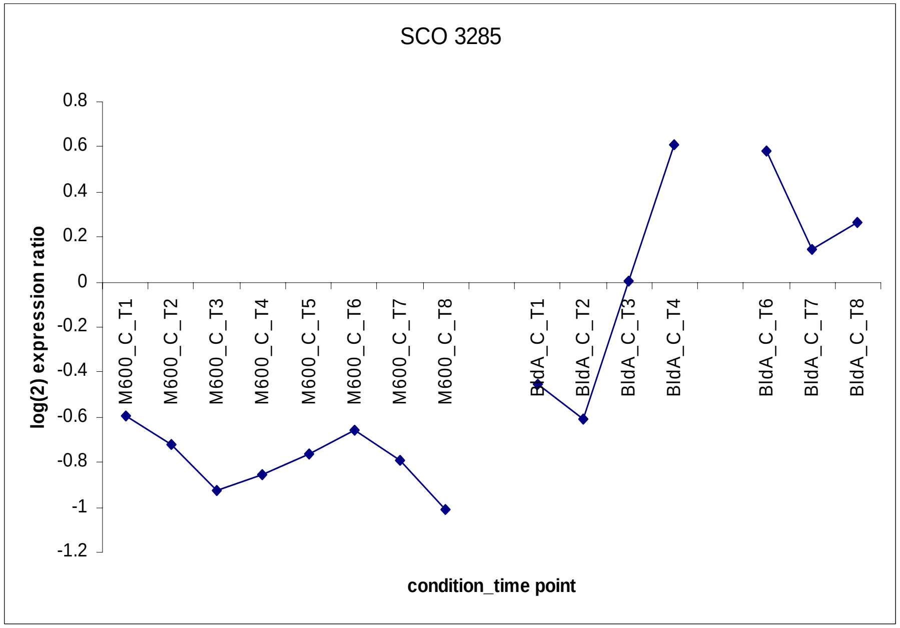

Spearman Correlation coefficient of 0.68 between the two profiles

All 16 RNA samples from one of the three time-course experiments (set C) were analysed.
