# Supplementary material for: New pleiotropic effects of eliminating a rare tRNA from Streptomyces coelicolor, revealed by combined proteomic and transcriptomic analysis of liquid cultures
Source: BMC Genomics. 2007 Aug 2;8:261. doi: 10.1186/1471-2164-8-261 (PMC2000904; doi:10.1186/1471-2164-8-261)
Supplement: Additional file 3 — A list of ribosomal protein genes present in the S. coelicolor genome, and the predicted physical properties of their gene products. Illustrates that the vast majority of ribosomal proteins would not be expected to be detected in the protomics analysis undertaken in this study. [file 1471-2164-8-261-S3.doc]

Additional file 3. A list of ribosomal protein genes present in the *S. coelicolor* genome, and the predicted physical properties of their gene products. Those with SCO numbers given in bold would not be expected to be detected on the 2D gels in the proteomics analysis because of their isoelectric point (pI) and/or molecular weight (Mwt).

| Gene | Gene product | pI^1^ | Mwt^1^ (Da) |
| --- | --- | --- | --- |
| SCO0436 | probable 50S ribosomal protein | 12.2459 | 6498 |
| SCO0569 | putative 50S ribosomal protein L36 | 12.2299 | 4622 |
| SCO0570 | 50S ribosomal protein L33 | 10.5662 | 6152 |
| SCO1150 | 50S ribosomal protein L31 | 5.02719 | 10062 |
| SCO1505 | 30S ribosomal protein S4 | 10.152 | 23610 |
| **SCO1598, rplT** | 50S ribosomal protein L20 | 10.8679 | 14386 |
| **SCO1599, rpmI** | 50S ribosomal protein L35 | 11.6698 | 7022 |
| SCO1998, rpsA | 30S ribosomal protein S1 | 4.6432 | 55033 |
| SCO2563, rspT | 30s ribosomal protein S20. | 10.9593 | 9541 |
| **SCO2596, rpmA** | 50S ribosomal protein L27 | 11.9125 | 8773 |
| **SCO2597, rplU** | ribosomal protein L21 | 9.60193 | 11664 |
| SCO3124 | ribosomal L25p family protein | 4.80086 | 20828 |
| SCO3425, rpsR2 | putative 30S ribosomal protein S18 | 11.5538 | 9323 |
| **SCO3427, rpmE** | putative 50S ribosomal protein L31 | 8.55347 | 9780 |
| **SCO3428, rpmG** | putative 50S ribosomal protein L33 | 10.8555 | 6416 |
| **SCO3429, rpmB** | putative 50S ribosomal protein L28 | 12.1784 | 8406 |
| **SCO3430, rpsN** | putative 30S ribosomal protein S14 | 11.7131 | 11768 |
| **SCO3880, rpmH** | putative 50S ribosomal protein L34 | 13.0806 | 5296 |
| **SCO3906, rpsF** | putative 30S ribosomal protein S6 | 6.56592 | 11176 |
| SCO3908, rpsR | putative 30S ribosomal protein S18 | 10.3733 | 8989 |
| **SCO3909, rplI** | 50S ribosomal protein L9 | 9.59894 | 15968 |
| **SCO4635, rpmG3** | 50S ribosomal protein L33 | 9.901 | 6406 |
| **SCO4648, rplK** | 50S ribosomal protein L11 | 9.50323 | 15393 |
| **SCO4649, rlpA** | 50S ribosomal protein L1 | 9.52032 | 25763 |
| **SCO4652, rplJ** | 50S ribosomal protein L10 | 8.82434 | 18646 |
| SCO4653, rplL | 50S ribosomal protein L7/L12 | 4.62408 | 13208 |
| SCO4659, rspL | 30S ribosomal protein S12 | 11.1934 | 13770 |
| **SCO4660, rspG** | 30S ribosomal protein S7 | 10.4024 | 17431 |
| **SCO4701, rpsJ** | 30S ribosomal protein S10 | 9.26825 | 11521 |
| **SCO4702, rplC** | 50S ribosomal protein L3 | 10.1944 | 22768 |
| **SCO4703, rplD** | 50S ribosomal protein L4 | 10.0326 | 23642 |
| **SCO4704, rplW** | 50S ribosomal protein L23 | 10.6207 | 14977 |
| **SCO4705, rplB** | 50S ribosomal protein L2 | 11.3876 | 30550 |
| **SCO4706, rpsS** | 30S ribosomal protein S19 | 10.808 | 10573 |
| **SCO4707, rplV** | 50S ribosomal protein L22 | 10.2471 | 13863 |
| **SCO4708, rpsC** | 30S ribosomal protein S3 | 10.4441 | 30273 |
| **SCO4709, rplP** | 50S ribosomal protein L16 | 11.0153 | 15843 |
| **SCO4710, rpmC** | 50S ribosomal protein L29 | 7.26147 | 8401 |
| **SCO4711, rpsQ** | 30S ribosomal protein S17 | 9.90997 | 10720 |
| SCO4712, rplN | 50S ribosomal protein L14 | 10.2616 | 13369 |
| **SCO4713, rplX** | 50S ribosomal protein L24 | 10.0488 | 11590 |
| **SCO4714, rplE** | 50S ribosomal protein L5 | 9.69037 | 20852 |
| **SCO4715, rpsN** | 30S ribosomal protein S14 | 11.0042 | 6949 |
| **SCO4716, rpsH** | 30S ribosomal protein S8 | 9.69785 | 14276 |
| **SCO4717, rplF** | 50S ribosomal protein L6 | 9.84984 | 19178 |
| **SCO4718, rplR** | 50S ribosomal protein L18 | 10.6128 | 13578 |
| **SCO4719, rpsE** | 30S ribosomal protein S5 | 10.1947 | 20499 |
| **SCO4720, rpmD** | 50S ribosomal protein L30 | 9.69763 | 6887 |
| **SCO4721, rplO,** | 50S ribosomal protein L15 | 10.1292 | 15947 |
| **SCO4726, rpmJ** | 50S ribosomal protein L36 | 11.0042 | 4401 |
| **SCO4727, rpsM** | 30S ribosomal protein S13 | 11.1966 | 14219 |
| **SCO4728, rpsK** | 30S ribosomal protein S11 | 11.5386 | 14398 |
| **SCO4730, rplQ** | 50S ribosomal protein L17 | 9.42676 | 18130 |
| **SCO4734, rplM** | 50S ribosomal protein L13 | 9.90784 | 16447 |
| **SCO4735, rpsI** | 30S ribosomal protein S9 | 9.25159 | 18698 |
| **SCO5359, rpmE3** | 50S ribosomal protein L31 | 8.80127 | 8101 |
| **SCO5564** | putative 50S ribosomal protein L28 | 11.5549 | 6638 |
| **SCO5571** | 50S ribosomal protein L32 | 10.2812 | 6572 |
| **SCO5591, rpsP** | 30S ribosomal protein S16 | 9.25351 | 15196 |
| **SCO5595, rplS** | 50S ribosomal protein L19 | 10.8879 | 13141 |
| SCO5624, rpsB | 30S ribosomal protein S2 | 5.18559 | 33591 |
| SCO5736, rpsO | 30S ribosomal protein S15 | 11.1319 | 10790 |

^1^ from ScoDB [53].

Although not detected in this study, all 4 ‘detectable’ ribosomal proteins (SCO1998, SCO3124, SCO4653 and SCO5624) have been detected on similar gels from Hesketh *et al*. [18]. They were presumably not selected for identification in this study because of unchanging abundance profiles.
