# Supplementary material for: New pleiotropic effects of eliminating a rare tRNA from Streptomyces coelicolor, revealed by combined proteomic and transcriptomic analysis of liquid cultures
Source: BMC Genomics. 2007 Aug 2;8:261. doi: 10.1186/1471-2164-8-261 (PMC2000904; doi:10.1186/1471-2164-8-261)
Supplement: Additional file 7 — Time-course abundance profiles showing changes in the post-translational processing of certain proteins as a result of bldA mutation. Illustrates the changes observed in post-translational processing of 11 proteins during growth of the wild-type and bldA mutant strains. [file 1471-2164-8-261-S7.pdf]

Additional file 7. Time-course abundance profiles showing changes in the post-translational processing of certain proteins as a result of *bldA* mutation. Two protein spots were found for each of the genes illustrated (for group A they differed in isoelectric point (pI), for group B in Mwt and pI), and one or more are altered in abundance in the *bldA* mutant. *1* and *2* indicate duplicate experiments in which cultures were sampled at five time points, times 1, 2, 4, 6 and 8 in Fig. 1. *Histogram bars* are normalised spot intensities following staining with Sypro Ruby, arranged from left to right in the same order as the samples were taken. For each protein, the bar extending to the top of the display represents the greatest abundance observed.

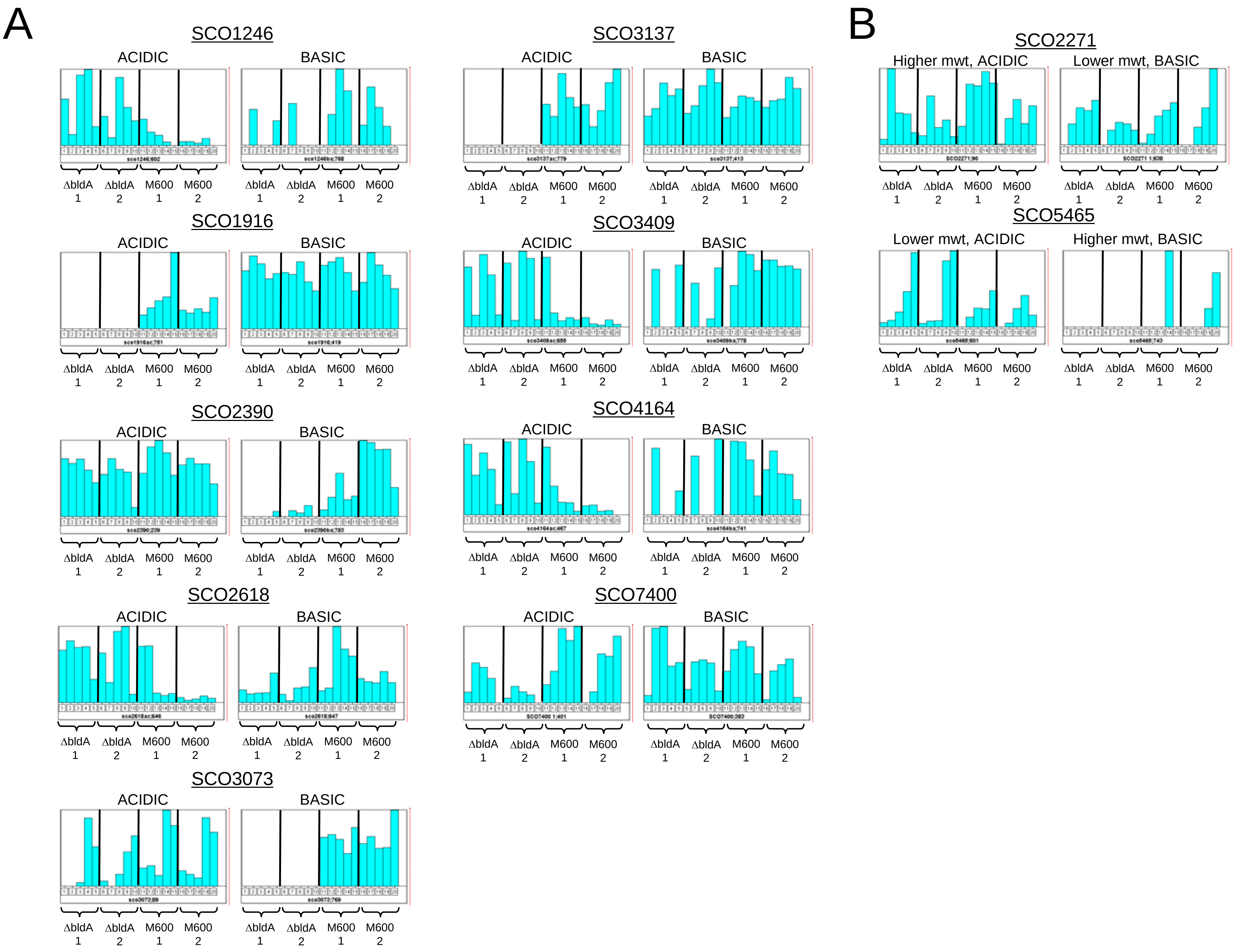
A
SCO1246
ACIDIC
BASIC
ΔbldA 1
ΔbldA 2
M600 1
M600 2
SCO1916
SCO2390
SCO2618
SCO3073
SCO3137
SCO3409
SCO4164
SCO7400
B
SCO2271
Higher mwt, ACIDIC
Lower mwt, BASIC
SCO5465
Lower mwt, ACIDIC
Higher mwt, BASIC
