# Supplementary material for: New pleiotropic effects of eliminating a rare tRNA from Streptomyces coelicolor, revealed by combined proteomic and transcriptomic analysis of liquid cultures
Source: BMC Genomics. 2007 Aug 2;8:261. doi: 10.1186/1471-2164-8-261 (PMC2000904; doi:10.1186/1471-2164-8-261)
Supplement: Additional file 9 — Comparison of transcript and protein abundance profiles for the eleven genes identified by both transcriptome and proteome analyses as being differentially expressed in the ΔbldA mutant. Compares the transcript and protein spot abundance profiles for the eleven genes identified by both transcriptome and proteome analysis as being differentially expressed in the bldA mutant. [file 1471-2164-8-261-S9.pdf]

Additional file 9.

Comparison of transcript and protein abundance profiles for the eleven genes identified by both transcriptome and proteome analyses as being differentially expressed in the ∆*bldA* mutant.

SCO0499

Transcript

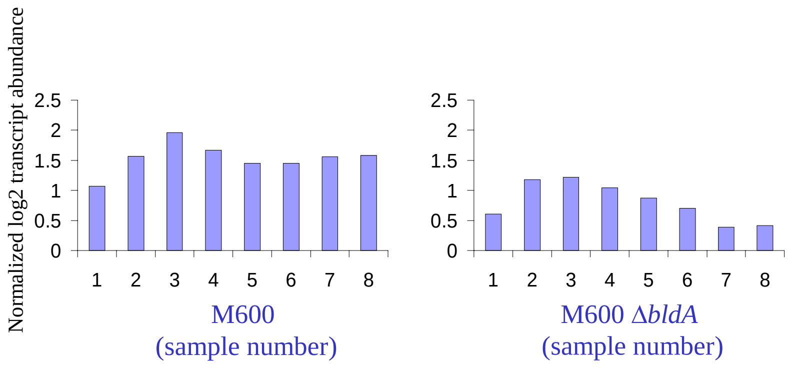

Protein

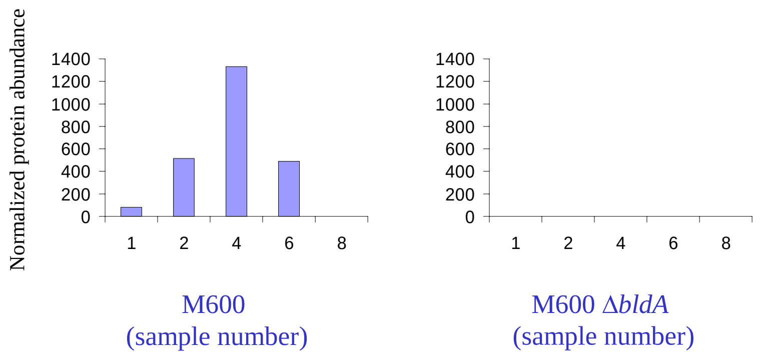

Profiles are the average of triplicate experiments for transcript abundance, and duplicate for protein abundance

SCO3285

Transcript

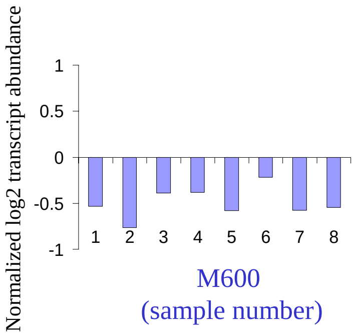

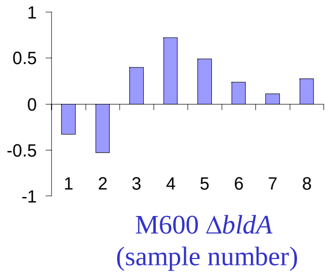

Protein

spot 1

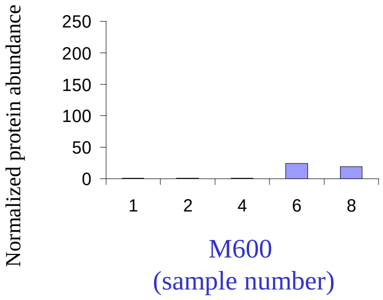

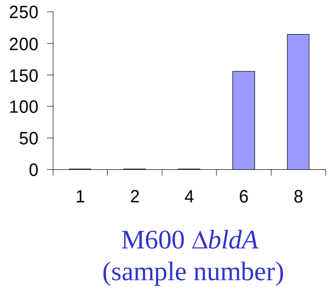

spot 2

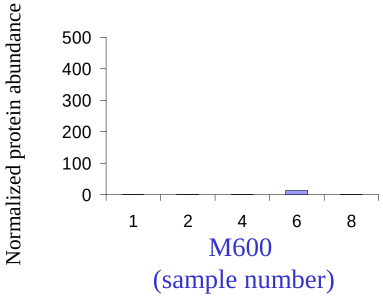

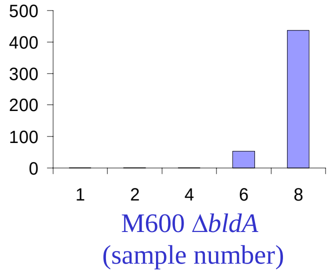

Profiles are the average of triplicate experiments for transcript abundance, and duplicate for protein abundance

SCO4252

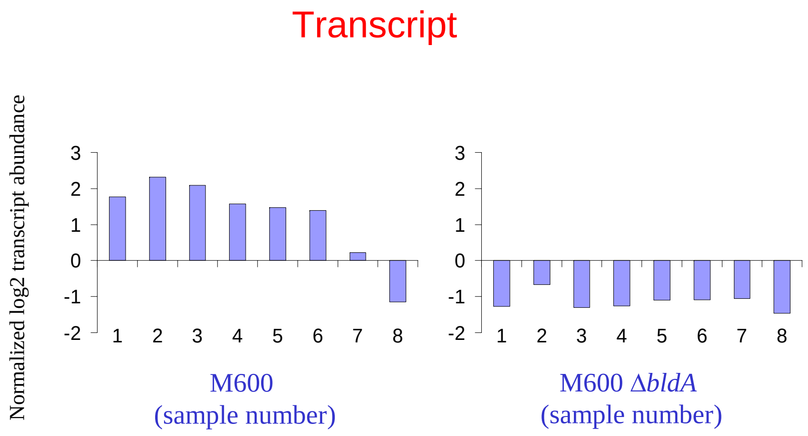

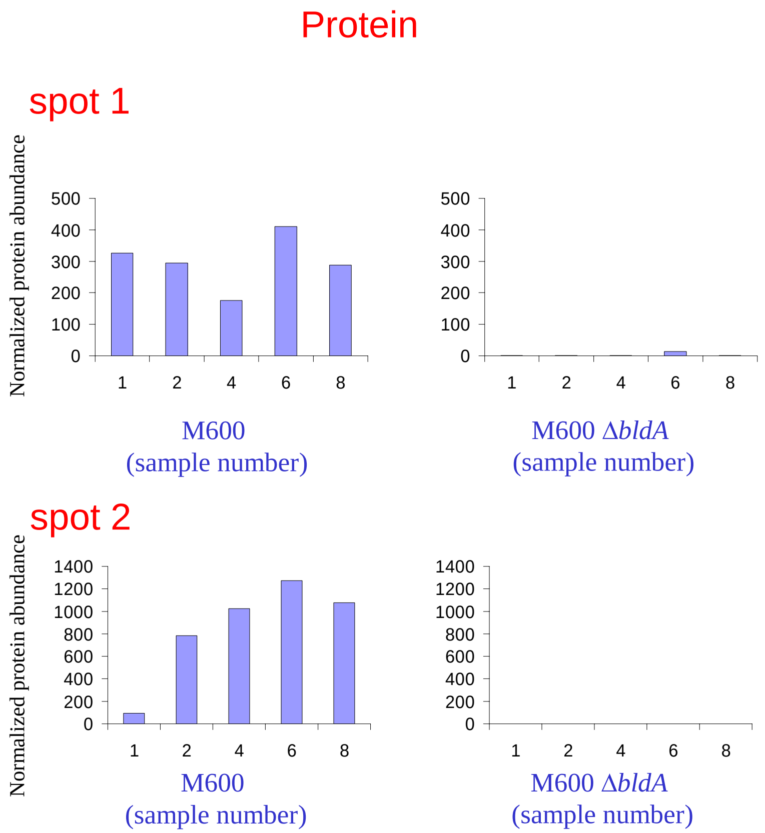

Profiles are the average of triplicate experiments for transcript abundance, and duplicate for protein abundance

SCO4253

Transcript

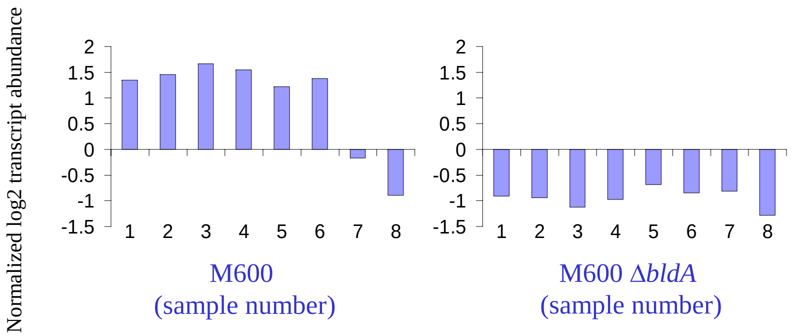

Protein

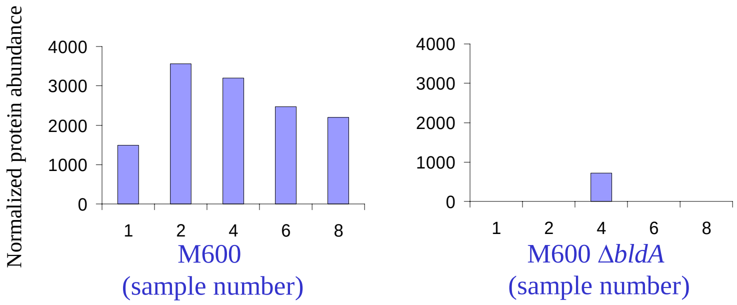

Profiles are the average of triplicate experiments for transcript abundance, and duplicate for protein abundance

SCO4677

Transcript

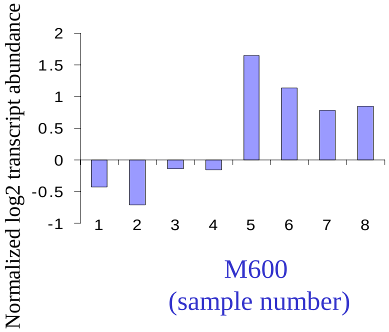

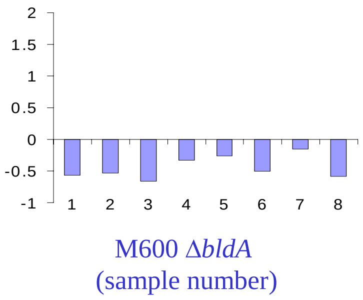

Protein

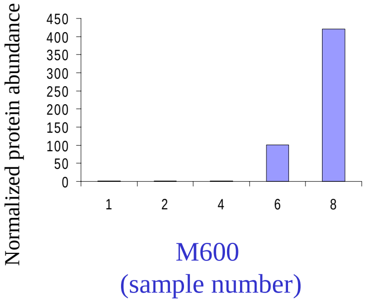

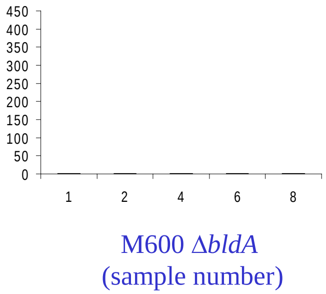

Profiles are the average of triplicate experiments for transcript abundance, and duplicate for protein abundance

SCO4994

Transcript

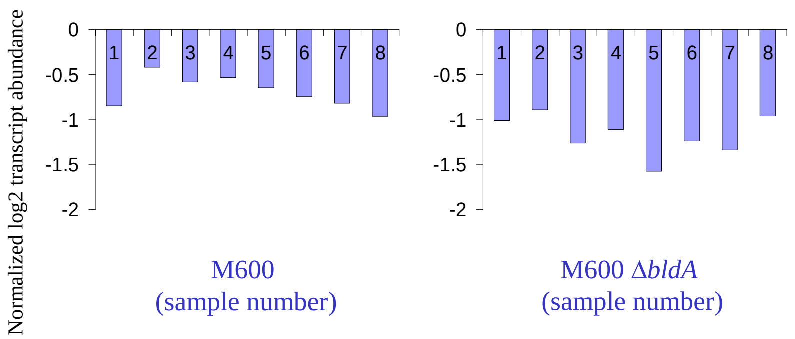

Protein

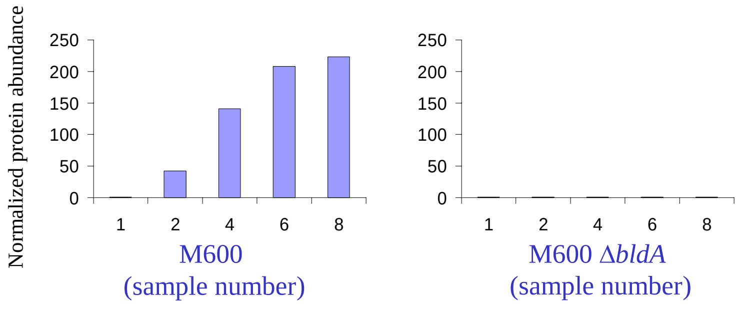

Profiles are the average of triplicate experiments for transcript abundance, and duplicate for protein abundance

SCO5073

Transcript

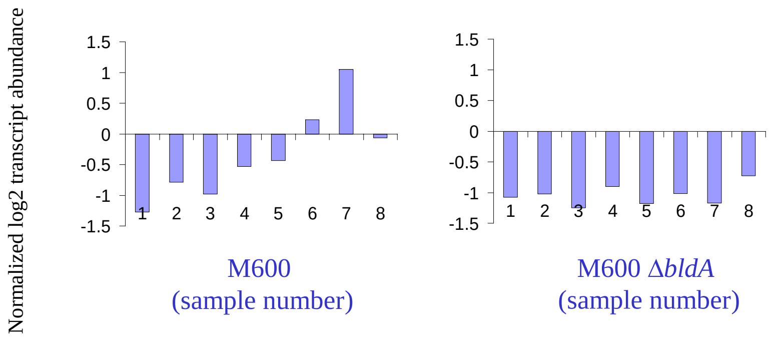

Protein

spot 1

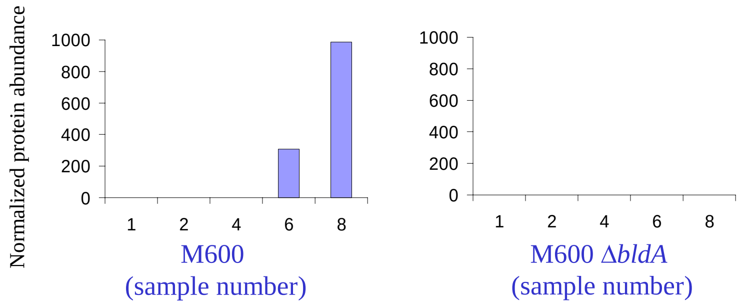

spot 2

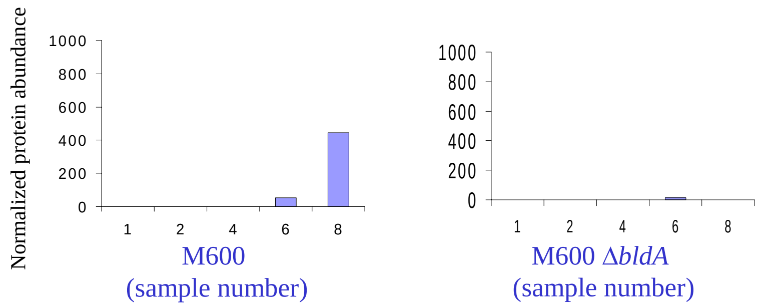

Profiles are the average of triplicate experiments for transcript abundance, and duplicate for protein abundance

SCO5079

Transcript

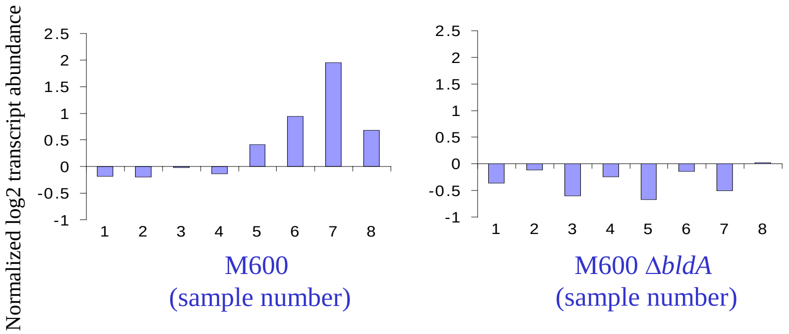

Protein

spot 1

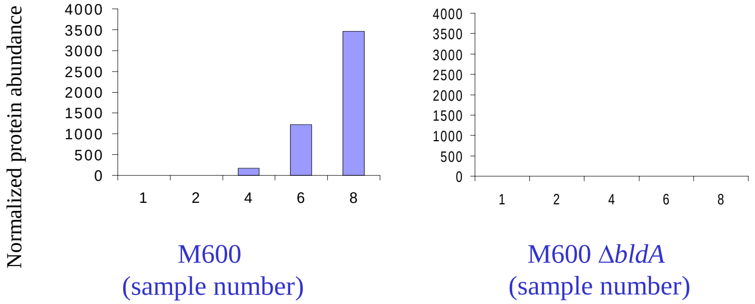

spot 2

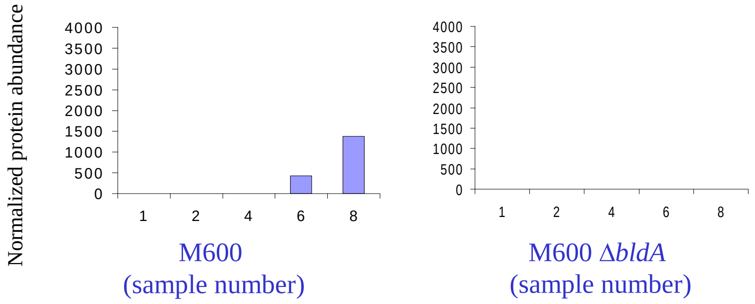

Profiles are the average of triplicate experiments for transcript abundance, and duplicate for protein abundance

SCO6637

Transcript

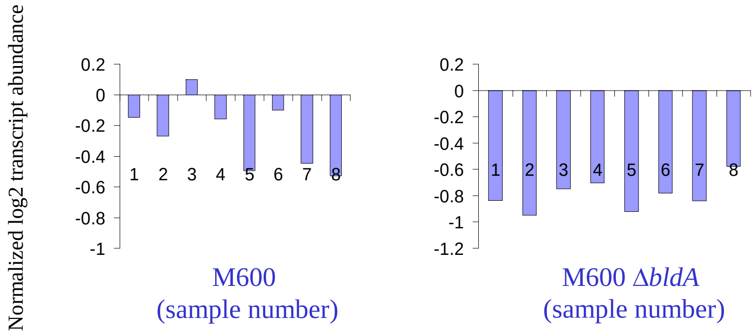

Protein

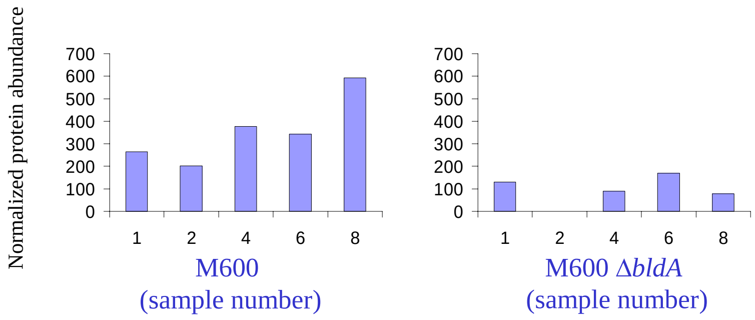

Profiles are the average of triplicate experiments for transcript abundance, and duplicate for protein abundance

SCO6638

Transcript

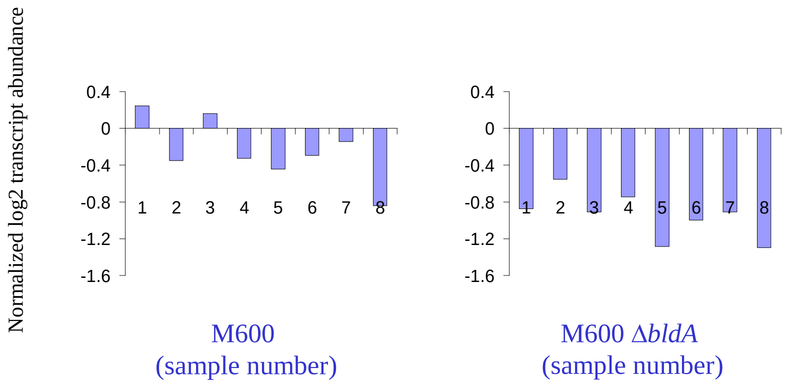

Protein

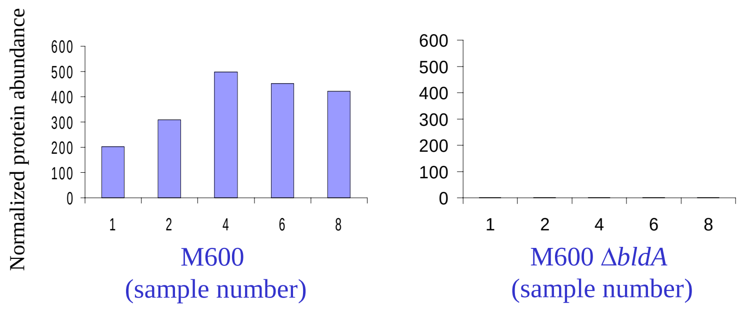

Profiles are the average of triplicate experiments for transcript abundance, and duplicate for protein abundance

SCO7511

Transcript

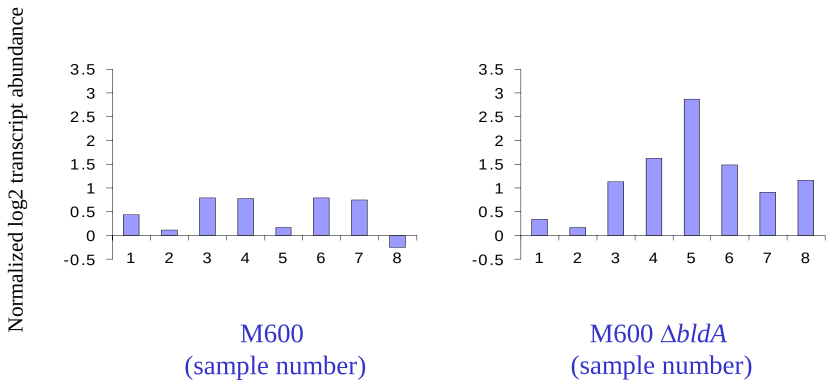

Protein

spot 1

spot 2

spot 3

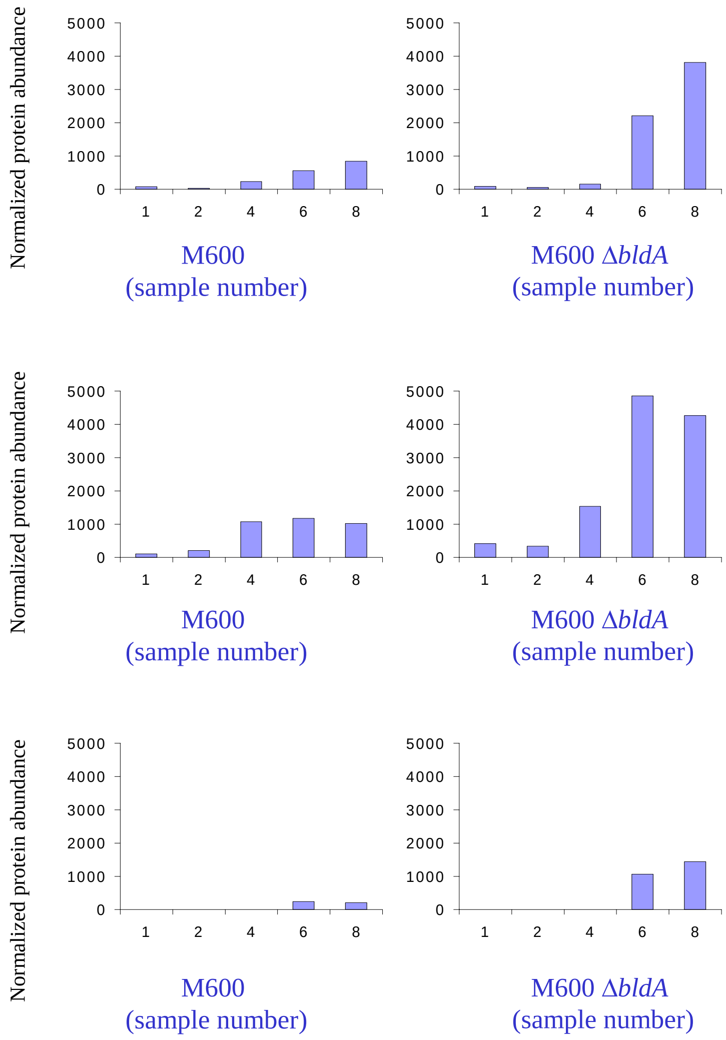

Profiles are the average of triplicate experiments for transcript abundance, and duplicate for protein abundance
