# Supplementary material for: New pleiotropic effects of eliminating a rare tRNA from Streptomyces coelicolor, revealed by combined proteomic and transcriptomic analysis of liquid cultures
Source: BMC Genomics. 2007 Aug 2;8:261. doi: 10.1186/1471-2164-8-261 (PMC2000904; doi:10.1186/1471-2164-8-261)
Supplement: Additional file 10 — Abundance data for protein spots belonging to genes responsible for the production of secondary metabolites in S. coelicolor. Illustrates differential representation of many proteins associated with secondary metabolite production as a result of bldA mutation. [file 1471-2164-8-261-S10.pdf]

Additional file 10.

Summary of the protein abundance data for protein spots belonging to genes responsible for the production of secondary metabolites in *S. coelicolor*, illustrating differential representation as a result of *bldA* mutation.

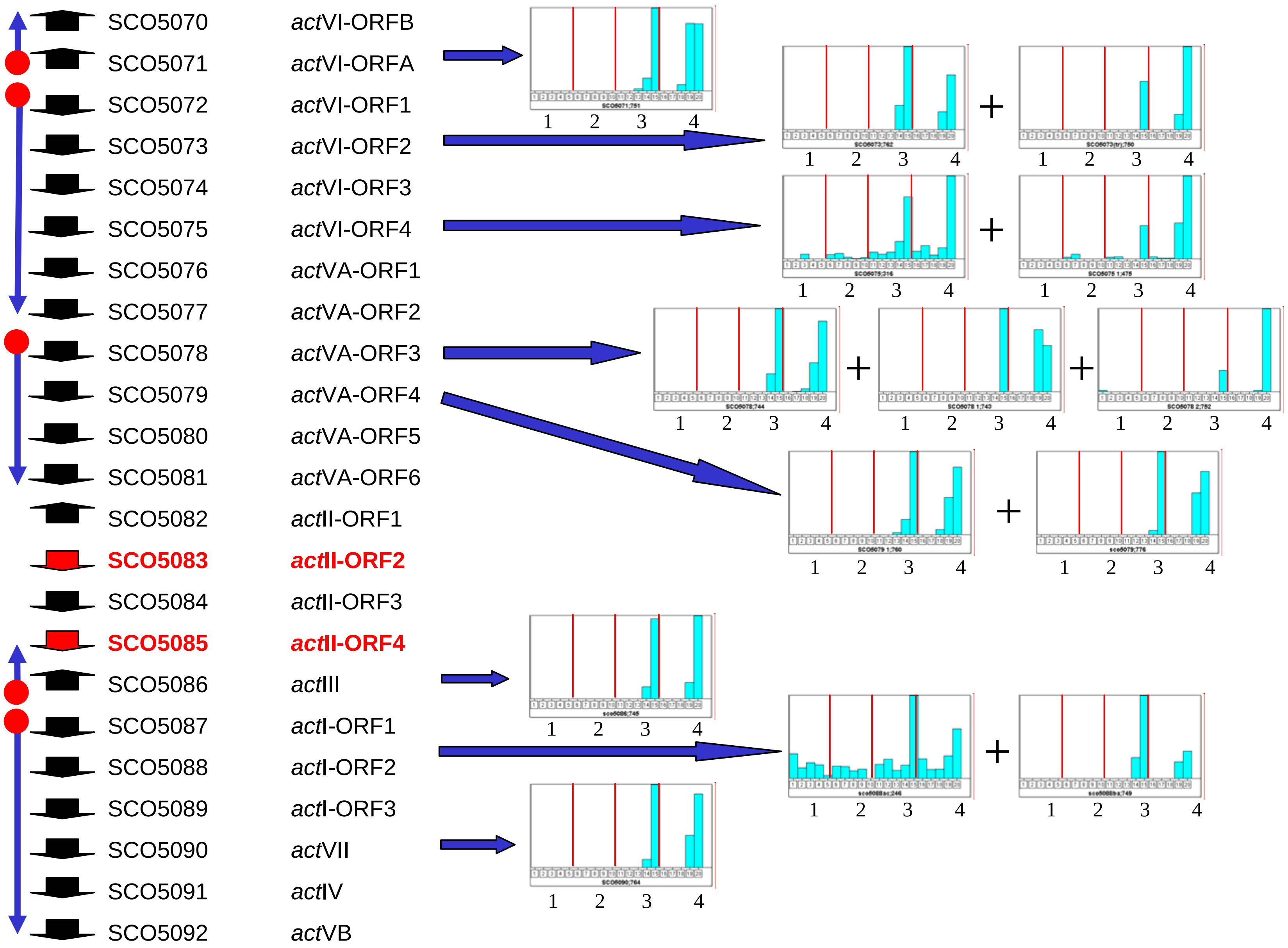

The *act* cluster: Illustration of five transcriptional units for genes encoding enzymes in the actinorhodin biosynthetic pathway, and summary of proteomics data showing decreased abundance of many proteins in the *bldA* mutant. Genes in red contain TTA codons. Panels 1 and 2 correspond to *bldA* replicates B and A respectively, while panels 3 and 4 similarly correspond to M600 replicates B and A. In each panel the earliest time point is on the left, and latest on the right.

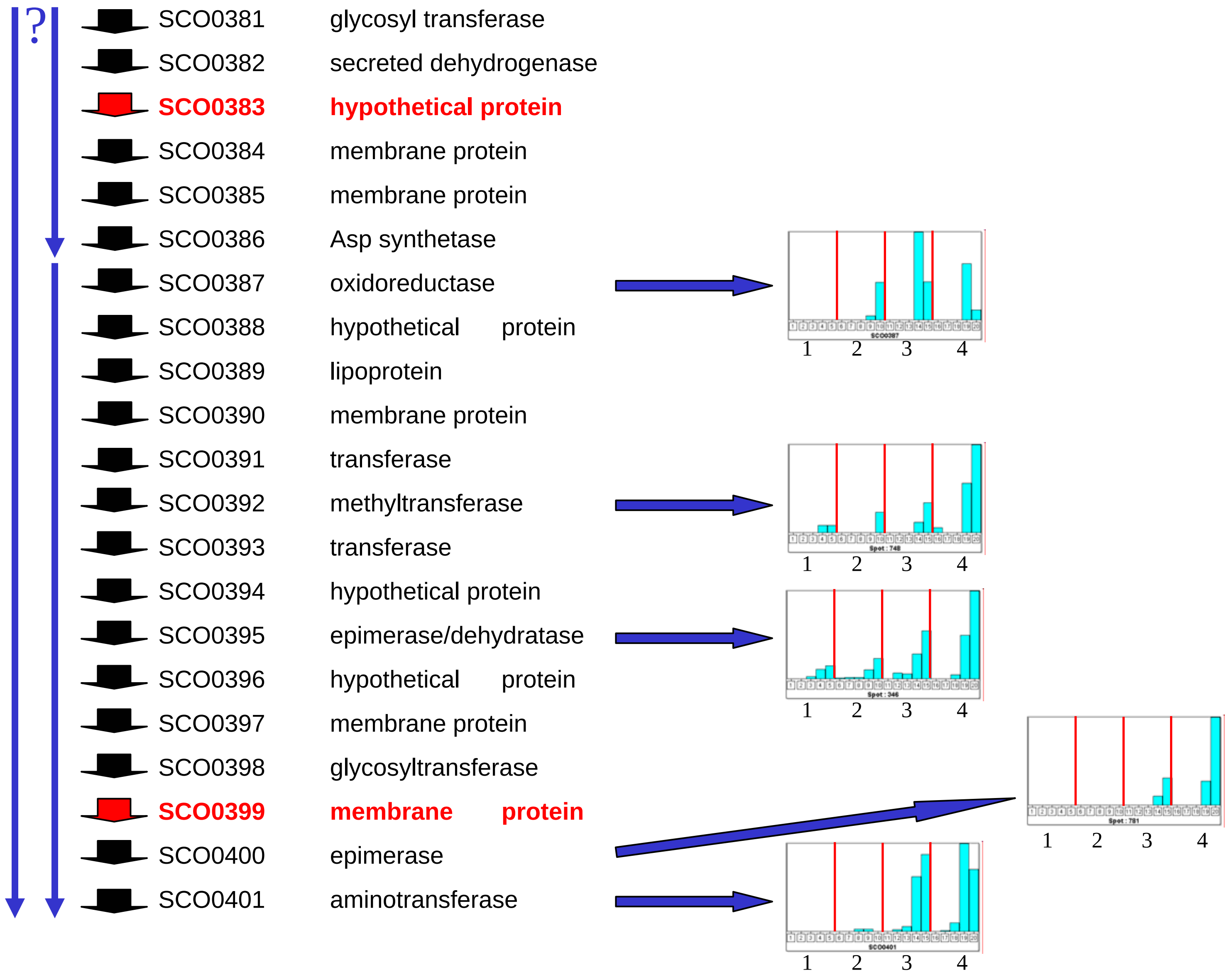

The deoxysugar/glycosyltransferase cluster: Illustration of putative transcriptional units for genes in the cluster, and summary of proteomics data showing decreased abundance of many proteins in the *bldA* mutant. Genes in red contain TTA codons. Panels 1 and 2 correspond to *bldA* replicates B and A respectively, while panels 3 and 4 similarly correspond to M600 replicates B and A. In each panel the earliest time point is on the left, and latest on the right.

CDA: SCO3236

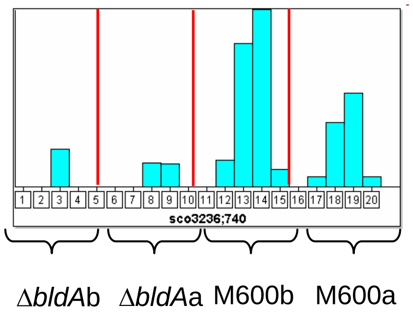

Red: SCO5895 (RedI)

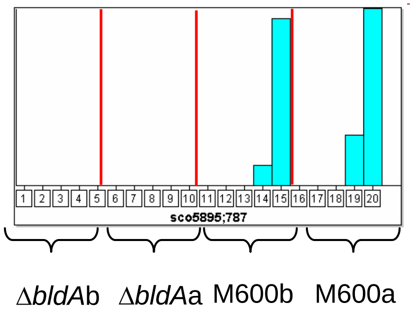

Type III polyketide synthase: SCO7221:

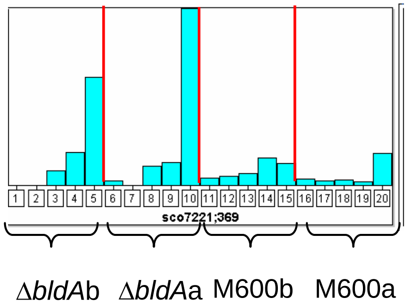

Differentially represented proteins from the CDA and Red antibiotic biosynthetic clusters, and the Type III polyketide synthase SCO7221.

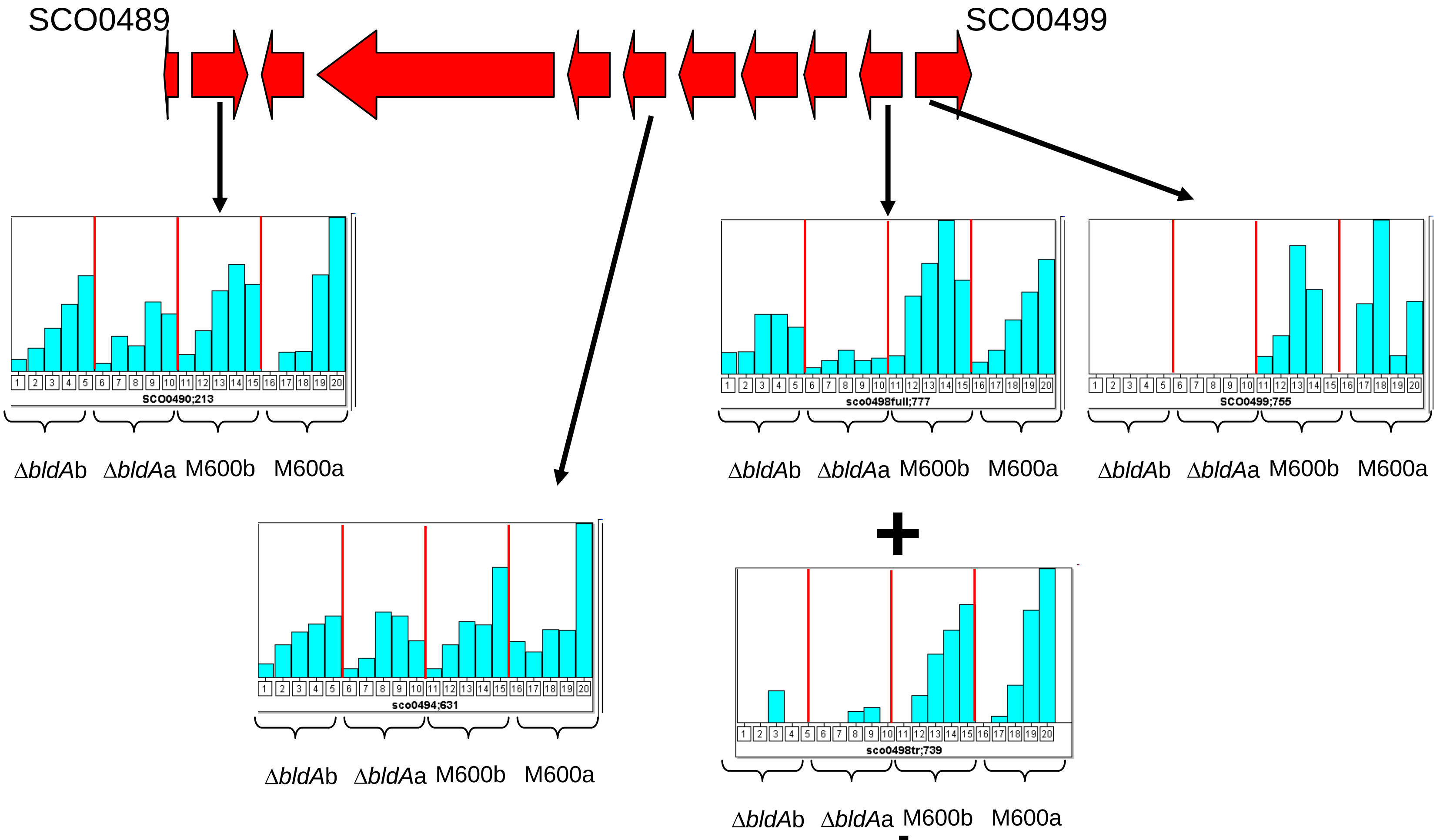

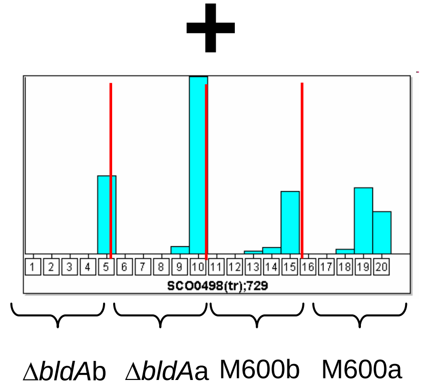

The coelichelin biosynthetic cluster illustrating differential representation of the SCO0498 and 0499 proteins in Δ*bldA*, but not the SCO0490 nor SCO0494 proteins

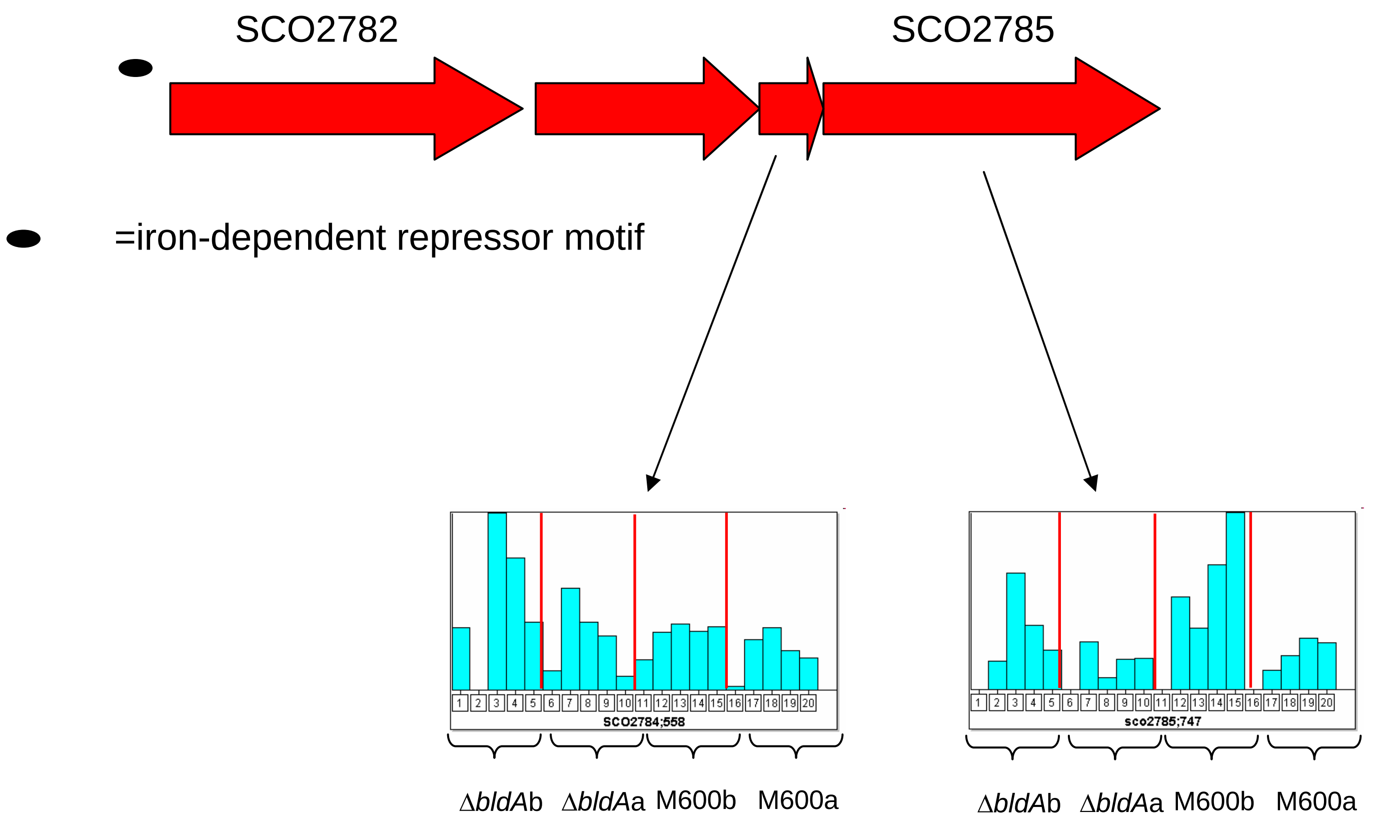

The desferrioxamines biosynthetic cluster illustrating subtle differential representation of the SCO2784 and 2785 proteins as a result of bldA mutation.
